# Weaning age influences the severity of gastrointestinal microbiome shifts in dairy calves

**DOI:** 10.1038/s41598-017-00223-7

**Published:** 2017-03-15

**Authors:** S. J. Meale, S. C. Li, P. Azevedo, H. Derakhshani, T. J. DeVries, J. C. Plaizier, M. A. Steele, E. Khafipour

**Affiliations:** 1 UMR Herbivores, INRA, Vetagro Sup, 63122 Saint-Genès-Champanelle, France; 2 Department of Animal Science, University of Manitoba, Winnipeg, MB Canada; 3 Department of Animal Biosciences, University of Guelph, Guelph, ON Canada; 4 Department of Agricultural, Food and Nutritional Science, University of Alberta, Edmonton, AB Canada; 5 Department of Medical Microbiology, University of Manitoba, Winnipeg, MB Canada

## Abstract

Ruminants microbial consortium is responsible for ruminal fermentation, a process which converts fibrous feeds unsuitable for human consumption into desirable dairy and meat products, begins to establish soon after birth. However, it undergoes a significant transition when digestion shifts from the lower intestine to ruminal fermentation. We hypothesised that delaying the transition from a high milk diet to an exclusively solid food diet (weaning) would lessen the severity of changes in the gastrointestinal microbiome during this transition. β-diversity of ruminal and faecal microbiota shifted rapidly in early-weaned calves (6 weeks), whereas, a more gradual shift was observed in late-weaned calves (8 weeks) up to weaning. Bacteroidetes and Firmicutes were the most abundant ruminal phyla in pre- and post-weaned calves, respectively. Yet, the relative abundance of these phyla remained stable in faeces (P ≥ 0.391). Inferred gene families assigned to KEGG pathways revealed an increase in ruminal carbohydrate metabolism (P ≤ 0.009) at 9, compared to 5 weeks. Conversely, carbohydrate metabolism in faeces declined (P ≤ 0.002) following a change in weaning status (i.e., the shift from pre- to post-weaning). Our results indicate weaning later facilitates a more gradual shift in microbiota and could potentially explain the negative effects of early-weaning associated with feeding a high-plane of pre-weaning nutrition.

## Introduction

The progressive microbial colonization of the ruminant intestinal tract begins at birth with the random acquisition of microbes from the animals’ surroundings. A number of factors may influence gastrointestinal microbial colonization including the cow’s vaginal microbiome, colostrum microbiota and co-inhabitance with other calves. Colostrum and milk harbor a rich microbial consortium imparting the gastrointestinal tract with beneficial microbes for metabolism and immune development, much like that in the human^1^. However, in young calves milk bypasses the rumen following activation of the esophageal groove^2, 3^, resulting in the initial acquisition of nutrients from the intestinal tract.

Activation of ruminal fermentative processes commences with the introduction of solid feed into the diet, with a dramatic shift occurring when milk is completely removed from the diet (weaning), as the source from which an animal attains its nutrition shifts, greatly altering the composition of the ruminal and intestinal microbiomes^4^. The resulting ruminal microbial fermentation products are essential for the development of the rumen wall papillae, providing niche environments^5, 6^, which aid the successional microbial colonization of the rumen with age. Early research using culture-based microbiology showed that aerobic and facultative anaerobic microbiota are gradually replaced by exclusively anaerobic taxa up to 6 and 8 weeks of age^7, 8^. This was confirmed by recent studies characterising the natural progression of the ruminal microbiome towards that of a mature ruminant^9^, even when fed a solely milk replacer diet^10^, suggesting the rumen and, thus, the intestine develop temporally to prepare for solid feed ingestion, but also indicates stimulation of ruminal development extends beyond just solid feed intake.

The stressful weaning transition has previously been linked with depressions in growth and intake. However, we recently showed that the age at which an animal is weaned can lessen these effects^11^, possibly a result of greater ruminal and intestinal maturation at 8, compared to 6 weeks of age. Considering this, in the current study, we investigated whether weaning age is a determining factor in the extent of microbial shifts in the rumen and faeces during weaning to better understand why calf growth slows during the weaning transition. We hypothesised that earlier weaned calves (6 week of age) would show a more rapid transition of ruminal and faecal microbiota, compared to calves weaned later in life (8 weeks of age), increasing the severity of this transition, potentially explaining the greater growth depressions observed throughout weaning of calves fed a high plane of pre-weaning nutrition.

## Results

### Alpha-diversity Measures

Richness of rumen microbiota, as indicated by the Chao1 index, was not affected by the interaction of weaning age x calf age (P = 0.24; Table 1). Additionally, the microbiota of all calves displayed similar evenness (Shannon and Inverse Simpson) across all ages, with the exception of early week 5 and late week 7 calves, which both had a greater (P ≤ 0.047) evenness than ruminal microbiota of early week 9 calves. Phylogenetic diversity (PD) of ruminal microbiota was greater (P = 0.01) in pre-weaned calves, compared to post weaned calves, with the exception of late-weaned week 5 calves which only showed a tendency (P = 0.06) to differ from late-weaned week 9 calves. Conversely, α-diversity of faecal microbiota was not affected by the interaction of weaning age x calf age or weaning age (P ≥ 0.10; Table 1). As expected, all indices were effected by calf age, where Chao1 was greater (P = 0.04) in calves at 7 weeks, compared to 5 weeks of age. Shannon, Inverse Simpson and PD were lower (P < 0.001) in faecal microbiota of calves at 5 weeks, compared to both 7 and 9 week old calves, which were similar (P > 0.05; Table 1).

**Table 1:** Alpha diversity indices.

	Calf age (weeks)						SEM	P-value		
	Early-weaned			Late-weaned						
	5	7	9	5	7	9		Weaning age	Calf age	Weaning age * Calf age
Rumen										
Chao1	1065	914	883	981	1009	938	55.1	0.70	0.18	0.24
Shannon	4.1	3.5	3.2	3.8	3.9	3.4	0.15	0.22	<0.001	0.05
Inverse Simpson	23.3	13.0	9.8	16.1	19.2	13.0	2.81	0.72	0.02	0.03
Phylogenetic diversity	48.2	35.7	34.1	45.7	45.4	38.5	2.03	0.01	<0.001	0.01
Faeces										
Chao1	2054	2320	2601	2058	2945	2236	311	0.86	0.04	0.30
Shannon	4.2	5.0	5.1	3.9	4.7	4.9	0.18	0.11	<0.001	0.80
Inverse Simpson	18.8	44.8	51.9	18.7	25.9	40.9	5.42	0.10	<0.001	0.32
Phylogenetic diversity	52.1	66.3	69.9	46.1	62.6	64.1	4.12	0.17	<0.001	0.94

### OTU Diversity and Similarity Analysis

**Figure 1:**
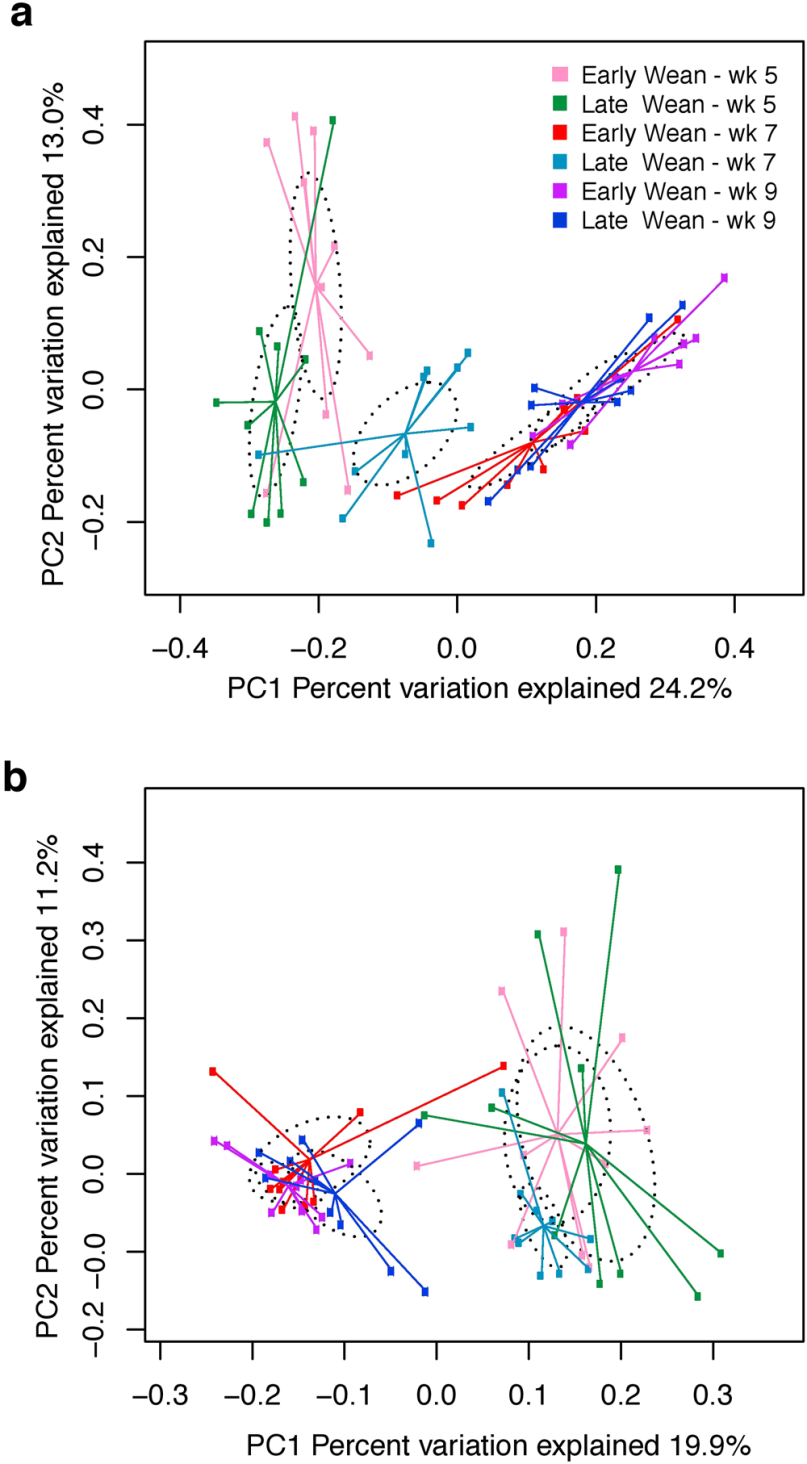
Similarities of ruminal and faecal microbiota compared across weaning age [early-weaned (Wk 6) vs. late-weaned (Wk 8)] and calf age (Wk 5, Wk 7 and Wk 9). Distance between the samples, based on similarity in OTU composition (OTU similarity ≥97%) calculated using weighted UniFrac distance in the a) rumen, and b) faeces, were visualised in principal coordinates analysis (PCoA) plots. A greater distance between two points infers a lower similarity. The impact of weaning age × calf age on the clustering pattern of microbial communities was tested using PERMANOVA (implemented in PRIMER-6 software; significance was declared when P < 0.05). Within the rumen, weaning age × calf age differed (P = 0.035) across all ages and groups, with the exception (P > 0.05) of early- versus late-weaned calves at 9 weeks of age, which were similar. Weaning age × calf age also differed (P = 0.004) in the faeces, with the exception (P > 0.05) of early- versus late-weaned calves at 5 and 9 weeks of age, and in early-weaned calves aged 7, compared to 9 weeks of age.

Community OTU comparisons were visualised by PCoA analysis (OTU ≥ 97% identity, species level similarity) using weighted (Fig. 1) and unweighted (Fig. S1) UniFrac distance metrics revealing a rapid shift in both ruminal and faecal microbiota of early-weaned calves at the time of weaning, compared to a more gradual shift in late-weaned calves (P ≤ 0.005; PERMANOVA), as evidenced by the proximity of early-weaned week 7 calves to that of post-weaned calves (early and late week 9 calves; Fig. 1), compared to the intermediate cluster of late week 7 calves between that of other pre-weaned calves (early and late week 5) and all post-weaned calves. In contrast to late-weaned calves, where faecal communities shifted (P = 0.004) between each age, faecal microbiota of early-weaned calves shifted (P < 0.002) at weaning and then remained stable (P = 0.257) in post-weaned calves regardless of their age. Interestingly, ruminal and faecal microbiota were similar across early- and late-weaned calves at 9 weeks of age (P ≥ 0.280).

### Bacterial Composition of Rumen and Faecal Microbiota

**Figure 2:**
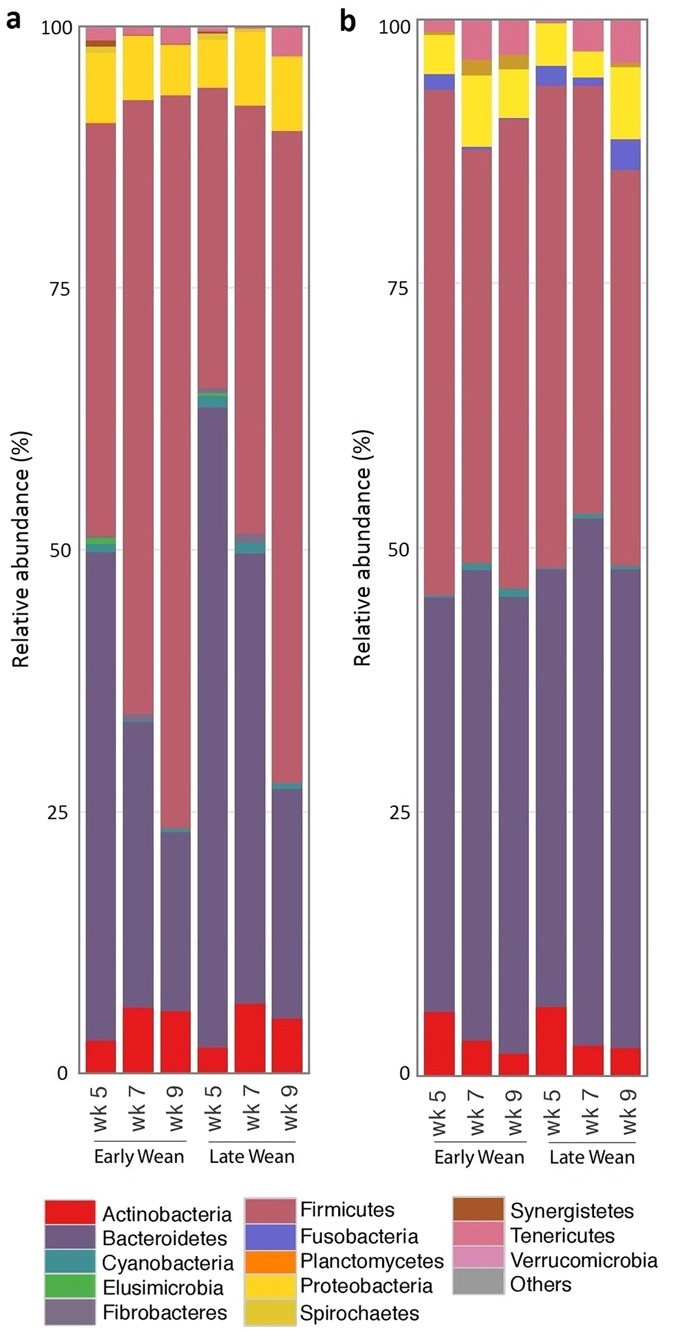
Phylum level composition. Bar plots showing average relative abundance of bacterial phyla (%) in (**a**) rumen and (**b**) faeces of early- (week 6) and late-weaned (week 8) calves for each weaning age.

The number of input 16S rRNA sequence reads generated from rumen and faecal microbiota were 26 772 ± 2 651 and 26 605 ± 4 503 (mean ± SD), respectively. While the majority of OTUs were identified at the genus (g.) level, some were only classified at the phylum (p.), class (c.), order (o.), or family (f.) level. Phylogenetic analysis of these sequences identified 24 phyla from the rumen, 12 of which had a relative abundance >0.1% of the community. The most abundant phyla in the rumen of pre-weaned calves was Bacteroidetes, which decreased (P ≤ 0.039) from week 5 to 7 in all calves, regardless of treatment, and also showed differences (P < 0.001) between week 5 and 9 calves, but remained stable (P ≥ 0.112) thereafter in all calves, despite their weaning status (Fig. 2; Table S1). In contrast, the relative abundance of p. Firmicutes increased (P = 0.017) in early-weaned calves from week 5 to 9 and increased (P ≤ 0.014) in late-weaned calves after weaning, making it the most abundant ruminal phyla in all post-weaned calves (Fig. 2; Table S1). Despite different weaning ages, the magnitude of change at weaning in these two ruminal phyla was similar across all calves (P = 0.969). Additionally, the ratio of Bacteroidetes to Firmicutes in the rumen decreased (P < 0.001) by 61 vs. 67% at the respective times of weaning in early- and late-weaned calves, respectively. However, no difference (P > 0.05) between the two weaning groups was observed in this ratio at the time of weaning. Relative abundances of p. Proteobacteria, p. Actinobacteria and p. Planctomycetes were similar across all calves, regardless of weaning age or calf age, averaging 6.03%, 4.96% and 0.01% of total sequences, respectively (Fig. 2; Table S1). Several other phyla were affected (P ≤ 0.035) by weaning in early-weaned calves including p. Cyanobacteria, p. Spirochaetes, p. Elusimicrobia, p. Synergistetes, p. Verrucomicrobia and p. Fibrobacteres, where all except the later decreased in calves at 7, compared to 5 weeks of age. Similarly, p. Elusimicrobia, and p. Fibrobacteres decreased (P ≤ 0.02) in late-weaned calves at the time of weaning, whereas, p. Tenericutes increased (P = 0.014) post-weaning in late-weaned calves.

Weaning resulted in faecal phyla shifting from the dominant p. Firmicutes in pre-weaned calves to p. Bacteriodetes in post-weaned calves (Fig. 2; Table S2). In early-weaned calves, dominance shifted again in 9 week old calves, returning to Firmicutes as the dominant phyla. Despite these shifts in dominance, weaning age did not affect (P ≥ 0.54) faecal abundance of p. Firmicutes or p. Bacteroidetes. The ratio of Bacteroidetes to Firmicutes in the faeces showed differences to that in the rumen, where a 25% reduction was observed between week 5 and 7 for all calves (1.16 to 0.87 and 1.07 to 0.81 for early- and late-weaned calves, respectively), followed by an increase of 19 and 6% from week 7 to 9. Relative abundances of p. Tenericutes and p. Cyanobacteria were greater (P ≤ 0.002) in late week 7 and 9, compared to late week 5 calves, but showed no differences (P ≥ 0.059) in early-weaned calves. Comparatively, p. Spirochaetes was more abundant (P < 0.001) in late week 9 calves, compared to late week 5 and 7 calves, and was greater (P = 0.021) in early week 9 calves, compared to early week 5 calves. Similarly, p. Fusobacteria was greater (P = 0.046) in early week 9 calves, compared to early week 5 calves, yet showed an increase (P = 0.001) from week 7 to week 9 in late-weaned calves. The relative abundances of p. Proteobacteria, p. Actinobacteria, p. Elusimicrobia and p. Verrucomicrobia were similar (P ≥ 0.08) across all calves and ages.

**Figure 3:**
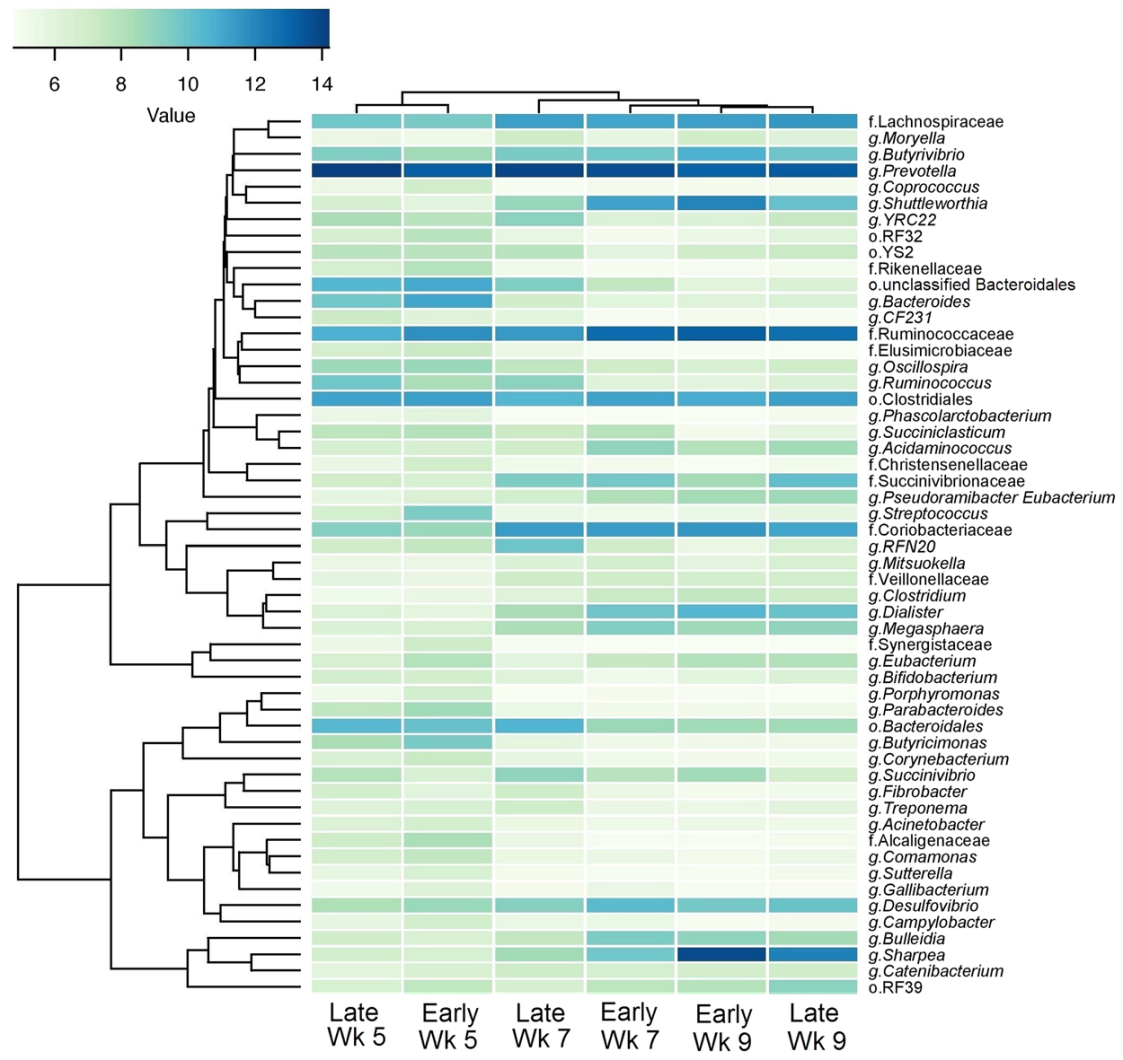
Correlation between ruminal microbiota and calf age and weaning strategy. The heatmap shows bacterial abundance in rumen varied with calf age (5, 7 and 9 weeks) and weaning age (6 vs. 8 weeks for early- and late-weaning, respectively). Data represent taxa present at greater than 1% of community that were significantly different (P < 0.05) between at least two ages or between treatment groups (weaning age). See Table S1 for additional data. The OTU count data was subjected to variance stabilizing transformation and summarised to calculate genus abundant data. Hierarchically clustering was then performed based on complete linkage of Euclidean distances matrix and visualized over heatmap using the heatmap.2 function of the “gplots” R package.

Of the 312 genera observed in the rumen, 56 had a relative abundance over 1%, and were affected either by weaning age or calf age (Fig. 3; Table S1). The relative abundance of the dominant g. *Prevotella* remained stable (P ≥ 0.28) in the rumen of early- and late-weaned calves. The most abundant genera became *Sharpea* in both early- and late-weaned calves at 9 weeks of age following an increase (P < 0.001) as a result of weaning (week 5 vs. 7) in early-weaned calves, and an additional increase (P < 0.001) in the relative abundance from week 7 to 9 of life. Conversely, no change (P = 0.90) occurred in the relative abundance of g. S*harpea* until weaning in late-weaned calves, where an increase was observed (P < 0.001; Fig. 3 and Table S1). The relative abundance of g. *Ruminococcus* decreased (P < 0.001) at weaning in early-weaned calves and remained stable (P ≥ 0.64) thereafter, and only showed a reduction (P < 0.001) between week 5 and week 9 late-weaned calves. The time of weaning did not affect the size of this shift (P = 1.00; Table S1). Additionally, weaning at 6 weeks (early-weaning) caused a sharp increase (P < 0.001) in the relative abundance of g. *Shuttleworthia* at weaning, remaining stable thereafter, whereas late-weaned calves exhibited only a tendency (P = 0.06) to increase from week 5 to 9 (Fig. 3 and Table S1).

**Figure 4:**
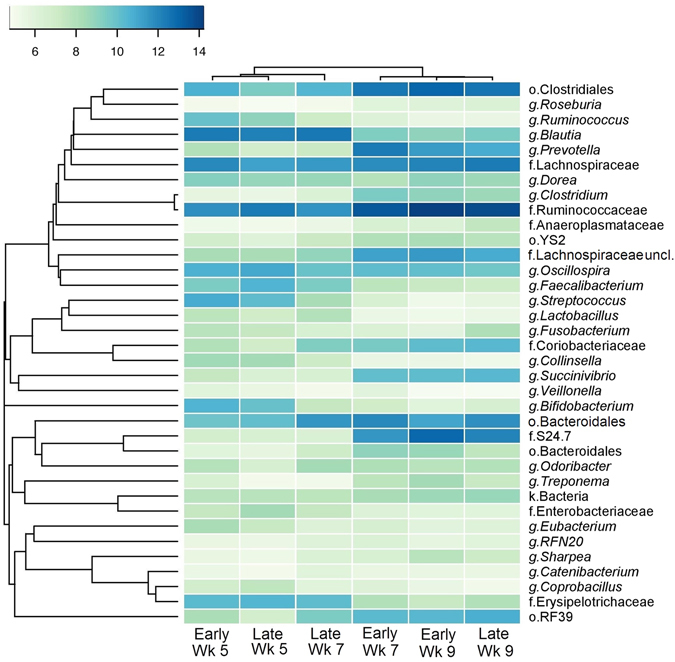
Correlation between faecal microbiota and calf age and weaning strategy. The heatmap shows bacterial abundance in faeces varied with calf age (5, 7 and 9 weeks) and weaning age (6 vs. 8 weeks for early- and late-weaning, respectively). Data presented represent taxa present at greater than 1% of community that were significantly different (P < 0.05) between at least two ages or between treatment groups (weaning age). See Table S2 for additional data. The OTU count data was subjected to variance stabilising transformation and summarised to calculate genus abundant data. Hierarchically clustering was then performed based on complete linkage of Euclidean distances matrix and visualised over heatmap using the heatmap.2 function of the “gplots” R package.

A total of 190 genera were identified in faecal microbiota, 46 of which were present at >1% of the total sequences and showed a difference relative to either weaning age of calf age (Fig. 4; Table S2). The relative abundance of g. *Bacteroides* was dominant and stable (P = 1.00; Table S2) across all ages regardless of weaning age, averaging 25.1% of total sequences. *Blautia* was the second most abundant genera in pre-weaned calves, regardless of weaning age. Relative abundance of this genera declined (P < 0.001) at weaning; however, the magnitude of this decrease was similar despite different weaning ages (av. 84.8% decrease; P = 1.00; Fig. 4 and Table S2). Conversely, an unclassified genus in f. *Ruminococcaceae* was the second most abundant taxa in post-weaned calves following similar increases (P ≤ 0.036) at weaning, regardless of weaning age.

### Correlation between Rumen and Faecal Microbiota and Calves Physiological Variables

**Figure 5:**
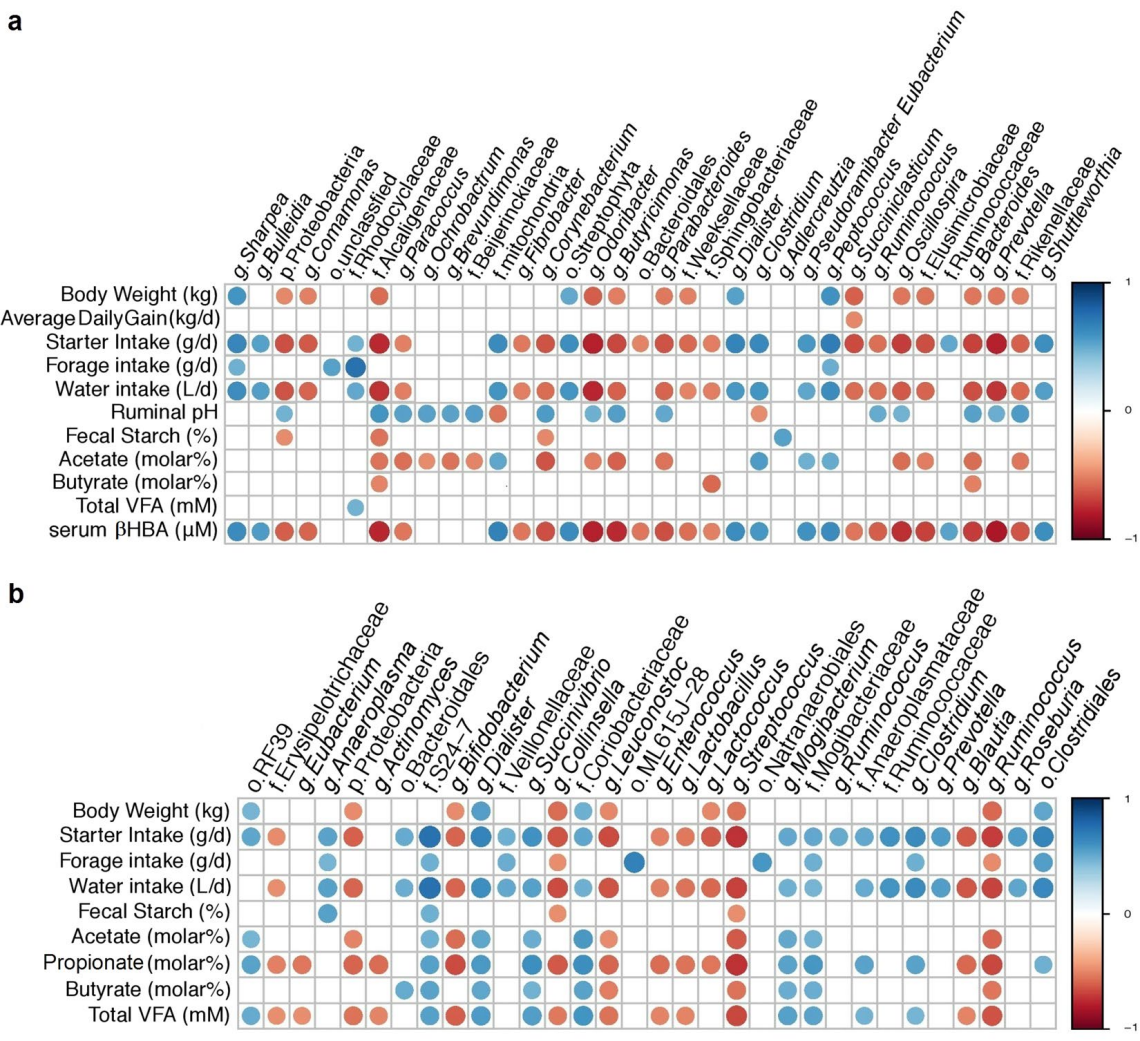
Correlations between production variables and relative taxa abundance. Spearman non-parametric rank correlation matrix of the dominant bacterial genera across (**a**) rumen and (**b**) faecal samples. The genera were included in the matrix if they were in at least 50% of calves and represented at least 0.1% of the bacterial community in at least one calf. All correlations presented were statistically significant (P < 0.05), with strong correlations indicated by large circles and weaker correlations indicated by small circles. The scale colours denote whether the correlation is positive (closer to 1, blue circles) or negative (closer to −1, red circles) between the taxa and production variables. The OTU count data was subjected to variance stabilising transformation then pairwise Spearman correlations between bacterial genus and biological parameters at corresponding ages were analysed. P-values were adjusted for multiple comparisons.

Of the genera detected in ruminal and faecal microbiota, the relative abundance of those present in at least 50% of calves and represented at least 0.1% of the bacterial community in at least one calf, were compared to calf physiological variables and rumen fermentation characteristics (Fig. 5). The relative abundance of g. *Prevotella* in the rumen was negatively correlated (P ≤ 0.021) with calf body weight (Spearman’ ρ = −0.53), starter intake (Spearman’ ρ = −0.77) and serum βHBA (Spearman’ ρ = −0.81) and positively correlated with ruminal pH (Spearman’ ρ = 0.49; Fig. 5a). Conversely, in the faeces, g. *Prevotella* was positively correlated (Spearman’ ρ = 0.56; P = 0.001) with starter intake, but showed no correlation with body weight (Fig. 5b). Similarly, g. *Dialister* in both rumen and faeces were positively correlated (P ≤ 0.002) with calf physiological variables including body weight (Spearman’ ρ = 0.55; Spearman’ ρ = 0.55) and starter intake (Spearman’ ρ = 0.67; Spearman’ ρ = 0.66), respectively, and serum βHBA (Spearman’ ρ = 0.63) with ruminal g. *Dialister*.

Similarly, g. *Sharpea* in the rumen was positively correlated (P ≤ 0.001) with body weight (Spearman’ ρ = 0.59), starter intake (Spearman’ ρ = 0.66) and serum βHBA (Spearman’ ρ = 0.64) and, in addition, showed a positive correlation (P = 0.03) with forage intake (Spearman’ ρ = 0.48). Conversely, no correlation (P > 0.05) between the relative abundance of g. *Sharpea* in faeces and calf physiological variables was observed.

### Metabolic Functions and Capacity of Ruminal and Faecal Microbiomes

**Figure 6:**
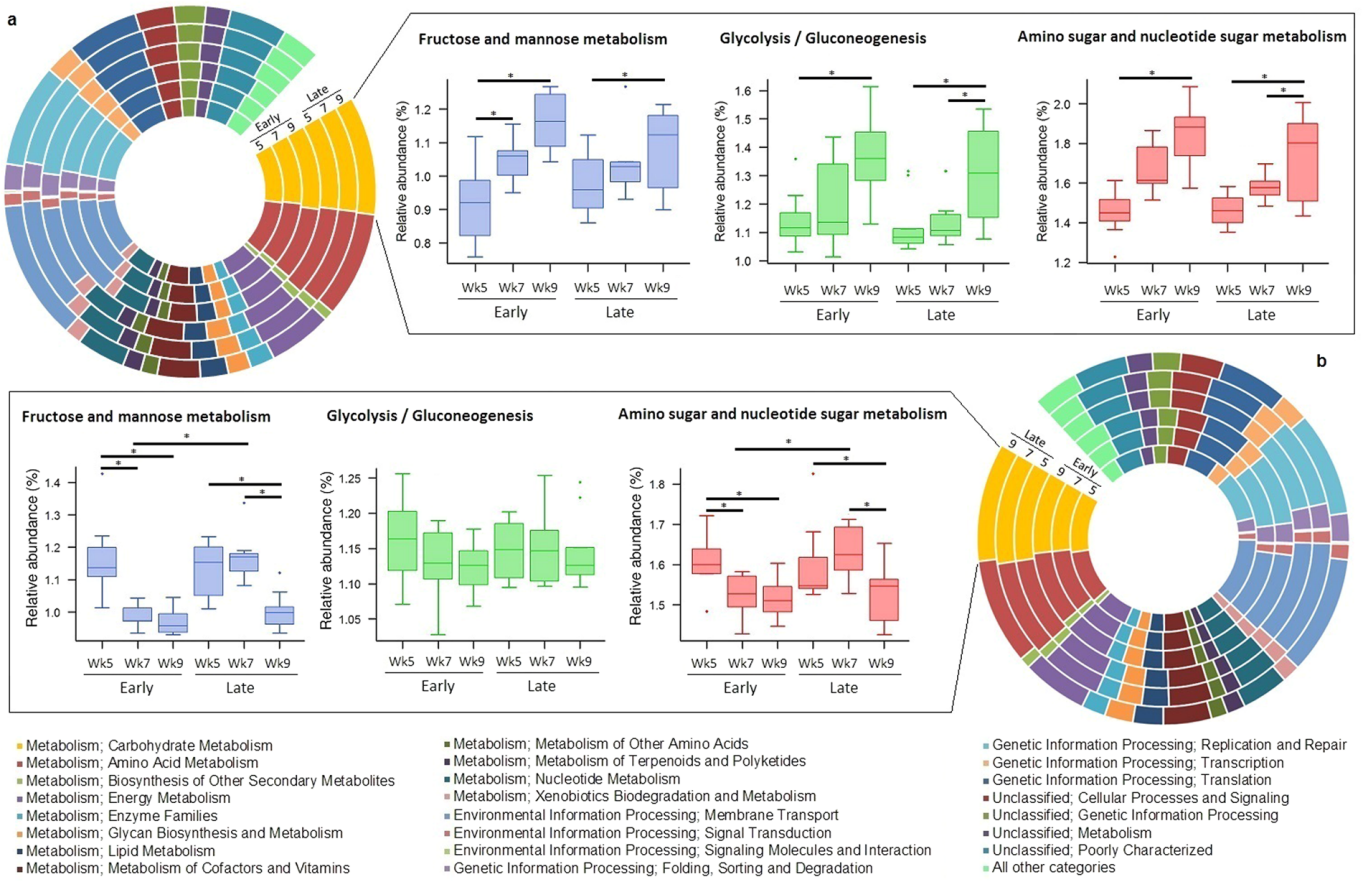
PICRUSt predicted summary of COG categories from 16S amplicon sequencing of (**a**) ruminal and (**b**) faecal microbiomes. Relative abundances of Level 2 KEGG pathways are depicted by calf age within treatment group (weaning age) in circular barplots. Relative abundances of the three most abundant L3 KEGG pathways involved in Carbohydrate Metabolism (Fructose and Mannose Metabolism, Glycose/gluconeogenesis and, Amino sugar and nucleotide sugar Metabolism) at different ages (5, 7, or 9) subjected to weaning at either 6 weeks (Early) or 8 weeks (Late) of age are also presented within the boxes. *P < 0.05 for the shown comparisons of L3 pathways (See Supplementary Tables 3–6 for complete KEGG data).

To assess the metabolic potentials of ruminal and faecal microbiomes, OTUs were entered into PICRUSt and the inferred gene families were annotated against KOs then collapsed into KEGG pathways. Gene families annotated to bacterial carbohydrate metabolism increased (P ≤ 0.009) in the rumen of all calves at 9 weeks of age, compared to 5 weeks of age (Fig. 6; Table S3). Conversely, the number of gene families annotated to bacterial carbohydrate metabolism in faeces only declined (P ≤ 0.002) following a change in weaning status (i.e., pre- vs. post-weaning) in both early- and late-weaned calves (Fig. 6; Table S4). The relative abundance of KEGG L2 pathways associated with metabolism of amino acids, energy or lipids remained stable (P ≥ 0.114) in ruminal bacteria across all calf ages, regardless of weaning age. Similarly, the abundance of genes associated with lipid metabolism was stable (P ≥ 0.201) in the faeces of all calves. However, the abundance of genes associated with both energy and amino acid metabolism tended to be lower (P ≤ 0.059) in faeces of early-weaned week 7, compared to early-weaned week 5 calves. Additionally, genes annotated to amino acid metabolism showed a tendency to decrease (P = 0.094), and decreased (P ≤ 0.036) in faeces of calves at 9 weeks, compared to those at 5 weeks of age, across early- and late-weaned calves, respectively.

A total of 328 KEGG level 3 pathways were identified in the rumen and 266 from faeces (Tables S4 and S5). Of the OTU inferred gene families assigned to KEGG pathways in the rumen, the three most abundant related to carbohydrate metabolism that changed in both early- and late-weaned, included amino sugar and nucleotide sugar metabolism (hsa00520), glycolysis/gluconeogenesis (hsa00010), fructose and mannose metabolism (hsa00051; Fig. 6). Fructose and mannose metabolism (P ≤ 0.002) increased following weaning in early-weaned calves. However, only a difference was observed between calves at week 5 and week 9 in late-weaned calves. Glycolysis/gluconeogenesis and amino sugar and nucleotide sugar metabolism were greater (P ≤ 0.01) in calves at 9 weeks of age, compared to 5 or 7 weeks for late-weaned calves. Yet, early-weaned calves at 9 week of age showed a greater (P ≤ 0.014) abundance of OTUs assigned to glycolysis/gluconeogenesis and amino sugar and nucleotide sugar metabolism, compared to those aged 5 weeks, with those aged 7 weeks intermediary between week 5 and week 9.

Comparatively, in the faecal microbiome, of the inferred gene families assigned to KEGG pathways, the three most abundant related to carbohydrate metabolism were the same as that of the rumen, with amino sugar and nucleotide sugar metabolism, and fructose and mannose metabolism decreasing (P < 0.006) following the respective weaning of both early- and late-weaned calves (Fig. 6). Glycolysis/gluconeogenesis showed a tendency to decrease following weaning in early-weaned calves (P ≤ 0.082), but remained stable (P ≥ 0.482) in late-weaned calves (Fig. 6).

## Discussion

Recently, we showed that weaning age (6 vs. 8 weeks of age) differentially affects physiological development of a calf fed a high plane of pre-weaning nutrition (>8 L/d), including growth during the transitional period, where later weaning promoted beneficial effects on growth and intake during weaning and thereafter^11^. Early studies suggested that the natural transition to strictly anaerobic taxa in the rumen ceases at around 6 to 8 weeks of age indicating that maturity of the ruminal and intestinal microbiomes at weaning may influence the impact this transition has on calf development and growth. Subsequently, in this study we sought to characterise the shift in ruminal and faecal microbiota, representative of the intestinal microbiome, which occurs across weaning in calves weaned at 6 and 8 weeks of age to elucidate if changes in the microbial structure could be responsible for the observed production effects (reported previously^11^).

A comparison of all calves showed that species richness (chao1 index) within the rumen and faeces were constant across all ages, regardless of calf weaning age (Table. 1). However, the microbial community in the rumen of calves weaned at an earlier age became less even, as determined by the Shannon index, between week 5 and 9 of age, a change which was not observed in late-weaned calves. Similarly, weaning calves at 6 weeks of age caused a rapid shift in β-diversity of rumen and faeces from a pre- to post-weaned state, evidenced by the similar diversity in those calves at week 7 and 9 (Fig. 1). Such a shift reflects the abrupt change in the source from which the calves obtained nutrients and was perhaps related to the pre-mature development of the rumen, paralleled by a reduction in calf growth (44 vs. 12% reduction for early- and late-weaned calves, respectively) observed by these calves during weaning^11^. Conversely, late-weaned calves displayed a gradual transition in β-diversity (Fig. 1) with calves at week 7 displaying an intermediate diversity between calves aged 5 and 9 weeks, reflecting the gradual increase in consumption of starter (Fig. S2) as calves aged and thus, a progressive development of the rumen. Interestingly, all calves at 9 weeks of age displayed similar diversity when using weighted UniFrac measurement; however, a difference was observed in the rumen of early- vs. late-weaned calves at 9 weeks of age when considering unweighted UniFrac (Fig. S1), which accounts for differences in unique evolutionary history of either group. This may indicate that although the microbial profile was similar, its functional potential may have evolved differently as a result of different weaning ages.

Weaning of calves shifted the dominant ruminal phyla from Bacteroidetes in pre-weaned calves, to Firmicutes in post-weaned calves, regardless of their age at weaning (Fig. 2). The abundance of p. Bacteroidetes; however, only declined between week 5 and 7, and remained stable thereafter. This corresponds to 2 month old pre-weaned calves fed milk replacer with access to calf starter where Bacteroidetes was also the dominant phyla^9^. Conversely, the increase in p. Firmicutes at the respective weaning times in our study, agrees with the increase observed between the abundance of p. Firmicutes in weaned-calves at 6 months of age, compared to pre-weaned calves at 2 months of age^9^. Similarly, Li *et al.* ^10^ observed a decline in the abundance of Bacteroidetes in pre-weaned calves to that of 1 year old post-weaned bull calves fed a hay diet and a corresponding increase in p. Firmicutes. These results, in conjunction with the abundance of p. Proteobacteria, concur with previous studies in cattle^4, 9^ which suggest that the developing rumen (pre-weaning) comprises the same three dominant phylum (Bacteroidetes, Firmicutes and Proteobacteria) as the more mature gut of post-weaned calves, although the relative abundances of each phyla vary with the stage of development^12^.

The dominance of g. *Prevotella* in the rumen of all calves was not unexpected, as this genus comprises species involved in degradation of non-structural carbohydrates and xylan^13^. Additionally, the stability of this genus across weaning is in agreement with Rey *et al.* ^14^, where the Bacteroidetes phylum was dominated by *Prevotella* in all calves older than 15 d of age when consumption of starter increased above 100 g/d, resulting in the acidification of the rumen^15^. Similarly, in our study, calves began consuming starter at 2 weeks of age with intakes averaging 83 g/d, and reaching 1307 and 504 g/d at week 7 for early- and late-weaned calves, respectively. Similarly, our results agree with those obtained in steers^10, 16^ and in adult dairy cows^16, 17^ suggesting that this genus is associated with diets containing solid food. Conversely, Li *et al.* ^18^ and Wu *et al.* ^17^ noted in calves fed solely milk replacer at 42 d of age that g. *Bacteroides* was dominant within the rumen, yet g. *Prevotella* was only present at low abundance rates. In accordance, within our study we observed a negative correlation between g. *Bacteroidetes* and starter intake, where g. *Bacteroides* decreased from week 5 to 7 in all calves, coinciding with the timing of starter consumption reaching above 100 g/d. However, calves weaned earlier displayed a greater reduction in g. *Bacteroides* at the time of weaning (6 weeks of age), compared to later-weaned calves (8 weeks of age), likely reflecting the greater shift in dietary composition from high milk replacer and low starter intakes to solely starter in early-weaned calves, compared to a more moderate starter and milk replacer diet consumed by late-weaned calves. Two additional ruminal genera which showed contrasting changes depending on the age at weaning included *Dialister* and *Shuttleworthia*, both of which increased dramatically in early-weaned calves, but showed no differences in late-weaned calves across weaning (Fig. 3). Collectively, these results indicate that, in agreement with earlier studies^19, 20^, the sooner a calf consumes solid feed, the sooner a ruminal bacterial community more representative of a mature rumen develops. However, the rumen may not be as capable of handling such dramatic changes in calves at 6 weeks of age, and could possibly account for the negative production effects observed in these calves.

The abundance of the two dominant faecal phyla Firmicutes and Bacteroidetes remained stable across weaning in all calves. This corresponds to a recent study in humans^21^, which explored the faecal microbiome as solid feed was introduced into the diet of babies fed breast milk or formula. The relative abundance of p. Firmicutes remained stable whereas, p. Bacteroidetes increased with solid feed consumption, as also reported by Koenig *et al.* ^22^. As the calves in the current study were already consuming solid feeds at the first sampling (64 vs. 103 g/d for early- and late-weaned calves, respectively) it is reasonable to conclude the shift in p. Bacteroidetes may have already occurred. Additionally, it has been suggested that the dietary balance between carbohydrates and protein or animal fat is the primary factor shifting faecal microbiota, where greater g. *Bacteroides* abundance is associated with high protein and animal fat diets, compared to g. *Prevotella* which predominates with greater carbohydrate intake^17, 21, 23^. In our study, the dominant genera within the faeces was *Bacteroides* which also remained stable across all calves. Additionally, g. *Blautia* from the f. Lachnospiraceae, which constitutes a major taxonomic group capable of degrading complex polysaccharides to short chain fatty acids including acetate, butyrate, and propionate in the human gut^24, 25^ was observed to decrease post-weaning in our study, compared to an increase observed following solid feed consumption in human babies^21^, likely reflecting the different digestive systems and the shift to ruminal fermentation in post-weaned calves.

The observed shifts in bacterial community have previously been noted to correspond to the nature of the fermentation substrate more so than the amount consumed^14^, with increases in solid feed correlating to increased production of short chain fatty acids (SCFA)^21, 22^. However, here we observed that despite being a producer of SCFA, ruminal abundance of g. *Bacteroides* was negatively correlated with acetate and butyrate concentration as well as calf body weight, starter intake and serum βHBA; however, no correlations were observed between *Bacteroides* and propionate concentration in the rumen. We observed a co-linearity between starter intake and water intake (R^2^ = 0.90), and starter intake and serum βHBA (R^2^ = 0.85; data not shown) in calves across all ages, providing a possible explanation for the strong association between bacterial taxa abundance and serum βHBA levels, and further suggests that in this study, the abundance of g. *Bacteroidetes* strongly relates to starter consumption. Furthermore, increasing serum βHBA levels in relation to increasing starter consumption is not surprising, as the end products of ruminal fermentation, SCFA, are absorbed and metabolised by ketogenesis in ruminal epithelium^26^.

Within the Firmicutes phyla, g. *Sharpea* increased in agreement with our previous work^4^ and showed a positive correlation to starter intake and serum βHBA (Fig. 5). A strong association between the ruminal abundance of *Sharpea* and methane was found recently, where lower emitting sheep had a higher abundance of g. *Sharpea* and an increased lactate production, which in turn, resulted in an increased conversion of lactate to butyrate by *Megasphaera* spp.^27^. Whist an association was not observed between ruminal *Sharpea* and fermentation parameters here, it is possible that such an increase in butyrate contributed to the observed overall increase in serum βHBA levels.

Similarly, ruminal and faecal abundances of g. *Dialister* were positively correlated with starter intake and calf body weight in the current study, corresponding to a study by Meyer *et al.* ^28^ which suggested that ruminal abundance of this genera were positively associated with average daily gain in beef steers, suggesting that perhaps this genera is responsive to body weight. Additionally, g. *Dialister* has been positively correlated with total VFAs and propionate, with the authors indicating this genera may be involved in VFA metabolism and starch degradation due to observed increases in amylase and carboxymethylcellulase activities^29^.

In addition to the observed changes in bacterial abundances, diet induced differences may also contribute to host metabolism and immune function by regulating genes involved in lipid and carbohydrate metabolism, altering endocrine functions, increasing inflammatory responses, and influencing energy balance and body weight^30^. Using predicted metagenomes (PICRUSt) of rumen and faeces microbiomes we inferred that there were several functional pathways associated with weaning (Fig. 6). Most notably, a significant reduction in genes associated with carbohydrate metabolism in the faecal microbiome following weaning mirrors the shift in nutrient metabolism away from the lower GI tract to ruminal fermentation, as previously seen in Meale *et al.* ^4^. However, increases in the abundance of genes associated with carbohydrate metabolism in the rumen only occurred in 9-week old calves, compared to those at 5 weeks, rather than with changes in weaning status. This suggests, in agreement with our previous study^4^, that consumption of starter initiated a shift in microbial taxa towards that of the mature rumen and potentially lessened any effects of a shift at weaning. Li *et al.* ^10^ noted that in the absence of solid feed, carbohydrate transport and metabolism, as indicated by the Clusters of Orthologous Groups (COG) classification, did not change with increasing age in pre-weaned calves suggesting that ingestion of solid feed is essential in developing the functionality of the rumen. The increase observed in calves at 9 weeks of age, indicates that perhaps the functional role of the rumen was still developing. It is possible that at this age the rumen is better developed to handle increasing consumption of starter and can regulate carbohydrate metabolism accordingly, providing some insights as to why calves weaned at 6 weeks of age exhibited a greater decline in growth through the weaning transition. Importantly, however, we must note that PICRUSt predictions are based only on known functions of the microbial communities present in the GI tract of humans and animals obtained from the whole genome shotgun sequencing of such samples. As there are limited numbers of shotgun sequencing studies in ruminants, the functionality of the members of ruminant GI microbiota may be over- or underestimated in PICRUSt.

In conclusion, our results suggest that weaning later results in a more gradual shift in microbial diversity in both the rumen and faeces of dairy calves and this shift, in conjunction with that observed in functional data, indicates an increased state of readiness for the weaning transition in calves weaned at 8 weeks of age; this may account for the lower declines in growth observed in these calves reported earlier^11^. Despite no changes in the relative abundance of dominant intestinal phyla, as represented by the faeces, across weaning at either weaning age, the gradual increase in solid feed consumption of late-weaned calves resulted in the rumen harbouring a microbial consortium sufficient to perform the major fermentative and metabolic functions prior to weaning, and as such, the level of physiological stress associated with the weaning transition was not as severe as observed in early-weaned calves.

## Methods

### Animal Experiment and Sample Collection

Experimental procedures used in this study were approved by the Nutreco Canada Agresearch Animal Care Committee in accordance with the Canadian Council on Animal Care guidelines^31^. A detailed description of the experimental treatments and growth measurements was published previously^11^. Briefly, 20 female Holstein dairy calves were randomly assigned at birth to be weaned at 6 (early) or 8 (late) weeks of age. Calves were fed Shur-Gain Optivia High Performance (26% CP, 16% fat; ME = 4.58 Mcal/kg) Milk Replacer (Nutreco Canada Inc., Guelph, ON, Canada), mixed at a rate of 150 g/L of 40 °C water twice daily (0730 and 1630 h) by nipple bottle (Super Calf Nipple). At d 7 ± 3 calves were transitioned to a gate-mounted artificial teat (Peach Teats; Skellerup Industries Ltd., Woolston, New Zealand), through which they were fed until weaning. Calves were offered 6 L from d 1 to 3, 7 L from d 4 to 6, and 8 L from d 7 until their respected time of step-down weaning. The early-weaning group was stepped down to 4 L/d on d 36 and weaned on d 43. The late-weaning group was stepped down to 4 L/d on d 50 and weaned on d 57 [see Eckert *et al.* ^11^ for figure]. Calves had *ad libitum* access to water, starter (Optivia Advantage Calf Starter; 22% CP; ME = 2.63 Mcal/kg), chopped straw (8% CP) and oat straw (8% CP; ME = 1.55 Mcal/kg) chopped to a length of 3 cm, from d 0–70. Orts were weighed and discarded daily.

Rumen and faecal samples were collected on d 35, 49 and 63 (Week 5, 7 and 9) of life. Rumen fluid was collected, on those same days, at 1100 h using a modified Geishauser oral probe (1.3 cm diameter)^11, 32^. The probe was inserted 50 cm inside the calf and pH measurements of all samples were above 5.0, indicative of the rumen not the abomasum. Samples were collected in 15 mL tubes, flash frozen in liquid nitrogen and stored at −80 °C. Calves were rectally finger-stimulated with sterile–gloved hand to facilitate the collection of a 50 g faecal sample, which was immediately frozen at −80 °C.

Calf body weight (BW), starter intake, straw intake, faecal starch concentrations, serum β-hydroxybutyrate (βHBA), behavioural observations, rumen acetate, butyrate and propionate, and total rumen volatile fatty acids (VFA) concentrations, reported previously^11^, were reinterpreted in the context of their correlations with microbial changes evaluated in this research.

### DNA Extraction

Rumen fluid and faecal samples were thawed at room temperature and kept on ice during the extraction process. Two hundred milligram faecal samples or the sediment collected from 1 mL rumen fluid by centrifuging at 15,000× *g* for 5 min was used for DNA extraction using a ZR faecal DNA kit (D6010; Zymo Research Corp., Irvine, CA, USA) that included a bead-beating step for mechanical disruption of microbial cells. DNA was eluted from the column with elution buffer, and DNA concentration was quantified using a NanoDrop 2000 spectrophotometer (Thermo Scientific, Waltham, MA, USA). DNA samples were normalized to 50 ng/µL, and quality verified by PCR amplification of the 16S rRNA gene using universal primers 27 F (5′-GAAGAGTTTGATCATGGCTCAG-3′) and 342 R (5′-CTGCTGCCTCCCGTAG-3′), as described before^33^. Amplicons were verified by agarose gel electrophoresis. All DNA samples were stored at −80 °C.

### Library Construction and Illumina Sequencing

The V4 region of 16S rRNA gene was targeted for PCR amplification using modified F515/R806 primers^34^ as described before^35^. The reverse PCR primer was indexed with 12-base Golay barcodes allowing for multiplexing of samples. PCR reaction for each sample was performed in duplicate and contained 1.0 µL of pre-normalized DNA, 1.0 µL of each forward and reverse primers (10 µM), 12 µL HPLC grade water (Fisher Scientific, Ottawa, ON, Canada) and 10 µL 5 Prime Hot MasterMix (5 Prime, Inc., Gaithersburg, USA). Reactions consisted of an initial denaturing step at 94 °C for 3 min followed by 35 amplification cycles at 94 °C for 45 sec, 50 °C for 60 sec, and 72 °C for 90 sec; finalized by an extension step at 72 °C for 10 min in an Eppendorf Mastercycler pro (Eppendorf, Hamburg, Germany). PCR products were then purified using ZR-96 DNA Clean-up Kit (ZYMO Research, Irvine, CA, USA) to remove primers, dNTPs and reaction components. The V4 library was generated by pooling 200 ng of each sample, quantified by Picogreen dsDNA (Invitrogen, Grand Island, NY, USA). This was followed by multiple dilution steps using pre-chilled hybridization buffer (HT1; Illumina, San Diego, CA, USA) to bring the pooled amplicons to a final concentration of 5 pM, measured by Qubit 2.0 Fluorometer (Life Technologies, Burlington, ON, Canada). Finally, 15% of PhiX control library was spiked into the amplicon pool to improve the unbalanced and biased base composition, a known characteristic of low diversity 16S rRNA libraries. Customized sequencing primers for read1 (5′-TATGGTAATTGTGTGCCAGCMGCCGCGGTAA-3′), read2 (5′-AGTCAGTCAGCCGGACTACHVGGGTWTCTAAT-3′) and index read (5′-ATTAGAWACCCBDGTAGTCCGGCTGACTGACT-3′) were synthesized and purified by polyacrylamide gel electrophoresis (Integrated DNA Technologies, Coralville, IA, USA) and added to the MiSeq Reagent Kit V2 (300-cycle; Illumina, San Diego, CA, USA). The 150 paired-end sequencing reaction was performed on a MiSeq platform (Illumina, San Diego, CA, USA) at the Gut Microbiome Laboratory (Department of Animal Science, University of Manitoba, Winnipeg, Canada). The sequencing data were deposited into the Sequence Read Archive (SRA) of NCBI (http://www.ncbi.nlm.nih.gov/sra) and can be accessed via accession number SRR4733877.

### Bioinformatic Analysis

The PANDAseq assembler^36^ was used to merge and fix the overlapping paired-end Illumina fastq files. All the sequences with low quality base calling scores, as well as those containing uncalled bases (N) in the overlapping region were discarded. The output fastq file was then analysed by downstream computational pipelines of the open source software package QIIME^37^. The default minimum quality threshold of 25 was used. Chimeric sequences were detected using the UCHIME algorithm (USEARCH 6.1) to run both *de novo* and reference based chimera detection. The number of chimeric sequences identified and consequently removed by both detection methods was 1.7% of total high quality sequences. Sequences were clustered at the 97% sequence similarity level using the Greengenes database (Version 13.5)^38^ using an open reference-based OTU picking approach with the QIIME algorithm and usearch61 method with default parameters^38, 39^. Those sequences that failed to cluster were subsampled for *de novo* OTU picking. All picked OTUs were subsequently aligned by PyNAST^40^ and a phylogenetic tree was built using FastTree method^41^ to calculate UniFrac distances^42^ within QIIME. Representative OTUs were assigned to bacterial taxonomies using RDP classifier via QIIME with a confidence threshold of 0.8^43^. Finally, open source software PICRUSt (phylogenetic investigation of communities by reconstruction of unobserved states)^44^ was used on 16S rRNA gene sequencing data to predict functional genes of the classified members of the rumen and faecal microbiota resulting from reference-based OTU picking against Greengenes database. Predicted genes were then hierarchically clustered and categorized under Kyoto Encyclopaedia of Genes and Genomes (KEGG)^45^ orthologs (KOs) and pathways (level 3; Tables S4 and S6).

### Alpha- and Beta-Diversity Analyses

The phylogenetic tree was rooted using *Methanococcus jannaschii* (L77117) as an outgroup. Subsequently an OTU table was generated by QIIME, which along with the mapping file was assembled into a Phyloseq object^46^. For within community diversity (α-diversity) calculations, the same number of sequences for each sample were randomly selected (corresponding to the sample with lowest number of sequences being 14,148 and 20,031 for rumen and faecal samples, respectively), to eliminate the bias caused by different sample sizes^47^. Alpha-diversity analysis was conducted with standard diversity metrics accessed via Phyloseq, including observed richness, Chao1, Shannon index, Simpson index, Inverse Simpson index and Phylogenetic Diversity (PD). The dataset was also subsampled to the minimum^48^ to compare microbial compositions between samples (β-diversity). Beta-diversity was measured by calculating the weighted and unweighted UniFrac distances^49^ using Phyloseq default scripts. Principal coordinate analysis (PCoA) was applied on the resulting distance matrices to generate two-dimensional plots using PRIMER v6 software^50^. Permutational multivariate analysis of variance (PERMANOVA)^51^ was used to calculate P-values and to test differences of β-diversity among treatment groups for significance. Both weighted and unweighted UniFrac distances were used to compute the test statistic and P-values^52^.

### Bacterial Community Composition and Metagenome prediction

Bacterial community composition at the phylum and genus levels, and predictive metagenome profiles were compared among treatments (early vs. late weaning) and weeks (5, 7, and 9) using the nbinomWaldTest method of DESeq2^53^ according to a randomized design with pre-weaning as covariate and containing dietary treatment, day and calf; these were considered significant at P < 0.05 (DESeq2, R package version 1.8.1, 2014). All P-values were corrected for false discovery rates using R software package (version 3.3.1, Vienna, Austria).

### Correlations between Calf Physiological Variables and Bacterial Abundance

Non-parametric Spearman rank correlation coefficient analysis implemented in PAST software^54^ was used to test the relationship between BW, average daily gain, starter intake, forage intake, water intake, metabolizable energy intake, ruminal pH, faecal starch, rumen acetate, rumen butyrate, rumen propionate, total rumen VFA and the bacterial communities in rumen fluid and faeces. Physiological data was published previously (Eckert *et al*.^11^). Data from the three corresponding ages (5, 7 and 9 weeks of age) of microbial sampling were used to produce the correlation matrix. The resulting correlation matrix was visualized in a heatmap format generated by the corrplot package of R [Corrplot: visualization of a correlation matrix; R package version 02-0. 2010 (http://CRAN)].

## Electronic supplementary material

Supplementary Information
