# Supplementary material for: Weaning age influences the severity of gastrointestinal microbiome shifts in dairy calves
Source: Sci Rep. 2017 Mar 15;7:198. doi: 10.1038/s41598-017-00223-7 (PMC5428063; doi:10.1038/s41598-017-00223-7)
Supplement: Supplementary file 1 — Supplementary Information [file 41598_2017_223_MOESM1_ESM.pdf]

Weaning age influences the severity of gastrointestinal microbiome shifts in dairy calves

S.J. Meale, S.C. Li, P. Azevedo, H. Derakhshani, T. J. DeVries, J.C. Plaizier, M.A. Steele and E. Khafipour

Supplementary Material

Supplementary Figure 1.

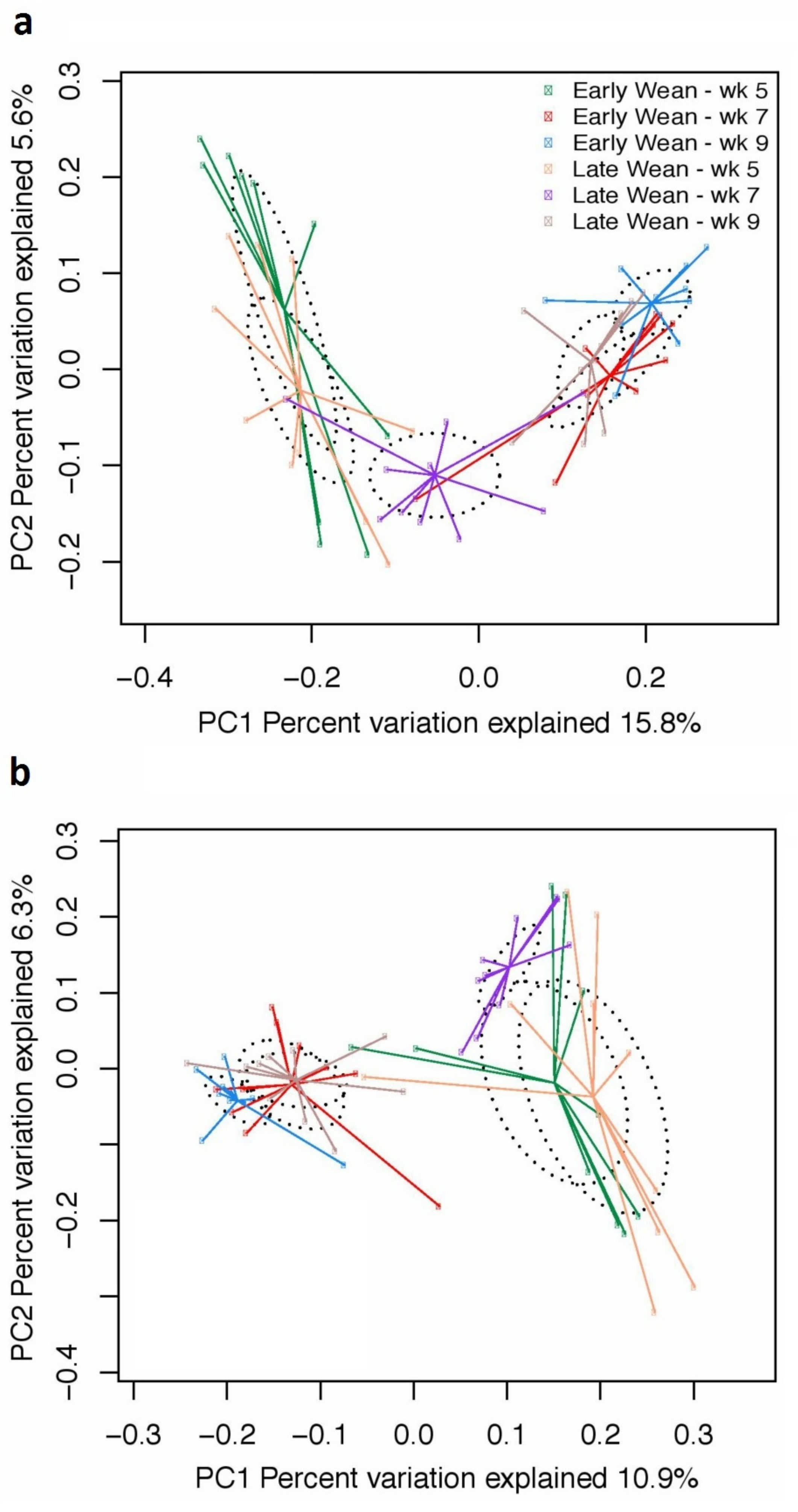

Supplementary Figure 2.

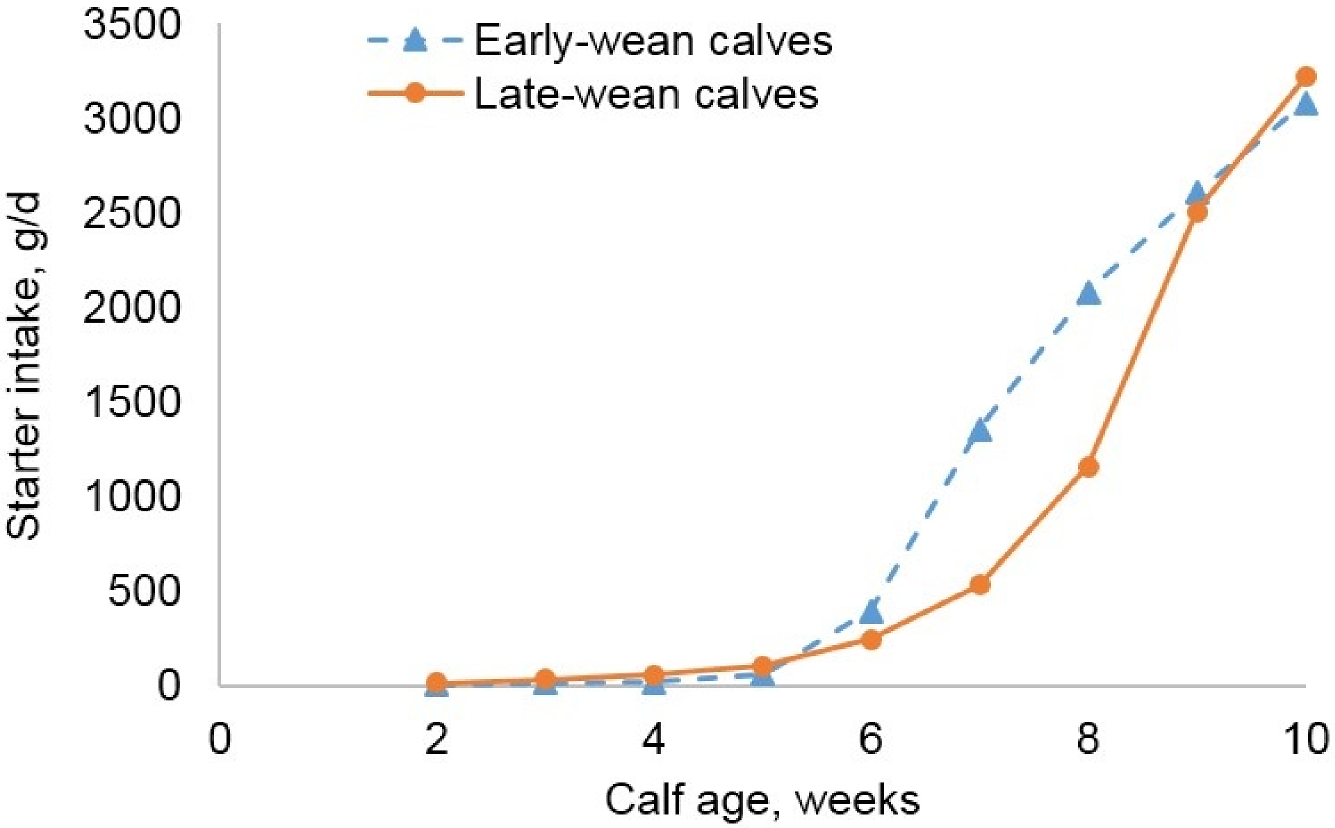

Supplementary Table 1. Rumen taxa

								Early-weaned						Late-weaned																	
		Early-weaned			Late-weaned			Wk 5 vs Wk 7		Wk 5 vs Wk 9		Wk 7 vs Wk 9		Wk 5 vs Wk 7		Wk 5 vs Wk 9		Wk 7 vs Wk 9		Early Wk 5 vs Late Wk 5		Early Wk 7 vs Late Wk 7		Early Wk 9 vs Late Wk 9		Early Wk 5 vs Late Wk 7		Early Wk 7 vs Late Wk 9		(Early Wk 7 - Wk 5) vs (Late Wk 9 - Wk 7)	
Phylum	Taxa	Wk 5	Wk7	Wk9	Wk 5	Wk7	Wk9	Log2 FC	p-value	Log2 FC	p-value	Log2 FC	p-value	Log2 FC	p-value	Log2 FC	p-value	Log2 FC	p-value	Log2 FC	p-value	Log2 FC	p-value	Log2 FC	p-value	Log2 FC	p-value	Log2 FC	p-value	Log2 FC	p-value
Acidobacteria		0.00	0.00	0.00	0.00	0.00	0.00	-0.26	1.00	-0.32	1.00	-0.06	1.00	-0.84	1.00	-0.03	1.00	0.81	1.00	0.50	1.00	1.08	1.00	0.22	1.00	1.34	1.00	0.28	1.00	-1.07	1.00
	f. Ellin6075	0.00	0.00	0.00	0.00	0.00	0.00	-0.85	1.00	-0.91	1.00	-0.06	1.00	-0.17	1.00	0.06	1.00	0.24	1.00	1.11	1.00	0.44	1.00	0.14	1.00	1.29	1.00	0.20	1.00	-1.09	1.00
	o. iii1-15	0.00	0.00	0.00	0.00	0.00	0.00	-0.17	1.00	-0.22	1.00	-0.05	1.00	-0.62	1.00	-0.30	1.00	0.32	1.00	-0.55	1.00	-0.10	1.00	-0.47	1.00	0.07	1.00	-0.42	1.00	-0.49	1.00
	f. RB40	0.00	0.00	0.00	0.00	0.00	0.00	0.27	1.00	-0.37	1.00	-0.64	1.00	-0.07	1.00	0.78	1.00	0.85	1.00	0.31	1.00	0.65	1.00	-0.84	1.00	0.38	1.00	-0.20	1.00	-0.59	1.00
Actinobacteria		3.37	6.25	5.97	2.57	6.71	4.91	0.69	0.23	0.96	0.14	0.27	1.00	1.02	0.30	1.07	0.10	0.05	0.96	0.23	0.99	-0.10	1.00	0.12	0.93	-0.79	0.81	-0.15	1.00	0.64	0.97
	o. Acidimicrobiales	0.00	0.00	0.00	0.00	0.00	0.00	0.54	1.00	0.05	1.00	-0.49	1.00	-0.17	1.00	0.06	1.00	0.24	1.00	0.20	1.00	0.92	1.00	0.18	1.00	0.38	1.00	0.68	1.00	0.30	1.00
	g. *Actinomyces*	0.15	0.00	0.00	0.06	0.00	0.00	-9.41	0.00	-8.06	0.01	1.35	1.00	-4.23	0.90	-5.60	0.26	-1.37	1.00	3.33	1.00	-1.85	1.00	0.87	1.00	7.56	0.02	-0.48	1.00	-8.04	0.54
	g. *Mobiluncus*	0.02	0.00	0.00	0.00	0.00	0.00	-1.84	1.00	-1.42	1.00	0.41	1.00	-0.25	1.00	-0.47	1.00	-0.22	1.00	1.63	1.00	0.04	1.00	0.67	1.00	1.88	1.00	0.26	1.00	-1.62	1.00
	g. *Trueperella*	0.01	0.00	0.00	0.00	0.00	0.00	-4.94	1.00	-5.00	1.00	-0.05	1.00	-0.83	1.00	-1.31	1.00	-0.48	1.00	3.21	1.00	-0.91	1.00	-0.48	1.00	4.03	1.00	-0.43	1.00	-4.46	1.00
	g. *Georgenia*	0.00	0.00	0.00	0.00	0.00	0.00	-0.17	1.00	-0.22	1.00	-0.05	1.00	0.31	1.00	0.03	1.00	-0.29	1.00	-0.06	1.00	-0.54	1.00	-0.30	1.00	-0.37	1.00	-0.26	1.00	0.11	1.00
	g. *Brevibacterium*	0.00	0.00	0.00	0.00	0.00	0.00	-1.73	1.00	-0.18	1.00	1.55	1.00	-1.47	1.00	-1.37	1.00	0.10	1.00	0.27	1.00	0.00	1.00	1.46	1.00	1.73	1.00	-0.09	1.00	-1.83	1.00
	g. *Demequina*	0.00	0.00	0.00	0.00	0.00	0.00	-0.62	1.00	-0.73	1.00	-0.11	1.00	-0.17	1.00	0.06	1.00	0.24	1.00	0.95	1.00	0.50	1.00	0.15	1.00	1.12	1.00	0.26	1.00	-0.86	1.00
	f. Cellulomonadaceae	0.00	0.00	0.00	0.00	0.00	0.00	0.19	1.00	-0.38	1.00	-0.56	1.00	-0.17	1.00	0.06	1.00	0.24	1.00	0.48	1.00	0.84	1.00	0.04	1.00	0.65	1.00	0.60	1.00	-0.05	1.00
	g. *Corynebacterium*	0.43	0.01	0.00	0.17	0.05	0.01	-5.64	0.00	-6.72	0.00	-1.08	1.00	-2.18	0.19	-3.87	0.00	-1.70	1.00	0.66	1.00	-2.80	0.09	-2.19	1.00	2.84	0.00	-1.11	1.00	-3.94	0.08
	g. *Brachybacterium*	0.00	0.00	0.00	0.00	0.00	0.00	-3.21	1.00	-3.26	1.00	-0.05	1.00	-0.93	1.00	-0.30	1.00	0.63	1.00	1.99	1.00	-0.29	1.00	-0.97	1.00	2.92	1.00	-0.92	1.00	-3.84	1.00
	g. *Dietzia*	0.01	0.00	0.00	0.02	0.00	0.00	-3.41	0.74	-2.98	1.00	0.44	1.00	-1.70	1.00	-2.93	1.00	-1.23	1.00	1.29	1.00	-0.42	1.00	1.24	1.00	2.99	1.00	0.80	1.00	-2.19	1.00
	f. Dietziaceae	0.00	0.00	0.00	0.00	0.00	0.00	-0.73	1.00	-0.83	1.00	-0.10	1.00	-0.84	1.00	-0.54	1.00	0.30	1.00	-0.07	1.00	0.03	1.00	-0.36	1.00	0.77	1.00	-0.27	1.00	-1.04	1.00
	f. Geodermatophilaceae	0.00	0.00	0.00	0.00	0.00	0.00	-0.08	1.00	0.47	1.00	0.55	1.00	-0.24	1.00	0.52	1.00	0.76	1.00	0.07	1.00	0.23	1.00	0.02	1.00	0.31	1.00	-0.53	1.00	-0.84	1.00
	g. *Gordonia*	0.00	0.00	0.00	0.00	0.00	0.00	-2.66	1.00	-3.12	1.00	-0.47	1.00	-0.79	1.00	-0.04	1.00	0.75	1.00	2.22	1.00	0.35	1.00	-0.86	1.00	3.01	1.00	-0.40	1.00	-3.41	1.00
	g. *Frigoribacterium*	0.00	0.00	0.00	0.00	0.00	0.00	-0.78	1.00	-0.90	1.00	-0.12	1.00	1.08	1.00	-0.02	1.00	-1.10	1.00	0.58	1.00	-1.28	1.00	-0.30	1.00	-0.50	1.00	-0.18	1.00	0.32	1.00
	g. *Leucobacter*	0.02	0.00	0.00	0.01	0.00	0.00	-6.30	1.00	-5.57	1.00	0.72	1.00	-2.28	1.00	-4.11	1.00	-1.83	1.00	1.80	1.00	-2.22	1.00	0.33	1.00	4.08	1.00	-0.39	1.00	-4.47	1.00
	f. Microbacteriaceae	0.02	0.01	0.00	0.01	0.00	0.00	-1.37	1.00	-2.26	0.28	-0.89	1.00	-0.02	1.00	-0.26	1.00	-0.24	1.00	2.79	1.00	1.45	1.00	0.80	1.00	2.81	0.34	1.69	1.00	-1.13	1.00
	g. *Rathayibacter*	0.00	0.00	0.00	0.00	0.00	0.00	-0.35	1.00	0.17	1.00	0.52	1.00	-0.17	1.00	0.06	1.00	0.24	1.00	0.62	1.00	0.44	1.00	0.72	1.00	0.79	1.00	0.20	1.00	-0.59	1.00
	g. *Salinibacterium*	0.00	0.00	0.00	0.00	0.00	0.00	-0.62	1.00	-0.73	1.00	-0.11	1.00	-0.17	1.00	0.06	1.00	0.24	1.00	0.95	1.00	0.50	1.00	0.15	1.00	1.12	1.00	0.26	1.00	-0.86	1.00
	g. *Arthrobacter*	0.01	0.00	0.00	0.00	0.00	0.00	-4.20	1.00	-4.06	1.00	0.13	1.00	-2.22	1.00	-2.49	1.00	-0.27	1.00	1.07	1.00	-0.90	1.00	-0.50	1.00	3.29	1.00	-0.63	1.00	-3.92	1.00
	g. *Microbispora*	0.00	0.00	0.00	0.00	0.00	0.00	-0.17	1.00	-0.22	1.00	-0.05	1.00	-0.67	1.00	-0.61	1.00	0.06	1.00	-0.54	1.00	-0.04	1.00	-0.14	1.00	0.13	1.00	-0.10	1.00	-0.23	1.00
	f. Micrococcaceae	0.00	0.00	0.00	0.00	0.00	0.00	-0.78	1.00	-0.84	1.00	-0.06	1.00	-0.17	1.00	0.06	1.00	0.24	1.00	0.82	1.00	0.21	1.00	-0.08	1.00	0.99	1.00	-0.03	1.00	-1.02	1.00
	g. *Rothia*	0.00	0.00	0.00	0.00	0.00	0.00	-0.82	1.00	-0.88	1.00	-0.06	1.00	-0.17	1.00	0.06	1.00	0.24	1.00	0.94	1.00	0.29	1.00	0.00	1.00	1.11	1.00	0.05	1.00	-1.06	1.00
	g. *Rhodococcus*	0.00	0.00	0.00	0.00	0.00	0.00	-1.34	1.00	-1.39	1.00	-0.04	1.00	-0.17	1.00	0.06	1.00	0.24	1.00	1.71	1.00	0.54	1.00	0.26	1.00	1.89	1.00	0.30	1.00	-1.58	1.00
	f. Nocardiopsaceae	0.00	0.00	0.00	0.00	0.00	0.00	-0.72	1.00	-0.82	1.00	-0.10	1.00	-0.17	1.00	0.06	1.00	0.24	1.00	1.01	1.00	0.46	1.00	0.12	1.00	1.18	1.00	0.23	1.00	-0.96	1.00
	g. *Pseudonocardia*	0.00	0.00	0.00	0.00	0.00	0.00	-0.87	1.00	-0.79	1.00	0.07	1.00	-0.17	1.00	0.06	1.00	0.24	1.00	1.00	1.00	0.31	1.00	0.15	1.00	1.18	1.00	0.07	1.00	-1.11	1.00
	g. *Streptomyces*	0.00	0.00	0.00	0.00	0.00	0.00	-0.79	1.00	-0.74	1.00	0.05	1.00	-0.82	1.00	-0.12	1.00	0.71	1.00	-0.28	1.00	-0.24	1.00	-0.90	1.00	0.54	1.00	-0.95	1.00	-1.49	1.00
	g. *Yaniella*	0.00	0.00	0.00	0.00	0.00	0.00	-0.17	1.00	-0.22	1.00	-0.05	1.00	-0.83	1.00	-0.53	1.00	0.30	1.00	-0.60	1.00	0.05	1.00	-0.29	1.00	0.23	1.00	-0.25	1.00	-0.48	1.00
	g. *Bifidobacterium*	0.54	0.04	0.15	0.32	0.15	0.32	-5.44	0.00	-3.24	0.12	2.20	1.00	-3.13	0.55	-2.00	1.00	1.14	1.00	-0.50	1.00	-2.81	1.00	-1.74	1.00	2.64	0.47	-3.94	1.00	-6.58	0.12
	f. Bifidobacteriaceae	0.00	0.00	0.00	0.00	0.00	0.00	0.26	1.00	-0.35	1.00	-0.61	1.00	-0.17	1.00	0.06	1.00	0.24	1.00	0.62	1.00	1.05	1.00	0.20	1.00	0.80	1.00	0.81	1.00	0.02	1.00
	g. *Adlercreutzia*	0.00	0.01	0.00	0.00	0.00	0.00	4.40	0.07	1.72	1.00	-2.67	1.00	2.52	1.00	3.19	0.91	0.67	1.00	2.10	1.00	3.98	1.00	0.64	1.00	-0.42	1.00	3.31	1.00	3.73	1.00
	g. *Atopobium*	0.00	0.00	0.00	0.00	0.00	0.00	-0.17	1.00	-0.22	1.00	-0.05	1.00	0.00	1.00	-0.50	1.00	-0.50	1.00	-0.87	1.00	-1.04	1.00	-0.59	1.00	-0.87	1.00	-0.54	1.00	0.33	1.00
	g. *Collinsella*	0.05	0.00	0.00	0.01	0.00	0.00	-7.38	0.11	-6.13	0.18	1.24	1.00	-4.05	1.00	-2.66	1.00	1.39	1.00	2.92	1.00	-0.41	1.00	-0.56	1.00	6.97	0.20	-1.80	1.00	-8.77	0.83
	g. *Eggerthella*	0.00	0.00	0.00	0.00	0.00	0.00	-1.32	1.00	-1.31	1.00	0.00	1.00	-0.17	1.00	0.06	1.00	0.24	1.00	1.68	1.00	0.54	1.00	0.31	1.00	1.86	1.00	0.30	1.00	-1.55	1.00
	f. Coriobacteriaceae	2.09	6.17	5.80	1.95	6.49	4.57	2.15	0.00	2.33	0.00	0.18	1.00	1.78	0.19	1.57	0.15	-0.21	1.00	-0.41	1.00	-0.04	1.00	0.35	1.00	-2.19	0.01	0.17	1.00	2.36	0.17
	g. *Slackia*	0.00	0.00	0.00	0.00	0.00	0.00	0.20	1.00	-0.38	1.00	-0.58	1.00	-0.23	1.00	0.55	1.00	0.78	1.00	0.43	1.00	0.86	1.00	-0.50	1.00	0.66	1.00	0.07	1.00	-0.59	1.00
	f. Patulibacteraceae	0.00	0.00	0.00	0.00	0.00	0.00	-0.62	1.00	-0.61	1.00	0.01	1.00	-0.17	1.00	0.06	1.00	0.24	1.00	1.01	1.00	0.56	1.00	0.34	1.00	1.19	1.00	0.32	1.00	-0.86	1.00
Bacteroidetes		47.00	27.44	17.22	60.54	42.79	21.85	-0.81	0.04	-1.38	0.00	-0.58	1.00	-0.97	0.00	-1.54	0.00	-0.58	0.58	-0.61	0.99	-0.45	0.72	-0.45	0.83	0.35	0.83	0.12	1.00	-0.23	0.97
	f. Chitinophagaceae	0.00	0.00	0.00	0.00	0.00	0.00	-0.78	1.00	-0.90	1.00	-0.12	1.00	-0.82	1.00	-0.52	1.00	0.30	1.00	0.20	1.00	0.23	1.00	-0.19	1.00	1.02	1.00	-0.07	1.00	-1.08	1.00

	o. [Saprospirales]	0.00	0.00	0.00	0.00	0.00	0.00	-0.17	1.00	-0.22	1.00	-0.05	1.00	-0.03	1.00	-0.42	1.00	-0.39	1.00	-0.67	1.00	-0.82	1.00	-0.47	1.00	-0.64	1.00	-0.42	1.00	0.22	1.00
	f. Saprospiraceae	0.00	0.00	0.00	0.00	0.00	0.00	-0.17	1.00	-0.22	1.00	-0.05	1.00	-0.23	1.00	0.57	1.00	0.80	1.00	-0.04	1.00	0.02	1.00	-0.83	1.00	0.19	1.00	-0.78	1.00	-0.97	1.00
	f. [Barnesiellaceae]	0.19	0.00	0.00	0.03	0.00	0.00	-9.55	0.47	-8.87	0.47	0.68	1.00	-5.19	1.00	-4.78	1.00	0.41	1.00	4.77	1.00	0.41	1.00	0.68	1.00	9.96	0.50	0.00	1.00	-9.97	1.00
	g. *Butyricimonas*	2.22	0.02	0.01	1.38	0.06	0.01	-8.74	0.00	-9.21	0.00	-0.47	1.00	-4.55	0.00	-5.88	0.00	-1.34	1.00	1.51	1.00	-2.68	0.23	-1.82	1.00	6.06	0.00	-1.34	1.00	-7.40	0.00
	g. *Odoribacter*	0.11	0.00	0.00	0.10	0.01	0.00	-7.78	0.00	-9.63	0.00	-1.85	1.00	-2.76	0.19	-7.48	0.00	-4.72	0.40	0.89	1.00	-4.12	0.17	-1.26	1.00	3.65	0.00	0.60	1.00	-3.06	1.00
	g. Prevotella 1	21.90	23.17	14.74	39.70	29.85	18.79	0.21	1.00	-0.46	1.00	-0.66	1.00	-0.29	1.00	-0.97	0.28	-0.68	1.00	-0.92	1.00	-0.42	1.00	-0.40	1.00	-0.62	0.78	0.26	1.00	0.89	1.00
	g. *[Prevotella] 2*	4.50	0.77	0.05	3.76	1.75	0.15	-4.43	0.00	-6.94	0.00	-2.51	0.53	-1.26	1.00	-5.46	0.00	-4.20	0.00	0.73	1.00	-2.44	0.09	-0.75	1.00	1.99	0.09	1.76	1.00	-0.23	1.00
	g. *CF231*	0.13	0.01	0.00	1.92	0.27	0.00	-7.27	0.04	-5.23	0.16	2.04	1.00	-4.24	1.00	-10.38	0.01	-6.14	0.98	3.26	1.00	0.23	1.00	8.41	0.18	7.50	0.02	6.37	1.00	-1.13	1.00
	f. [Paraprevotellaceae]	0.51	0.64	0.58	1.40	0.77	0.42	-0.36	1.00	-0.33	1.00	0.03	1.00	-0.50	1.00	-1.22	1.00	-0.71	1.00	-0.88	1.00	-0.73	1.00	0.00	1.00	-0.37	1.00	-0.02	1.00	0.35	1.00
	g. *YRC22*	0.93	0.13	0.13	1.19	3.39	0.52	-2.54	0.02	-2.72	0.01	-0.18	1.00	0.35	1.00	-1.43	0.64	-1.78	1.00	-1.45	1.00	-4.33	0.00	-2.74	0.18	-1.79	0.25	-2.56	1.00	-0.77	1.00
	g. *5-7N15*	0.00	0.00	0.00	0.00	0.00	0.00	-0.17	1.00	-0.22	1.00	-0.05	1.00	-0.27	1.00	1.27	1.00	1.54	1.00	-0.11	1.00	-0.01	1.00	-1.59	1.00	0.17	1.00	-1.55	1.00	-1.71	1.00
	g. *Bacteroides*	7.28	0.07	0.06	3.61	0.52	0.21	-7.75	0.00	-7.30	0.00	0.45	1.00	-3.70	0.00	-4.73	0.00	-1.03	1.00	1.87	1.00	-2.18	0.25	-0.70	1.00	5.57	0.00	-1.15	1.00	-6.72	0.00
	f. Bacteroidaceae	0.00	0.00	0.00	0.00	0.00	0.00	-0.71	1.00	-0.81	1.00	-0.10	1.00	-0.17	1.00	0.06	1.00	0.24	1.00	0.94	1.00	0.41	1.00	0.07	1.00	1.12	1.00	0.17	1.00	-0.95	1.00
	f. BS11	0.00	0.00	0.00	0.00	0.00	0.00	-0.17	1.00	-0.22	1.00	-0.05	1.00	0.26	1.00	0.01	1.00	-0.25	1.00	-0.21	1.00	-0.65	1.00	-0.45	1.00	-0.48	1.00	-0.40	1.00	0.08	1.00
	f. Marinilabiaceae	0.00	0.00	0.00	0.00	0.00	0.00	0.61	1.00	0.08	1.00	-0.53	1.00	-0.17	1.00	0.06	1.00	0.24	1.00	0.21	1.00	1.00	1.00	0.23	1.00	0.39	1.00	0.76	1.00	0.37	1.00
	o. Bacteroidales	4.91	0.89	0.97	4.99	4.52	0.90	-1.86	0.02	-2.02	0.01	-0.17	1.00	0.05	1.00	-2.04	0.01	-2.09	0.10	-0.01	1.00	-1.91	0.09	0.01	1.00	-0.06	1.00	0.18	1.00	0.24	1.00
	f. Porphyromonadaceae	0.01	0.00	0.00	0.01	0.00	0.00	-3.02	1.00	-2.48	1.00	0.54	1.00	-0.69	1.00	-0.40	1.00	0.29	1.00	2.51	1.00	0.18	1.00	0.43	1.00	3.20	1.00	-0.11	1.00	-3.31	1.00
	g. *Paludibacter*	0.16	0.00	0.00	0.03	0.00	0.00	-8.79	0.61	-8.78	0.48	0.01	1.00	-1.66	1.00	-0.48	1.00	1.17	1.00	6.29	1.00	-0.84	1.00	-2.01	1.00	7.95	0.78	-2.01	1.00	-9.96	1.00
	g. *Parabacteroides*	0.93	0.01	0.01	0.52	0.03	0.02	-8.54	0.00	-9.10	0.00	-0.55	1.00	-4.90	0.01	-4.75	0.00	0.14	1.00	1.87	1.00	-1.78	1.00	-2.47	1.00	6.77	0.00	-1.92	1.00	-8.69	0.00
	g. *Porphyromonas*	0.33	0.00	0.00	0.07	0.00	0.00	-8.64	0.03	-8.90	0.02	-0.25	1.00	-1.88	1.00	-1.22	1.00	0.66	1.00	8.85	1.00	2.08	1.00	1.17	1.00	10.73	0.01	1.42	1.00	-9.30	0.64
	g. *Tannerella*	0.00	0.00	0.00	0.00	0.00	0.00	-0.86	1.00	-0.89	1.00	-0.03	1.00	-0.17	1.00	0.06	1.00	0.24	1.00	0.93	1.00	0.25	1.00	-0.03	1.00	1.10	1.00	0.01	1.00	-1.10	1.00
	f. Prevotellaceae	0.00	0.00	0.00	0.00	0.00	0.00	-1.89	1.00	-2.00	1.00	-0.11	1.00	-0.96	1.00	-1.22	1.00	-0.26	1.00	-0.12	1.00	-1.05	1.00	-0.90	1.00	0.84	1.00	-0.79	1.00	-1.63	1.00
	f. RF16	0.01	0.00	0.00	0.00	0.00	0.00	-0.46	1.00	-1.26	1.00	-0.80	1.00	-0.96	1.00	0.24	1.00	1.20	1.00	0.17	1.00	0.67	1.00	-1.33	1.00	1.13	1.00	-0.53	1.00	-1.66	1.00
	f. Rikenellaceae	0.63	0.00	0.00	0.31	0.02	0.01	-8.46	0.00	-9.77	0.00	-1.31	1.00	-5.14	0.01	-5.48	0.00	-0.34	1.00	2.09	1.00	-1.22	1.00	-2.19	1.00	7.24	0.00	-0.88	1.00	-8.12	0.00
	f. S24-7	2.15	1.71	0.65	1.37	1.58	0.80	0.62	1.00	-0.06	1.00	-0.67	1.00	-0.37	1.00	0.61	1.00	0.98	1.00	-0.37	1.00	0.61	1.00	-1.04	1.00	-0.01	1.00	-0.37	1.00	-0.36	1.00
	g. *Dyadobacter*	0.00	0.00	0.00	0.00	0.00	0.00	-0.82	1.00	-0.88	1.00	-0.06	1.00	-0.17	1.00	0.06	1.00	0.24	1.00	0.94	1.00	0.29	1.00	0.00	1.00	1.11	1.00	0.05	1.00	-1.06	1.00
	f. Flammeovirgaceae	0.00	0.00	0.00	0.00	0.00	0.00	0.20	1.00	-0.38	1.00	-0.58	1.00	-0.17	1.00	0.06	1.00	0.24	1.00	0.68	1.00	1.05	1.00	0.23	1.00	0.85	1.00	0.81	1.00	-0.04	1.00
	g. *Chryseobacterium*	0.00	0.00	0.00	0.00	0.00	0.00	-0.42	1.00	-24.33	1.00	-23.91	0.05	-3.09	1.00	5.96	1.00	9.05	1.00	-2.21	1.00	0.46	1.00	-32.50	1.00	0.88	1.00	-8.59	1.00	-9.48	1.00
	f. [Weeksellaceae]	0.01	0.00	0.00	0.01	0.00	0.00	-4.22	0.09	-5.86	1.00	-1.64	1.00	-3.60	1.00	-5.10	0.15	-1.50	1.00	0.67	1.00	0.06	1.00	-0.09	1.00	4.27	1.00	1.56	1.00	-2.72	1.00
	g. *Wautersiella*	0.01	0.01	0.00	0.03	0.00	0.00	-2.49	1.00	-3.64	0.78	-1.15	1.00	-1.27	1.00	-3.94	1.00	-2.66	1.00	0.96	1.00	-0.25	1.00	1.26	1.00	2.24	1.00	2.41	1.00	0.17	1.00
	g. *Weeksella*	0.01	0.00	0.00	0.01	0.00	0.00	-4.67	1.00	-4.76	1.00	-0.08	1.00	-1.10	1.00	-2.36	1.00	-1.26	1.00	0.87	1.00	-2.70	1.00	-1.52	1.00	1.97	1.00	-1.44	1.00	-3.42	1.00
	g. *Fluviicola*	0.00	0.00	0.00	0.00	0.00	0.00	-0.17	1.00	-0.22	1.00	-0.05	1.00	-0.73	1.00	-0.49	1.00	0.24	1.00	-0.60	1.00	-0.05	1.00	-0.34	1.00	0.12	1.00	-0.29	1.00	-0.41	1.00
	g. *Myroides*	0.00	0.00	0.00	0.00	0.00	0.00	-0.17	1.00	-0.22	1.00	-0.05	1.00	-0.83	1.00	0.04	1.00	0.87	1.00	-0.84	1.00	-0.18	1.00	-1.10	1.00	0.00	1.00	-1.05	1.00	-1.05	1.00
	f. Flavobacteriaceae	0.01	0.00	0.00	0.01	0.00	0.00	-4.53	0.49	-4.26	1.00	0.27	1.00	-3.18	1.00	-1.96	1.00	1.22	1.00	1.68	1.00	0.33	1.00	-0.62	1.00	4.87	1.00	-0.88	1.00	-5.75	1.00
	f. Sphingobacteriaceae	0.02	0.00	0.00	0.02	0.00	0.00	-4.94	0.07	-5.69	0.03	-0.75	1.00	-3.03	0.90	-4.27	0.26	-1.24	1.00	0.08	1.00	-1.84	1.00	-1.34	1.00	3.11	0.50	-0.59	1.00	-3.70	1.00
	g. *Pedobacter*	0.00	0.00	0.00	0.00	0.00	0.00	-0.62	1.00	-0.73	1.00	-0.11	1.00	-0.67	1.00	-0.61	1.00	0.06	1.00	0.21	1.00	0.26	1.00	0.09	1.00	0.88	1.00	0.20	1.00	-0.68	1.00
	g. Sphingobacterium	0.04	0.00	0.00	0.04	0.00	0.00	-7.52	0.00	-7.30	0.00	0.21	1.00	-3.71	0.76	-6.00	0.11	-2.28	1.00	2.58	1.00	-1.22	1.00	1.28	1.00	6.30	0.01	1.06	1.00	-5.23	1.00
BRC1	c. PRR-11	0.00	0.00	0.00	0.00	0.00	0.00	-0.57	1.00	-0.59	1.00	-0.02	1.00	-0.58	1.00	-0.06	1.00	0.52	1.00	0.54	1.00	0.55	1.00	0.00	1.00	1.12	1.00	0.03	1.00	-1.09	1.00
Chlorobi	c. OPB56	0.00	0.00	0.00	0.00	0.00	0.00	-0.05	1.00	0.71	1.00	0.76	1.00	-0.58	1.00	-0.06	1.00	0.52	1.00	-0.13	1.00	0.39	1.00	0.63	1.00	0.44	1.00	-0.13	1.00	-0.57	1.00
Chloroflexi		0.00	0.00	0.00	0.00	0.00	0.00	-0.55	1.00	0.92	1.00	1.47	1.00	-0.58	1.00	-0.06	1.00	0.52	1.00	0.68	1.00	0.72	1.00	1.67	1.00	1.26	1.00	0.20	1.00	-1.06	1.00
	o. CFB-26	0.00	0.00	0.00	0.00	0.00	0.00	-0.82	1.00	-0.88	1.00	-0.06	1.00	-0.17	1.00	0.06	1.00	0.24	1.00	0.94	1.00	0.29	1.00	0.00	1.00	1.11	1.00	0.05	1.00	-1.06	1.00
	f. A4b	0.00	0.00	0.00	0.00	0.00	0.00	0.01	1.00	0.47	1.00	0.46	1.00	-0.17	1.00	0.06	1.00	0.24	1.00	0.20	1.00	0.39	1.00	0.61	1.00	0.38	1.00	0.15	1.00	-0.23	1.00
	g. *Chloronema*	0.00	0.00	0.00	0.00	0.00	0.00	-0.33	1.00	0.30	1.00	0.63	1.00	-0.17	1.00	0.06	1.00	0.24	1.00	0.49	1.00	0.34	1.00	0.73	1.00	0.67	1.00	0.10	1.00	-0.57	1.00
Cyanobacteria		0.83	0.15	0.31	1.04	1.06	0.49	-1.65	0.04	-0.42	1.00	1.23	1.00	-0.23	0.98	-0.35	0.99	-0.12	0.96	-0.74	0.99	-2.17	0.01	-0.81	0.83	-0.51	0.83	-2.04	0.11	-1.53	0.97
	o. YS2	0.81	0.09	0.23	1.02	1.01	0.39	-3.44	0.02	-0.95	1.00	2.49	1.00	-0.18	1.00	-0.97	1.00	-0.79	1.00	-0.04	1.00	-3.30	0.14	-0.01	1.00	0.14	1.00	-2.50	1.00	-2.65	1.00
	o. Streptophyta	0.02	0.06	0.08	0.02	0.05	0.10	2.32	0.00	2.74	0.00	0.42	1.00	1.46	0.32	2.84	0.00	1.38	0.82	-0.20	1.00	0.67	1.00	-0.29	1.00	-1.66	0.06	-0.71	1.00	0.95	1.00
	c. ML635J-21	0.00	0.00	0.00	0.00	0.00	0.00	-0.26	1.00	0.17	1.00	0.43	1.00	-0.17	1.00	0.06	1.00	0.24	1.00	0.62	1.00	0.54	1.00	0.72	1.00	0.79	1.00	0.30	1.00	-0.50	1.00
Elusimicrobia		0.57	0.00	0.00	0.29	0.03	0.00	-7.95	0.00	-8.91	0.00	-0.97	1.00	-4.15	0.02	-8.97	0.00	-4.82	0.03	0.36	0.99	-3.44	0.06	0.41	0.93	4.51	0.00	1.38	1.00	-3.13	0.97

	g. *Elusimicrobium*	0.10	0.00	0.00	0.05	0.00	0.00	-23.56	1.00	-23.14	1.00	0.41	1.00	-21.48	1.00	-21.24	1.00	0.24	1.00	2.07	1.00	-0.01	1.00	0.17	1.00	23.55	0.00	-0.25	1.00	-23.79	0.17
	f. Elusimicrobiaceae	0.47	0.00	0.00	0.24	0.03	0.00	-7.62	0.00	-8.61	0.00	-0.99	1.00	-3.28	0.45	-8.45	0.00	-5.18	0.40	0.72	1.00	-3.62	0.54	0.56	1.00	4.00	0.04	1.56	1.00	-2.44	1.00
Fibrobacteres	g. Fibrobacter	0.10	0.54	0.00	0.40	0.88	0.01	-5.43	0.00	-7.21	0.00	-1.77	1.00	0.23	1.00	-5.94	0.01	-6.17	0.02	-0.69	1.00	-6.36	0.00	-1.96	1.00	-0.93	1.00	-0.19	1.00	0.74	1.00
Firmicutes		39.32	58.59	69.59	29.12	40.91	62.59	0.61	0.14	1.01	0.02	0.40	1.00	0.16	0.98	1.29	0.00	1.13	0.01	0.39	0.99	0.85	0.06	0.12	0.93	0.23	0.83	-0.28	1.00	-0.51	0.97
	g. Bacillus	0.00	0.00	0.00	0.00	0.00	0.00	-1.72	1.00	-0.40	1.00	1.32	1.00	-0.20	1.00	-0.38	1.00	-0.19	1.00	0.10	1.00	-1.42	1.00	0.09	1.00	0.30	1.00	-1.23	1.00	-1.53	1.00
	o. Bacillales	0.01	0.00	0.00	0.00	0.00	0.00	-4.29	1.00	-4.31	1.00	-0.02	1.00	0.15	1.00	-0.89	1.00	-1.04	1.00	1.86	1.00	-2.58	1.00	-1.56	1.00	1.71	1.00	-1.54	1.00	-3.25	1.00
	g. *Paenibacillus*	0.00	0.00	0.00	0.00	0.00	0.00	-0.17	1.00	-0.22	1.00	-0.05	1.00	-0.24	1.00	1.25	1.00	1.49	1.00	-0.12	1.00	-0.05	1.00	-1.59	1.00	0.12	1.00	-1.55	1.00	-1.67	1.00
	f. Planococcaceae	0.00	0.00	0.00	0.00	0.00	0.00	-0.46	1.00	-0.46	1.00	0.00	1.00	-0.57	1.00	1.04	1.00	1.61	1.00	-0.17	1.00	-0.06	1.00	-1.67	1.00	0.40	1.00	-1.67	1.00	-2.07	1.00
	g. *Planomicrobium*	0.00	0.00	0.00	0.00	0.00	0.00	-0.17	1.00	-0.22	1.00	-0.05	1.00	-0.06	1.00	0.70	1.00	0.77	1.00	0.04	1.00	-0.08	1.00	-0.89	1.00	0.10	1.00	-0.84	1.00	-0.94	1.00
	g. *Rummeliibacillus*	0.00	0.00	0.00	0.00	0.00	0.00	-0.17	1.00	-0.22	1.00	-0.05	1.00	-0.21	1.00	0.61	1.00	0.82	1.00	0.01	1.00	0.04	1.00	-0.83	1.00	0.22	1.00	-0.78	1.00	-1.00	1.00
	g. *Sporosarcina*	0.00	0.00	0.00	0.00	0.00	0.00	-0.17	1.00	-0.22	1.00	-0.05	1.00	-0.82	1.00	-0.52	1.00	0.30	1.00	-0.54	1.00	0.11	1.00	-0.24	1.00	0.28	1.00	-0.19	1.00	-0.47	1.00
	g. *Jeotgalicoccus*	0.00	0.00	0.00	0.00	0.00	0.00	-1.56	1.00	-1.40	1.00	0.15	1.00	-0.17	1.00	0.06	1.00	0.24	1.00	1.90	1.00	0.52	1.00	0.43	1.00	2.08	1.00	0.28	1.00	-1.80	1.00
	g. *Staphylococcus*	0.01	0.00	0.00	0.01	0.00	0.00	-1.44	1.00	-0.77	1.00	0.67	1.00	-1.38	1.00	-1.66	1.00	-0.27	1.00	-0.84	1.00	-0.89	1.00	0.05	1.00	0.54	1.00	-0.62	1.00	-1.16	1.00
	g. *Aerococcus*	0.00	0.00	0.00	0.00	0.00	0.00	-0.87	1.00	-0.79	1.00	0.07	1.00	-0.17	1.00	0.06	1.00	0.24	1.00	1.00	1.00	0.31	1.00	0.15	1.00	1.18	1.00	0.07	1.00	-1.11	1.00
	g. Facklamia	0.01	0.00	0.00	0.00	0.00	0.00	-2.58	1.00	-3.26	1.00	-0.67	1.00	-3.31	1.00	-3.07	1.00	0.24	1.00	0.78	1.00	1.50	1.00	0.59	1.00	4.09	1.00	1.27	1.00	-2.82	1.00
	f. Aerococcaceae	0.00	0.00	0.00	0.00	0.00	0.00	0.54	1.00	0.05	1.00	-0.49	1.00	-0.52	1.00	0.10	1.00	0.62	1.00	-0.39	1.00	0.68	1.00	-0.43	1.00	0.14	1.00	0.06	1.00	-0.08	1.00
	g. *Carnobacterium*	0.00	0.00	0.00	0.00	0.00	0.00	-0.17	1.00	-0.22	1.00	-0.05	1.00	-0.67	1.00	-0.61	1.00	0.06	1.00	-0.54	1.00	-0.04	1.00	-0.14	1.00	0.13	1.00	-0.10	1.00	-0.23	1.00
	g. *Trichococcus*	0.00	0.00	0.00	0.00	0.00	0.00	-0.54	1.00	-0.58	1.00	-0.04	1.00	-0.18	1.00	-0.75	1.00	-0.57	1.00	0.67	1.00	0.31	1.00	0.84	1.00	0.85	1.00	0.89	1.00	0.03	1.00
	g. *Enterococcus*	0.10	0.00	0.00	0.01	0.00	0.00	-6.91	0.75	-5.13	1.00	1.78	1.00	-2.76	1.00	-2.08	1.00	0.68	1.00	3.99	1.00	-0.16	1.00	0.93	1.00	6.75	0.84	-0.85	1.00	-7.60	1.00
	f. Enterococcaceae	0.00	0.00	0.00	0.00	0.00	0.00	-1.42	1.00	-1.45	1.00	-0.03	1.00	-0.17	1.00	0.06	1.00	0.24	1.00	1.47	1.00	0.22	1.00	-0.05	1.00	1.64	1.00	-0.02	1.00	-1.66	1.00
	g. *Lactobacillus*	0.00	0.00	0.00	0.00	0.00	0.00	-6.21	1.00	-0.18	1.00	6.03	1.00	-2.35	1.00	-3.22	1.00	-0.87	1.00	4.63	1.00	0.76	1.00	7.67	1.00	6.97	1.00	1.63	1.00	-5.34	1.00
	f. Lactobacillaceae	0.00	0.00	0.00	0.00	0.00	0.00	0.19	1.00	-0.38	1.00	-0.56	1.00	-0.17	1.00	0.06	1.00	0.24	1.00	0.48	1.00	0.84	1.00	0.04	1.00	0.65	1.00	0.60	1.00	-0.05	1.00
	g. *Leuconostoc*	0.00	0.00	0.00	0.00	0.00	0.00	-1.12	1.00	-1.18	1.00	-0.05	1.00	-0.23	1.00	0.57	1.00	0.81	1.00	1.48	1.00	0.59	1.00	-0.27	1.00	1.71	1.00	-0.22	1.00	-1.93	1.00
	f. Leuconostocaceae	0.00	0.00	0.00	0.00	0.00	0.00	0.22	1.00	-1.19	1.00	-1.41	1.00	-1.49	1.00	-1.19	1.00	0.31	1.00	-0.08	1.00	1.64	1.00	-0.08	1.00	1.41	1.00	1.33	1.00	-0.08	1.00
	o. Lactobacillales	0.01	0.00	0.00	0.00	0.00	0.00	-1.97	1.00	-2.21	1.00	-0.25	1.00	0.27	1.00	-5.40	1.00	-5.67	1.00	-3.45	1.00	-5.69	1.00	-0.26	1.00	-3.72	1.00	-0.01	1.00	3.70	1.00
	g. *Streptococcus*	7.82	0.01	0.02	0.44	0.03	0.04	-9.53	0.00	-6.85	0.00	2.68	1.00	-3.05	0.29	-1.55	1.00	1.50	1.00	4.96	0.04	-1.52	1.00	-0.34	1.00	8.01	0.00	-3.02	1.00	-11.03	0.00
	g. *Anaerovorax*	0.01	0.00	0.00	0.00	0.00	0.00	-1.49	1.00	-1.83	1.00	-0.34	1.00	-0.17	1.00	0.06	1.00	0.24	1.00	2.92	1.00	1.60	1.00	1.02	1.00	3.09	1.00	1.36	1.00	-1.73	1.00
	g. *Mogibacterium*	0.20	0.20	0.24	0.13	0.24	0.20	0.32	1.00	0.62	0.60	0.30	1.00	0.98	0.52	0.92	0.33	-0.06	1.00	0.59	1.00	-0.07	1.00	0.29	1.00	-0.39	1.00	-0.01	1.00	0.38	1.00
	f. [Mogibacteriaceae]	0.39	0.25	0.30	0.23	0.33	0.36	0.65	0.90	0.83	0.41	0.18	1.00	0.69	1.00	0.79	0.86	0.10	1.00	-0.26	1.00	-0.30	1.00	-0.22	1.00	-0.95	0.41	-0.40	1.00	0.55	1.00
	g. *GW-34*	0.02	0.00	0.00	0.00	0.00	0.00	-1.30	1.00	-1.49	1.00	-0.19	1.00	-0.17	1.00	0.06	1.00	0.24	1.00	2.56	1.00	1.44	1.00	1.01	1.00	2.74	1.00	1.20	1.00	-1.54	1.00
	g. *Helcococcus*	0.01	0.00	0.00	0.00	0.00	0.00	-4.61	1.00	-5.34	1.00	-0.73	1.00	-0.83	1.00	-0.53	1.00	0.30	1.00	4.54	1.00	0.77	1.00	-0.27	1.00	5.38	1.00	0.47	1.00	-4.91	1.00
	f. [Tissierellaceae]	0.00	0.00	0.00	0.00	0.00	0.00	-0.62	1.00	-0.74	1.00	-0.11	1.00	-0.17	1.00	0.06	1.00	0.24	1.00	1.01	1.00	0.56	1.00	0.21	1.00	1.18	1.00	0.32	1.00	-0.86	1.00
	g. *Peptoniphilus*	0.00	0.00	0.00	0.00	0.00	0.00	-2.63	1.00	-2.80	1.00	-0.17	1.00	-0.61	1.00	-0.29	1.00	0.32	1.00	2.32	1.00	0.31	1.00	-0.18	1.00	2.94	1.00	-0.01	1.00	-2.95	1.00
	g. *Tissierella_Soehngenia*	0.00	0.00	0.00	0.00	0.00	0.00	-0.17	1.00	-0.22	1.00	-0.05	1.00	-0.83	1.00	-0.53	1.00	0.30	1.00	-0.64	1.00	0.01	1.00	-0.33	1.00	0.19	1.00	-0.29	1.00	-0.48	1.00
	g. *Christensenella*	0.05	0.02	0.01	0.02	0.02	0.01	-1.35	0.35	-1.92	0.05	-0.57	1.00	0.40	1.00	-0.14	1.00	-0.54	1.00	1.47	1.00	-0.28	1.00	-0.32	1.00	1.07	0.72	0.26	1.00	-0.81	1.00
	f. Christensenellaceae	0.56	0.00	0.00	0.03	0.01	0.00	-6.40	0.00	-9.21	0.00	-2.82	1.00	-1.20	1.00	-2.39	0.77	-1.18	1.00	3.56	1.00	-1.64	1.00	-3.27	1.00	4.76	0.01	-0.46	1.00	-5.22	0.28
	g. *Clostridium*	0.03	0.31	0.28	0.02	0.08	0.29	3.61	0.00	3.57	0.00	-0.05	1.00	2.79	0.19	4.82	0.00	2.03	1.00	2.33	1.00	3.15	0.09	1.07	1.00	-0.46	1.00	1.12	1.00	1.58	1.00
	f. Clostridiaceae	0.11	0.00	0.00	0.08	0.04	0.00	-5.15	0.01	-2.84	0.18	2.30	1.00	-0.33	1.00	-2.85	0.33	-2.52	1.00	-0.30	1.00	-5.11	0.09	-0.29	1.00	0.03	1.00	-2.59	1.00	-2.63	1.00
	g. *Tindallia_Anoxynatronum*	0.00	0.00	0.00	0.00	0.00	0.00	0.24	1.00	-0.35	1.00	-0.59	1.00	0.29	1.00	0.63	1.00	0.34	1.00	0.11	1.00	0.06	1.00	-0.86	1.00	-0.18	1.00	-0.28	1.00	-0.10	1.00
	g. *Dehalobacterium*	0.00	0.00	0.00	0.00	0.00	0.00	-1.61	1.00	-1.70	1.00	-0.08	1.00	1.42	1.00	0.64	1.00	-0.77	1.00	1.56	1.00	-1.47	1.00	-0.78	1.00	0.15	1.00	-0.69	1.00	-0.84	1.00
	f. Dehalobacteriaceae	0.03	0.00	0.00	0.01	0.00	0.00	-4.69	1.00	-4.69	1.00	-0.01	1.00	0.56	1.00	0.49	1.00	-0.07	1.00	3.76	1.00	-1.49	1.00	-1.43	1.00	3.20	1.00	-1.42	1.00	-4.62	1.00
	f. EtOH8	0.01	0.00	0.00	0.00	0.00	0.00	-4.37	1.00	-4.44	1.00	-0.07	1.00	-1.78	1.00	-2.34	1.00	-0.56	1.00	1.90	1.00	-0.69	1.00	-0.20	1.00	3.68	1.00	-0.13	1.00	-3.81	1.00
	g. *Anaerofustis*	0.00	0.00	0.00	0.00	0.00	0.00	-0.17	1.00	-0.22	1.00	-0.05	1.00	-0.83	1.00	-0.53	1.00	0.30	1.00	-0.60	1.00	0.05	1.00	-0.29	1.00	0.23	1.00	-0.25	1.00	-0.48	1.00
	g. *Pseudoramibacter_Eubacterium*	0.18	0.78	0.61	0.08	0.36	0.82	2.43	0.05	2.38	0.04	-0.05	1.00	2.10	0.48	5.01	0.00	2.91	0.14	1.88	1.00	2.21	0.32	-0.75	1.00	-0.22	1.00	-0.70	1.00	-0.47	1.00
	f. Gracilibacteraceae	0.00	0.00	0.00	0.00	0.00	0.00	-0.55	1.00	0.02	1.00	0.58	1.00	-1.59	1.00	-2.70	1.00	-1.11	1.00	-2.40	1.00	-1.37	1.00	0.32	1.00	-0.82	1.00	-0.26	1.00	0.56	1.00
	g. *Ruminococcus 1*	2.16	0.17	0.14	4.79	2.18	0.11	-4.65	0.00	-4.43	0.00	0.22	1.00	-0.72	1.00	-4.38	0.00	-3.66	0.06	-1.50	1.00	-5.44	0.00	-1.55	1.00	-0.79	1.00	-1.78	1.00	-0.99	1.00
	g. *[Ruminococcus]2*	0.02	0.00	0.00	0.04	0.00	0.01	-6.62	0.40	-0.17	1.00	6.45	1.00	-3.77	0.90	-1.15	1.00	2.61	1.00	-2.59	1.00	-5.44	0.53	-1.60	1.00	1.18	1.00	-8.05	1.00	-9.24	0.15
	g. *Anaerostipes*	0.00	0.00	0.00	0.00	0.01	0.00	0.83	1.00	-0.69	1.00	-1.52	1.00	0.32	1.00	-0.01	1.00	-0.33	1.00	0.68	1.00	1.19	1.00	0.00	1.00	0.36	1.00	1.52	1.00	1.16	1.00

	g. *Blautia*	0.04	0.01	0.01	0.04	0.01	0.04	-2.70	0.03	-2.13	0.09	0.57	1.00	-2.41	0.29	-0.72	1.00	1.69	1.00	0.28	1.00	-0.01	1.00	-1.13	1.00	2.69	0.04	-1.70	1.00	-4.39	0.09
	g. *Butyrivibrio*	2.06	2.48	2.90	3.05	2.40	2.79	1.23	0.43	1.81	0.05	0.58	1.00	-0.05	1.00	0.08	1.00	0.14	1.00	-1.12	1.00	0.17	1.00	0.61	1.00	-1.06	0.72	0.03	1.00	1.10	1.00
	g. *Coprococcus*	0.57	0.00	0.01	0.03	0.00	0.02	-7.58	0.00	-9.14	0.00	-1.57	1.00	-4.90	0.48	-5.67	0.15	-0.77	1.00	4.92	1.00	2.24	1.00	1.45	1.00	9.82	0.00	3.02	1.00	-6.80	0.43
	g. *Dorea*	0.02	0.01	0.00	0.06	0.02	0.01	-1.78	0.68	-3.75	0.03	-1.97	1.00	-0.87	1.00	-1.78	0.91	-0.91	1.00	-0.61	1.00	-1.52	1.00	-2.58	1.00	0.26	1.00	-0.61	1.00	-0.86	1.00
	g. *Epulopiscium*	0.00	0.00	0.00	0.00	0.00	0.00	-0.17	1.00	-0.22	1.00	-0.05	1.00	0.31	1.00	0.03	1.00	-0.29	1.00	-0.06	1.00	-0.54	1.00	-0.30	1.00	-0.37	1.00	-0.26	1.00	0.11	1.00
	g. *Lachnospira*	0.00	0.00	0.00	0.00	0.01	0.00	1.08	1.00	-0.36	1.00	-1.44	1.00	3.94	1.00	0.51	1.00	-3.43	1.00	0.32	1.00	-2.54	1.00	-0.55	1.00	-3.62	1.00	0.89	1.00	4.51	1.00
	g. *Moryella*	0.07	0.03	0.20	0.11	0.46	0.07	2.52	0.19	5.08	0.00	2.56	1.00	4.00	0.03	2.27	0.33	-1.73	1.00	-2.13	1.00	-3.61	0.09	0.68	1.00	-6.13	0.00	-1.88	1.00	4.25	0.17
	f. Lachnospiraceae	2.05	4.08	4.48	2.01	6.06	5.85	1.30	0.02	1.17	0.03	-0.13	1.00	1.45	0.07	1.61	0.00	0.15	1.00	-0.05	1.00	-0.20	1.00	-0.49	1.00	-1.50	0.01	-0.36	1.00	1.15	0.83
	g. *Pseudobutyrivibrio*	0.00	0.00	0.00	0.01	0.00	0.00	0.31	1.00	0.58	1.00	0.27	1.00	0.63	1.00	-0.17	1.00	-0.81	1.00	-0.89	1.00	-1.22	1.00	-0.14	1.00	-1.53	1.00	-0.41	1.00	1.12	1.00
	g. *Roseburia*	0.03	0.04	0.07	0.07	0.23	0.13	-0.64	1.00	-1.82	0.37	-1.18	1.00	2.02	0.63	1.50	0.91	-0.53	1.00	-0.24	1.00	-2.90	0.16	-3.56	0.18	-2.26	0.20	-2.37	1.00	-0.11	1.00
	g. *Shuttleworthia*	0.11	11.95	7.64	0.56	3.55	4.46	8.65	0.00	8.85	0.00	0.20	1.00	3.25	0.29	3.64	0.06	0.39	1.00	-3.39	1.00	2.01	1.00	1.82	1.00	-6.64	0.00	1.62	1.00	8.26	0.00
	o. Clostridiales	6.31	4.49	2.98	6.98	3.92	4.59	-0.26	1.00	-0.78	0.20	-0.52	1.00	-0.71	0.90	-0.01	1.00	0.70	1.00	0.15	1.00	0.60	1.00	-0.61	1.00	0.86	0.25	-0.10	1.00	-0.96	1.00
	g. *Peptococcus*	0.00	0.02	0.03	0.00	0.01	0.02	4.50	0.01	5.68	0.00	1.18	1.00	5.89	0.07	7.46	0.00	1.56	1.00	1.69	1.00	0.29	1.00	-0.09	1.00	-4.21	0.02	-1.27	1.00	2.94	1.00
	f. Peptostreptococcaceae	0.00	0.00	0.00	0.00	0.00	0.00	-0.26	1.00	0.16	1.00	0.42	1.00	-0.30	1.00	-0.55	1.00	-0.26	1.00	-0.52	1.00	-0.48	1.00	0.19	1.00	-0.22	1.00	-0.22	1.00	0.00	1.00
	g. *Peptostreptococcus*	0.00	0.00	0.00	0.00	0.00	0.00	-0.17	1.00	-0.22	1.00	-0.05	1.00	-0.21	1.00	0.61	1.00	0.82	1.00	0.01	1.00	0.04	1.00	-0.83	1.00	0.22	1.00	-0.78	1.00	-1.00	1.00
	g. *Anaerotruncus*	0.02	0.00	0.00	0.01	0.01	0.00	-3.22	0.16	-3.26	0.11	-0.04	1.00	-1.70	1.00	-3.42	0.33	-1.72	1.00	1.36	1.00	-0.15	1.00	1.53	1.00	3.07	0.25	1.57	1.00	-1.50	1.00
	g. *Faecalibacterium*	0.00	0.00	0.00	0.00	0.00	0.00	-0.61	1.00	-0.72	1.00	-0.11	1.00	1.14	1.00	0.76	1.00	-0.38	1.00	0.20	1.00	-1.55	1.00	-1.28	1.00	-0.94	1.00	-1.17	1.00	-0.23	1.00
	f. Ruminococcaceae	9.13	13.62	15.83	4.44	6.02	13.68	0.79	0.67	0.71	0.68	-0.08	1.00	0.56	1.00	1.77	0.01	1.21	1.00	1.18	1.00	1.42	0.24	0.13	1.00	0.63	1.00	0.20	1.00	-0.42	1.00
	g. *Oscillospira*	0.91	0.21	0.08	0.88	0.44	0.19	-2.43	0.00	-3.48	0.00	-1.05	1.00	-1.36	0.19	-2.21	0.00	-0.85	1.00	0.10	1.00	-0.97	0.88	-1.18	1.00	1.46	0.02	-0.12	1.00	-1.58	0.42
	g. *Syntrophomonas*	0.00	0.00	0.00	0.00	0.00	0.00	0.91	1.00	-0.35	1.00	-1.27	1.00	2.09	1.00	-0.59	1.00	-2.68	1.00	-0.33	1.00	-1.50	1.00	-0.09	1.00	-2.42	1.00	1.17	1.00	3.59	1.00
	g. *Acidaminococcus*	0.18	1.17	0.82	0.33	0.29	0.90	2.83	0.00	1.74	0.14	-1.09	1.00	0.07	1.00	2.57	0.03	2.51	0.23	-0.10	1.00	2.66	0.09	-0.93	1.00	-0.17	1.00	0.16	1.00	0.32	1.00
	g. *Anaerovibrio*	0.01	0.00	0.00	0.00	0.07	0.00	-1.94	1.00	-4.81	0.09	-2.87	1.00	4.98	0.19	-1.25	1.00	-6.23	0.10	2.49	1.00	-4.42	0.24	-1.07	1.00	-2.49	0.66	1.80	1.00	4.29	1.00
	g. *Dialister*	0.11	2.80	3.52	0.33	1.67	2.58	6.91	0.00	7.12	0.00	0.21	1.00	3.27	0.17	5.43	0.00	2.16	1.00	-1.16	1.00	2.48	0.32	0.53	1.00	-4.43	0.00	0.32	1.00	4.75	0.09
	g. *Megasphaera*	0.28	1.87	0.83	0.13	1.13	1.29	4.42	0.00	3.13	0.01	-1.29	1.00	3.18	0.07	4.14	0.00	0.96	1.00	0.36	1.00	1.60	1.00	-0.65	1.00	-2.82	0.02	0.64	1.00	3.46	0.18
	g. *Mitsuokella*	0.04	0.24	0.06	0.06	0.10	0.19	2.93	0.04	0.35	1.00	-2.57	1.00	2.11	0.63	3.12	0.05	1.00	1.00	0.45	1.00	1.26	1.00	-2.32	1.00	-1.67	0.66	0.26	1.00	1.92	1.00
	f. Veillonellaceae	0.02	0.27	0.20	0.10	0.38	0.28	3.57	0.00	3.07	0.01	-0.50	1.00	1.55	0.90	1.53	0.63	-0.01	1.00	-2.04	1.00	-0.02	1.00	-0.50	1.00	-3.58	0.00	0.00	1.00	3.58	0.17
	g. *Phascolarctobacterium*	0.20	0.00	0.00	0.05	0.00	0.00	-7.65	0.05	-7.60	0.04	0.05	1.00	-2.05	1.00	0.91	1.00	2.96	1.00	3.59	1.00	-2.00	1.00	-4.92	1.00	5.65	0.25	-4.97	1.00	-10.61	0.17
	g. *Selenomonas*	0.00	0.00	0.01	0.00	0.00	0.02	1.62	1.00	1.63	1.00	0.01	1.00	2.61	1.00	2.47	1.00	-0.13	1.00	-0.10	1.00	-1.09	1.00	-0.95	1.00	-2.70	1.00	-0.96	1.00	1.75	1.00
	g. *Succiniclasticum*	0.77	1.01	0.01	0.68	0.59	0.15	0.22	1.00	-7.63	0.00	-7.85	0.00	-0.71	1.00	-2.78	0.15	-2.08	1.00	0.35	1.00	1.28	1.00	-4.50	0.18	1.06	1.00	3.35	1.00	2.29	1.00
	g. *Veillonella*	0.40	0.00	0.01	0.00	0.00	0.00	-9.14	0.51	-7.74	0.63	1.40	1.00	-1.49	1.00	-1.16	1.00	0.32	1.00	9.67	1.00	2.02	1.00	3.10	1.00	11.16	0.36	1.69	1.00	-9.46	1.00
	g. *[Eubacterium]*	1.25	0.29	0.51	0.19	0.07	0.62	-1.26	0.90	-1.08	1.00	0.18	1.00	-1.02	1.00	2.43	0.21	3.45	0.10	2.86	1.00	2.62	0.25	-0.65	1.00	3.88	0.00	-0.83	1.00	-4.70	0.09
	g. *Asteroleplasma*	0.00	0.00	0.00	0.00	0.00	0.00	0.22	1.00	-0.25	1.00	-0.47	1.00	-0.17	1.00	0.06	1.00	0.24	1.00	0.55	1.00	0.95	1.00	0.24	1.00	0.73	1.00	0.71	1.00	-0.02	1.00
	g. *Bulleidia*	0.16	1.47	1.03	0.45	0.37	0.68	3.89	0.00	3.25	0.00	-0.63	1.00	0.78	1.00	2.30	0.01	1.52	1.00	-0.91	1.00	2.20	0.09	0.04	1.00	-1.69	0.12	0.68	1.00	2.36	0.29
	g. *Catenibacterium*	0.52	0.32	0.95	0.14	0.70	0.33	0.78	1.00	-1.07	1.00	-1.85	1.00	1.89	1.00	3.43	0.11	1.53	1.00	1.89	1.00	0.78	1.00	-2.61	1.00	0.00	1.00	-0.75	1.00	-0.75	1.00
	g. *Coprobacillus*	0.00	0.00	0.00	0.00	0.00	0.00	-0.17	1.00	-0.22	1.00	-0.05	1.00	-0.23	1.00	0.58	1.00	0.81	1.00	-0.21	1.00	-0.16	1.00	-1.02	1.00	0.02	1.00	-0.97	1.00	-0.99	1.00
	g. *Erysipelothrix*	0.00	0.00	0.00	0.00	0.00	0.00	-0.17	1.00	-0.22	1.00	-0.05	1.00	-0.59	1.00	-0.32	1.00	0.27	1.00	-0.84	1.00	-0.43	1.00	-0.75	1.00	-0.26	1.00	-0.70	1.00	-0.44	1.00
	g. *Holdemania*	0.00	0.00	0.00	0.00	0.00	0.00	-1.01	1.00	-1.08	1.00	-0.06	1.00	-1.38	1.00	-0.99	1.00	0.38	1.00	0.36	1.00	0.72	1.00	0.28	1.00	1.74	1.00	0.34	1.00	-1.40	1.00
	g. *L7A_E11*	0.00	0.00	0.00	0.03	0.01	0.00	-0.17	1.00	-0.22	1.00	-0.05	1.00	-0.23	1.00	-1.66	1.00	-1.42	1.00	-2.68	1.00	-2.62	1.00	-1.25	1.00	-2.45	1.00	-1.20	1.00	1.25	1.00
	f. Erysipelotrichaceae	0.05	0.01	0.02	0.01	0.01	0.02	-2.76	0.31	-4.20	0.03	-1.44	1.00	0.17	1.00	1.52	1.00	1.35	1.00	4.18	1.00	1.25	1.00	-1.54	1.00	4.01	0.07	-0.10	1.00	-4.11	0.83
	g. *p-75-a5*	0.24	0.01	0.01	1.75	0.04	0.02	-0.61	1.00	-0.17	1.00	0.44	1.00	-1.50	1.00	-1.49	1.00	0.01	1.00	-2.80	1.00	-1.91	1.00	-1.48	1.00	-1.30	1.00	-1.92	1.00	-0.62	1.00
	g. *RFN20*	1.75	0.59	0.04	0.35	6.41	0.40	-0.68	1.00	-4.00	0.00	-3.32	0.53	3.82	0.03	-0.81	1.00	-4.64	0.00	0.62	1.00	-3.88	0.02	-2.57	1.00	-3.20	0.02	0.75	1.00	3.96	0.17
	g. *Sharpea*	0.27	9.85	25.72	0.36	2.57	21.37	4.99	0.00	8.72	0.00	3.73	0.12	1.78	0.90	6.47	0.00	4.69	0.00	-1.25	1.00	1.96	0.88	1.00	1.00	-3.03	0.03	-2.73	1.00	0.30	1.00
Fusobacteria	g. *Fusobacterium*	0.00	0.00	0.00	0.00	0.00	0.01	-1.98	1.00	-2.00	1.00	-0.02	1.00	-1.88	1.00	-0.53	1.00	1.35	1.00	-0.05	1.00	-0.16	1.00	-1.52	1.00	1.83	1.00	-1.50	1.00	-3.33	1.00
Gemmatimonadetes	c. Gemm-3	0.00	0.00	0.00	0.00	0.00	0.00	-0.08	1.00	-0.02	1.00	0.07	1.00	-0.51	1.00	0.62	1.00	1.13	1.00	-0.26	1.00	0.17	1.00	-0.90	1.00	0.25	1.00	-0.96	1.00	-1.21	1.00
Lentisphaerae	f. Victivallaceae	0.02	0.00	0.00	0.02	0.00	0.00	-4.72	0.06	-4.14	0.09	0.59	1.00	-2.12	1.00	-2.82	1.00	-0.70	1.00	1.51	1.00	-1.09	1.00	0.20	1.00	3.63	0.26	-0.39	1.00	-4.02	1.00
OP11		0.00	0.00	0.00	0.00	0.00	0.00	-0.32	1.00	0.29	1.00	0.62	1.00	-0.17	1.00	0.06	1.00	0.24	1.00	0.44	1.00	0.29	1.00	0.66	1.00	0.61	1.00	0.05	1.00	-0.56	1.00
Planctomycetes		0.02	0.00	0.00	0.02	0.00	0.00	-3.43	0.66	-3.40	1.00	0.03	1.00	-1.21	0.98	-0.69	0.99	0.52	0.96	2.26	0.99	0.04	1.00	-0.45	0.93	3.46	0.83	-0.48	1.00	-3.94	0.97
	f. Pirellulaceae	0.01	0.00	0.00	0.02	0.00	0.00	-1.51	1.00	-1.57	1.00	-0.07	1.00	-0.85	1.00	-0.55	1.00	0.30	1.00	0.95	1.00	0.30	1.00	-0.07	1.00	1.81	1.00	0.00	1.00	-1.81	1.00

	g. *Planctomyces*	0.00	0.00	0.00	0.00	0.00	0.00	-2.34	1.00	-2.39	1.00	-0.04	1.00	-0.82	1.00	-0.52	1.00	0.30	1.00	1.82	1.00	0.29	1.00	-0.05	1.00	2.64	1.00	-0.01	1.00	-2.64	1.00
Proteobacteria		6.18	6.09	4.90	4.67	7.10	7.22	0.26	0.66	-0.22	1.00	-0.48	1.00	0.04	0.98	0.27	0.99	0.24	0.96	-0.10	0.99	0.13	1.00	-0.58	0.83	-0.13	0.90	-0.11	1.00	0.03	0.97
	o. BD7-3	0.00	0.00	0.00	0.00	0.00	0.00	-3.23	1.00	-3.22	1.00	0.01	1.00	0.07	1.00	0.37	1.00	0.31	1.00	2.18	1.00	-1.11	1.00	-1.41	1.00	2.11	1.00	-1.42	1.00	-3.53	1.00
	g. *Asticcacaulis*	0.00	0.00	0.00	0.00	0.00	0.00	-1.03	1.00	-1.09	1.00	-0.06	1.00	-0.17	1.00	0.06	1.00	0.24	1.00	1.80	1.00	0.95	1.00	0.64	1.00	1.97	1.00	0.71	1.00	-1.27	1.00
	g. *Brevundimonas*	0.02	0.00	0.00	0.01	0.00	0.00	-4.98	0.24	-5.16	0.17	-0.17	1.00	-2.90	1.00	-3.61	1.00	-0.71	1.00	1.93	1.00	-0.16	1.00	0.38	1.00	4.83	0.37	0.55	1.00	-4.27	1.00
	g. *Caulobacter*	0.00	0.00	0.00	0.00	0.00	0.00	-0.63	1.00	-0.61	1.00	0.01	1.00	-0.68	1.00	-0.63	1.00	0.05	1.00	0.31	1.00	0.36	1.00	0.32	1.00	0.99	1.00	0.31	1.00	-0.68	1.00
	g. *Mycoplana*	0.01	0.00	0.00	0.00	0.00	0.00	-2.86	1.00	-2.83	1.00	0.03	1.00	0.10	1.00	0.39	1.00	0.29	1.00	2.48	1.00	-0.48	1.00	-0.74	1.00	2.38	1.00	-0.77	1.00	-3.15	1.00
	f. Caulobacteraceae	0.01	0.00	0.00	0.00	0.00	0.00	-2.16	1.00	-1.87	1.00	0.29	1.00	-2.02	1.00	-1.91	1.00	0.11	1.00	0.61	1.00	0.47	1.00	0.65	1.00	2.63	1.00	0.36	1.00	-2.27	1.00
	g. *Phenylobacterium*	0.00	0.00	0.00	0.00	0.00	0.00	-0.52	1.00	0.82	1.00	1.34	1.00	-0.17	1.00	0.06	1.00	0.24	1.00	0.59	1.00	0.24	1.00	1.35	1.00	0.76	1.00	0.00	1.00	-0.76	1.00
	c. Alphaproteobacteria	0.04	0.00	0.00	0.01	0.00	0.00	-6.35	1.00	-6.49	0.92	-0.14	1.00	0.06	1.00	0.26	1.00	0.20	1.00	5.44	1.00	-0.97	1.00	-1.32	1.00	5.38	1.00	-1.17	1.00	-6.55	1.00
	o. RF32	1.57	0.01	0.02	0.39	0.19	0.08	-10.50	0.00	-5.18	0.01	5.32	0.76	-3.14	0.63	-1.91	1.00	1.23	1.00	3.39	1.00	-3.97	0.54	0.12	1.00	6.53	0.00	-5.20	1.00	-11.73	0.00
	f. Aurantimonadaceae	0.00	0.00	0.00	0.00	0.00	0.00	-0.35	1.00	-1.03	1.00	-0.68	1.00	-0.77	1.00	-0.51	1.00	0.26	1.00	0.42	1.00	0.84	1.00	-0.10	1.00	1.19	1.00	0.58	1.00	-0.61	1.00
	f. Bartonellaceae	0.00	0.00	0.00	0.00	0.00	0.00	-0.80	1.00	-0.92	1.00	-0.12	1.00	-0.17	1.00	0.06	1.00	0.24	1.00	1.00	1.00	0.38	1.00	0.02	1.00	1.18	1.00	0.14	1.00	-1.04	1.00
	f. Beijerinckiaceae	0.01	0.00	0.00	0.01	0.00	0.00	-4.90	1.00	-4.38	1.00	0.51	1.00	-2.17	1.00	-2.80	1.00	-0.63	1.00	1.78	1.00	-0.95	1.00	0.20	1.00	3.94	1.00	-0.32	1.00	-4.26	1.00
	g. *Bosea*	0.00	0.00	0.00	0.00	0.00	0.00	-0.17	1.00	-0.22	1.00	-0.05	1.00	-0.63	1.00	-0.52	1.00	0.11	1.00	-0.54	1.00	-0.09	1.00	-0.24	1.00	0.08	1.00	-0.20	1.00	-0.28	1.00
	f. Bradyrhizobiaceae	0.00	0.00	0.00	0.00	0.00	0.00	-1.50	1.00	-1.35	1.00	0.14	1.00	-0.60	1.00	-0.38	1.00	0.22	1.00	1.01	1.00	0.11	1.00	0.04	1.00	1.61	1.00	-0.11	1.00	-1.72	1.00
	f. Brucellaceae	0.02	0.00	0.00	0.02	0.00	0.00	-4.99	1.00	-4.79	1.00	0.20	1.00	-1.79	1.00	-2.42	1.00	-0.63	1.00	2.37	1.00	-0.83	1.00	0.00	1.00	4.16	1.00	-0.19	1.00	-4.35	1.00
	g. *Ochrobactrum*	0.01	0.00	0.00	0.01	0.00	0.00	-4.70	1.00	-4.90	1.00	-0.20	1.00	-4.08	1.00	-4.39	1.00	-0.30	1.00	1.16	1.00	0.54	1.00	0.65	1.00	5.24	1.00	0.85	1.00	-4.39	1.00
	g. *Devosia*	0.02	0.00	0.00	0.01	0.00	0.00	-4.01	0.12	-4.80	0.05	-0.78	1.00	-2.58	1.00	-2.67	0.73	-0.09	1.00	-0.03	1.00	-1.46	1.00	-2.15	1.00	2.55	0.66	-1.37	1.00	-3.92	1.00
	f. Hyphomicrobiaceae	0.00	0.00	0.00	0.00	0.00	0.00	-0.53	1.00	-0.42	1.00	0.11	1.00	-0.67	1.00	-0.61	1.00	0.06	1.00	0.16	1.00	0.30	1.00	0.35	1.00	0.83	1.00	0.24	1.00	-0.59	1.00
	g. *Methylobacterium*	0.00	0.00	0.00	0.00	0.00	0.00	-0.17	1.00	-0.22	1.00	-0.05	1.00	-0.72	1.00	-0.48	1.00	0.24	1.00	-0.54	1.00	0.00	1.00	-0.28	1.00	0.18	1.00	-0.24	1.00	-0.41	1.00
	o. Rhizobiales	0.01	0.00	0.00	0.00	0.00	0.00	-3.96	1.00	-3.08	1.00	0.87	1.00	0.20	1.00	-0.32	1.00	-0.52	1.00	3.47	1.00	-0.69	1.00	0.71	1.00	3.27	1.00	-0.17	1.00	-3.43	1.00
	g. *Aminobacter*	0.00	0.00	0.00	0.00	0.00	0.00	-0.62	1.00	-0.73	1.00	-0.11	1.00	-0.17	1.00	0.06	1.00	0.24	1.00	0.95	1.00	0.50	1.00	0.15	1.00	1.12	1.00	0.26	1.00	-0.86	1.00
	f. Phyllobacteriaceae	0.02	0.00	0.00	0.01	0.00	0.00	-3.67	0.13	-4.29	0.05	-0.62	1.00	-1.30	1.00	-4.13	0.26	-2.83	1.00	0.24	1.00	-2.13	1.00	0.08	1.00	1.54	1.00	0.71	1.00	-0.83	1.00
	g. *Agrobacterium*	0.01	0.01	0.00	0.00	0.00	0.00	-0.41	1.00	-1.67	1.00	-1.26	1.00	-0.20	1.00	-1.37	1.00	-1.18	1.00	2.05	1.00	1.84	1.00	1.75	1.00	2.25	1.00	3.01	1.00	0.77	1.00
	g. *Kaistia*	0.00	0.00	0.00	0.00	0.00	0.00	-0.63	1.00	-0.74	1.00	-0.11	1.00	0.04	1.00	-0.53	1.00	-0.57	1.00	0.30	1.00	-0.37	1.00	0.09	1.00	0.26	1.00	0.20	1.00	-0.06	1.00
	f. Rhizobiaceae	0.00	0.00	0.00	0.00	0.00	0.00	0.38	1.00	-0.27	1.00	-0.65	1.00	-0.17	1.00	0.06	1.00	0.24	1.00	0.46	1.00	1.01	1.00	0.12	1.00	0.63	1.00	0.77	1.00	0.14	1.00
	f. Rhodobacteraceae	0.03	0.00	0.00	0.01	0.01	0.00	-6.83	0.08	-5.91	0.12	0.92	1.00	-0.96	1.00	-1.97	1.00	-1.02	1.00	3.00	1.00	-2.87	1.00	-0.94	1.00	3.96	0.66	-1.86	1.00	-5.82	1.00
	g. *Paracoccus*	0.02	0.00	0.00	0.02	0.00	0.00	-5.68	0.01	-5.59	0.01	0.09	1.00	-3.88	0.20	-3.83	0.14	0.05	1.00	0.74	1.00	-1.05	1.00	-1.02	1.00	4.62	0.01	-1.11	1.00	-5.73	0.29
	f. mitochondria	0.01	0.02	0.03	0.00	0.01	0.02	2.22	0.06	2.77	0.01	0.55	1.00	1.95	0.76	3.35	0.01	1.40	1.00	1.25	1.00	1.52	0.89	0.67	1.00	-0.70	1.00	0.12	1.00	0.82	1.00
	f. Erythrobacteraceae	0.00	0.00	0.00	0.00	0.00	0.00	0.21	1.00	-0.45	1.00	-0.66	1.00	-0.17	1.00	0.06	1.00	0.24	1.00	1.14	1.00	1.53	1.00	0.63	1.00	1.32	1.00	1.29	1.00	-0.03	1.00
	o. Sphingomonadales	0.00	0.00	0.00	0.00	0.00	0.00	-0.17	1.00	-0.22	1.00	-0.05	1.00	-0.83	1.00	-0.53	1.00	0.30	1.00	-0.60	1.00	0.05	1.00	-0.29	1.00	0.23	1.00	-0.25	1.00	-0.48	1.00
	g. *Kaistobacter*	0.00	0.00	0.00	0.00	0.00	0.00	-0.85	1.00	-0.78	1.00	0.07	1.00	-0.17	1.00	0.06	1.00	0.24	1.00	0.94	1.00	0.26	1.00	0.09	1.00	1.11	1.00	0.02	1.00	-1.09	1.00
	f. Sphingomonadaceae	0.00	0.00	0.00	0.00	0.00	0.00	-1.81	1.00	-2.44	1.00	-0.64	1.00	-1.34	1.00	-2.44	1.00	-1.11	1.00	0.23	1.00	-0.24	1.00	0.23	1.00	1.57	1.00	0.87	1.00	-0.70	1.00
	g. *Novosphingobium*	0.00	0.00	0.00	0.00	0.00	0.00	-1.22	1.00	-1.27	1.00	-0.05	1.00	-0.17	1.00	0.06	1.00	0.24	1.00	1.76	1.00	0.72	1.00	0.43	1.00	1.94	1.00	0.48	1.00	-1.46	1.00
	g. *Sphingobium*	0.01	0.00	0.00	0.00	0.00	0.00	-3.68	1.00	-3.10	1.00	0.58	1.00	-0.10	1.00	-0.89	1.00	-0.79	1.00	1.70	1.00	-1.88	1.00	-0.51	1.00	1.80	1.00	-1.09	1.00	-2.89	1.00
	g. *Sphingomonas*	0.00	0.00	0.00	0.00	0.00	0.00	-0.65	1.00	-1.47	1.00	-0.82	1.00	-1.52	1.00	-1.88	1.00	-0.37	1.00	-0.89	1.00	-0.02	1.00	-0.47	1.00	0.63	1.00	0.35	1.00	-0.28	1.00
	g. *Sphingopyxis*	0.02	0.00	0.00	0.03	0.01	0.00	-6.01	0.15	-6.11	0.11	-0.10	1.00	-2.09	1.00	-4.76	0.63	-2.68	1.00	0.88	1.00	-3.05	1.00	-0.47	1.00	2.96	1.00	-0.37	1.00	-3.34	1.00
	g. *Achromobacter*	0.00	0.00	0.00	0.00	0.00	0.00	-0.73	1.00	-0.83	1.00	-0.10	1.00	-0.23	1.00	0.09	1.00	0.32	1.00	-0.17	1.00	-0.67	1.00	-1.09	1.00	0.06	1.00	-0.99	1.00	-1.04	1.00
	f. Alcaligenaceae	0.73	0.00	0.00	0.66	0.03	0.00	-9.21	0.00	-10.06	0.00	-0.85	1.00	-3.33	0.19	-6.50	0.00	-3.16	0.98	1.53	1.00	-4.35	0.09	-2.03	1.00	4.86	0.00	-1.18	1.00	-6.05	0.09
	g. *Oligella*	0.00	0.00	0.00	0.00	0.00	0.00	-2.12	1.00	-2.11	1.00	0.01	1.00	-0.43	1.00	-1.19	1.00	-0.76	1.00	-0.21	1.00	-1.89	1.00	-1.13	1.00	0.22	1.00	-1.14	1.00	-1.36	1.00
	g. *Pigmentiphaga*	0.00	0.00	0.00	0.00	0.00	0.00	-0.72	1.00	-0.82	1.00	-0.10	1.00	-0.17	1.00	0.06	1.00	0.24	1.00	1.01	1.00	0.46	1.00	0.12	1.00	1.18	1.00	0.23	1.00	-0.96	1.00
	g. *Sutterella*	0.22	0.00	0.00	0.05	0.01	0.01	-5.91	0.00	-5.56	0.00	0.35	1.00	-2.72	0.76	-2.58	0.61	0.14	1.00	1.88	1.00	-1.32	1.00	-1.10	1.00	4.59	0.01	-1.46	1.00	-6.05	0.17
	g. *Lautropia*	0.02	0.00	0.00	0.00	0.00	0.00	-1.56	1.00	-1.03	1.00	0.53	1.00	-0.12	1.00	-0.49	1.00	-0.37	1.00	2.06	1.00	0.62	1.00	1.52	1.00	2.18	1.00	0.99	1.00	-1.19	1.00
	g. *Acidovorax*	0.00	0.00	0.00	0.00	0.00	0.00	-0.62	1.00	-0.73	1.00	-0.11	1.00	-0.17	1.00	0.06	1.00	0.24	1.00	0.95	1.00	0.50	1.00	0.15	1.00	1.12	1.00	0.26	1.00	-0.86	1.00
	g. *Comamonas*	0.61	0.03	0.01	0.26	0.04	0.01	-4.71	0.00	-6.28	0.00	-1.57	1.00	-2.84	0.19	-3.35	0.01	-0.51	1.00	1.15	1.00	-0.72	1.00	-1.78	1.00	3.99	0.00	-0.22	1.00	-4.20	0.15
	g. *Delftia*	0.01	0.00	0.00	0.00	0.00	0.00	-4.49	1.00	-2.89	1.00	1.61	1.00	-0.13	1.00	2.25	1.00	2.38	1.00	3.99	1.00	-0.37	1.00	-1.14	1.00	4.12	1.00	-2.75	1.00	-6.87	1.00
	g. *Hylemonella*	0.00	0.00	0.00	0.00	0.00	0.00	-0.46	1.00	-0.46	1.00	0.00	1.00	-1.24	1.00	-0.91	1.00	0.33	1.00	-0.42	1.00	0.36	1.00	0.03	1.00	0.81	1.00	0.03	1.00	-0.78	1.00

g. *Limnohabitans*	0.00	0.00	0.00	0.00	0.00	0.00	-0.61	1.00	-0.60	1.00	0.01	1.00	-0.68	1.00	0.24	1.00	0.92	1.00	-0.15	1.00	-0.09	1.00	-0.99	1.00	0.53	1.00	-1.01	1.00	-1.53	1.00
f. Comamonadaceae	0.03	0.01	0.00	0.03	0.01	0.01	-3.21	0.19	-4.32	0.03	-1.12	1.00	-0.55	1.00	-0.79	1.00	-0.24	1.00	1.59	1.00	-1.06	1.00	-1.94	1.00	2.14	0.72	-0.82	1.00	-2.97	1.00
g. *Ramlibacter*	0.00	0.00	0.00	0.00	0.00	0.00	-0.17	1.00	-0.22	1.00	-0.05	1.00	-0.23	1.00	0.57	1.00	0.81	1.00	-0.09	1.00	-0.04	1.00	-0.89	1.00	0.14	1.00	-0.84	1.00	-0.98	1.00
o. Burkholderiales	0.00	0.00	0.00	0.00	0.00	0.00	-2.30	1.00	-2.28	1.00	0.02	1.00	-0.17	1.00	0.06	1.00	0.24	1.00	2.58	1.00	0.45	1.00	0.24	1.00	2.76	1.00	0.21	1.00	-2.54	1.00
g. *Janthinobacterium*	0.00	0.00	0.00	0.00	0.00	0.00	-0.65	1.00	-0.23	1.00	0.42	1.00	-0.17	1.00	0.06	1.00	0.24	1.00	0.96	1.00	0.49	1.00	0.67	1.00	1.14	1.00	0.25	1.00	-0.89	1.00
f. Oxalobacteraceae	0.00	0.01	0.00	0.00	0.00	0.00	1.75	1.00	-3.75	1.00	-5.50	1.00	-21.80	1.00	-7.26	1.00	14.54	0.98	5.23	1.00	28.78	1.00	8.74	1.00	27.03	1.00	14.24	1.00	-12.79	1.00
g. *Oxalobacter*	0.01	0.01	0.01	0.01	0.01	0.01	0.04	1.00	-1.72	0.40	-1.77	1.00	-1.65	1.00	-0.31	1.00	1.34	1.00	0.23	1.00	1.92	1.00	-1.18	1.00	1.88	0.59	0.59	1.00	-1.29	1.00
f. Methylophilaceae	0.00	0.00	0.00	0.00	0.00	0.00	-1.22	1.00	-1.27	1.00	-0.05	1.00	-0.82	1.00	-1.32	1.00	-0.50	1.00	0.22	1.00	-0.18	1.00	0.27	1.00	1.04	1.00	0.32	1.00	-0.72	1.00
c. Betaproteobacteria	0.00	0.00	0.00	0.00	0.00	0.00	0.72	1.00	0.91	1.00	0.18	1.00	-0.07	1.00	1.13	1.00	1.19	1.00	-0.38	1.00	0.42	1.00	-0.60	1.00	-0.31	1.00	-0.78	1.00	-0.47	1.00
g. *Kingella*	0.00	0.00	0.00	0.00	0.00	0.00	-23.34	1.00	-23.50	1.00	-0.16	1.00	-0.36	1.00	-20.84	1.00	-20.48	0.09	-4.40	1.00	-27.37	1.00	-7.06	1.00	-4.03	1.00	-6.90	1.00	-2.86	1.00
f. Neisseriaceae	0.08	0.01	0.00	0.03	0.01	0.00	-4.16	0.00	-5.34	0.00	-1.17	1.00	-0.81	1.00	-3.84	0.04	-3.03	1.00	1.23	1.00	-2.13	1.00	-0.26	1.00	2.04	0.34	0.91	1.00	-1.13	1.00
g. *Neisseria*	0.00	0.00	0.00	0.00	0.00	0.00	-0.78	1.00	-0.90	1.00	-0.12	1.00	-0.17	1.00	0.06	1.00	0.24	1.00	0.94	1.00	0.33	1.00	-0.03	1.00	1.11	1.00	0.09	1.00	-1.02	1.00
g. *Vogesella*	0.00	0.00	0.00	0.00	0.00	0.00	-0.17	1.00	-0.22	1.00	-0.05	1.00	-0.63	1.00	-0.52	1.00	0.11	1.00	-0.54	1.00	-0.09	1.00	-0.24	1.00	0.08	1.00	-0.20	1.00	-0.28	1.00
f. Rhodocyclaceae	0.00	0.00	0.00	0.00	0.00	0.01	0.76	1.00	0.97	1.00	0.21	1.00	-0.27	1.00	2.34	1.00	2.61	1.00	0.78	1.00	1.80	1.00	-0.59	1.00	1.05	1.00	-0.80	1.00	-1.85	1.00
g. *Bacteriovorax*	0.00	0.00	0.00	0.00	0.00	0.00	-1.52	1.00	-1.56	1.00	-0.03	1.00	-0.17	1.00	0.06	1.00	0.24	1.00	1.59	1.00	0.24	1.00	-0.03	1.00	1.76	1.00	0.00	1.00	-1.76	1.00
f. Bacteriovoracaceae	0.00	0.00	0.00	0.01	0.00	0.00	-3.25	1.00	-3.24	1.00	0.01	1.00	-0.64	1.00	-0.45	1.00	0.19	1.00	2.37	1.00	-0.24	1.00	-0.42	1.00	3.01	1.00	-0.43	1.00	-3.44	1.00
g. *Bdellovibrio*	0.00	0.00	0.00	0.00	0.00	0.00	-0.82	1.00	-0.88	1.00	-0.06	1.00	-0.17	1.00	0.06	1.00	0.24	1.00	0.94	1.00	0.29	1.00	0.00	1.00	1.11	1.00	0.05	1.00	-1.06	1.00
g. *Bilophila*	0.03	0.00	0.00	0.02	0.00	0.00	-2.82	0.72	-6.14	0.03	-3.32	1.00	-5.21	0.45	-4.81	0.27	0.41	1.00	0.20	1.00	2.60	1.00	-1.13	1.00	5.42	0.12	2.19	1.00	-3.22	1.00
g. *Desulfovibrio*	0.93	2.76	1.74	0.90	1.93	2.16	1.46	0.16	0.72	1.00	-0.74	1.00	1.07	1.00	1.58	0.21	0.51	1.00	0.53	1.00	0.92	1.00	-0.34	1.00	-0.54	1.00	0.40	1.00	0.95	1.00
f. Desulfovibrionaceae	0.00	0.00	0.00	0.00	0.00	0.00	-0.54	1.00	-2.65	1.00	-2.11	1.00	0.94	1.00	-1.04	1.00	-1.98	1.00	1.58	1.00	0.10	1.00	-0.03	1.00	0.64	1.00	2.08	1.00	1.44	1.00
o. Myxococcales	0.00	0.00	0.00	0.00	0.00	0.00	0.20	1.00	-0.38	1.00	-0.58	1.00	-0.07	1.00	0.78	1.00	0.85	1.00	0.49	1.00	0.76	1.00	-0.66	1.00	0.57	1.00	-0.09	1.00	-0.66	1.00
f. OM27	0.00	0.00	0.00	0.00	0.00	0.00	-0.82	1.00	-0.88	1.00	-0.06	1.00	-0.17	1.00	0.06	1.00	0.24	1.00	0.94	1.00	0.29	1.00	0.00	1.00	1.11	1.00	0.05	1.00	-1.06	1.00
g. *Campylobacter*	0.55	0.05	0.00	0.08	0.03	0.00	-3.72	0.06	-9.25	0.00	-5.54	0.53	-0.83	1.00	-3.24	0.26	-2.41	1.00	2.63	1.00	-0.25	1.00	-3.38	1.00	3.46	0.11	2.15	1.00	-1.31	1.00
g. *Helicobacter*	0.00	0.00	0.00	0.00	0.00	0.00	-0.35	1.00	0.15	1.00	0.50	1.00	-0.17	1.00	0.06	1.00	0.24	1.00	0.46	1.00	0.29	1.00	0.55	1.00	0.64	1.00	0.05	1.00	-0.58	1.00
f. Helicobacteraceae	0.00	0.00	0.00	0.00	0.00	0.00	-0.23	1.00	-0.54	1.00	-0.30	1.00	-0.17	1.00	0.06	1.00	0.24	1.00	0.74	1.00	0.68	1.00	0.14	1.00	0.91	1.00	0.44	1.00	-0.47	1.00
f. Aeromonadaceae	0.00	0.00	0.00	0.00	0.01	0.00	-1.39	1.00	-1.39	1.00	0.00	1.00	0.00	1.00	-0.87	1.00	-0.87	1.00	-1.06	1.00	-2.45	1.00	-1.58	1.00	-1.05	1.00	-1.58	1.00	-0.53	1.00
f. Succinivibrionaceae	0.15	2.40	0.71	0.44	2.87	4.63	3.97	0.00	2.37	0.02	-1.60	1.00	3.80	0.00	4.66	0.00	0.87	1.00	-0.35	1.00	-0.18	1.00	-2.64	0.18	-4.14	0.00	-1.04	1.00	3.10	0.17
g. *Ruminobacter*	0.07	0.00	0.00	0.21	0.01	0.00	-5.89	1.00	-6.60	0.74	-0.71	1.00	-7.24	1.00	-7.84	0.91	-0.60	1.00	-1.57	1.00	-0.22	1.00	-0.33	1.00	5.67	1.00	0.38	1.00	-5.29	1.00
g. *Succinivibrio*	0.19	0.67	2.28	1.17	1.76	0.20	1.99	0.17	2.90	0.01	0.91	1.00	2.09	0.48	-1.53	0.73	-3.63	0.02	-1.70	1.00	-1.80	0.88	2.73	0.44	-3.79	0.00	1.83	1.00	5.62	0.00
g. *Rheinheimera*	0.00	0.00	0.00	0.00	0.00	0.00	-0.17	1.00	-0.22	1.00	-0.05	1.00	-2.09	1.00	-1.82	1.00	0.27	1.00	-1.89	1.00	0.03	1.00	-0.29	1.00	0.20	1.00	-0.24	1.00	-0.45	1.00
g. *Candidatus Endobugula*	0.00	0.00	0.00	0.00	0.00	0.00	-0.17	1.00	-0.22	1.00	-0.05	1.00	-0.72	1.00	-0.48	1.00	0.24	1.00	-0.54	1.00	0.00	1.00	-0.28	1.00	0.18	1.00	-0.24	1.00	-0.41	1.00
g. *Cellvibrio*	0.01	0.00	0.00	0.00	0.00	0.00	-4.11	1.00	-4.18	1.00	-0.07	1.00	-1.03	1.00	-1.94	1.00	-0.91	1.00	1.79	1.00	-1.29	1.00	-0.45	1.00	2.82	1.00	-0.38	1.00	-3.20	1.00
f. Alteromonadaceae	0.01	0.00	0.00	0.00	0.00	0.00	-1.80	1.00	-2.27	1.00	-0.47	1.00	-1.59	1.00	-2.28	1.00	-0.69	1.00	0.45	1.00	0.24	1.00	0.46	1.00	2.04	1.00	0.93	1.00	-1.11	1.00
f. Idiomarinaceae	0.00	0.00	0.00	0.00	0.00	0.00	-0.17	1.00	-0.22	1.00	-0.05	1.00	-1.50	1.00	-1.20	1.00	0.31	1.00	-1.50	1.00	-0.17	1.00	-0.52	1.00	0.01	1.00	-0.47	1.00	-0.48	1.00
Pseudidiomarina	0.00	0.00	0.00	0.00	0.00	0.00	-0.17	1.00	-0.22	1.00	-0.05	1.00	-1.34	1.00	-1.30	1.00	0.04	1.00	-1.44	1.00	-0.27	1.00	-0.36	1.00	-0.10	1.00	-0.31	1.00	-0.22	1.00
f. OM60	0.00	0.00	0.00	0.00	0.00	0.00	-0.62	1.00	-0.61	1.00	0.01	1.00	-0.17	1.00	0.06	1.00	0.24	1.00	1.01	1.00	0.56	1.00	0.34	1.00	1.19	1.00	0.32	1.00	-0.86	1.00
g. *Citrobacter*	0.00	0.00	0.00	0.00	0.00	0.00	-0.30	1.00	0.59	1.00	0.90	1.00	0.27	1.00	0.62	1.00	0.35	1.00	0.75	1.00	0.17	1.00	0.72	1.00	0.48	1.00	-0.18	1.00	-0.66	1.00
g. *Erwinia*	0.00	0.00	0.00	0.00	0.00	0.00	-0.29	1.00	-1.06	1.00	-0.77	1.00	-0.15	1.00	-0.73	1.00	-0.58	1.00	-0.38	1.00	-0.51	1.00	-0.70	1.00	-0.22	1.00	0.07	1.00	0.29	1.00
g. *Escherichia*	0.00	0.00	0.00	0.00	0.00	0.00	-0.61	1.00	-0.72	1.00	-0.11	1.00	-0.17	1.00	0.06	1.00	0.24	1.00	0.84	1.00	0.40	1.00	0.06	1.00	1.02	1.00	0.16	1.00	-0.85	1.00
g. *Morganella*	0.00	0.00	0.00	0.00	0.00	0.00	-0.17	1.00	-0.22	1.00	-0.05	1.00	-0.63	1.00	-0.52	1.00	0.11	1.00	-0.54	1.00	-0.09	1.00	-0.24	1.00	0.08	1.00	-0.20	1.00	-0.28	1.00
f. Enterobacteriaceae	0.01	0.00	0.03	0.01	0.00	0.00	-3.60	0.18	-1.05	1.00	2.54	1.00	-0.72	1.00	-1.66	1.00	-0.94	1.00	0.33	1.00	-2.54	1.00	0.94	1.00	1.05	1.00	-1.60	1.00	-2.66	1.00
g. *Pantoea*	0.00	0.00	0.00	0.00	0.00	0.00	-0.72	1.00	-0.82	1.00	-0.10	1.00	0.30	1.00	0.03	1.00	-0.27	1.00	0.84	1.00	-0.17	1.00	0.00	1.00	0.54	1.00	0.10	1.00	-0.44	1.00
g. *Serratia*	0.00	0.00	0.00	0.00	0.00	0.00	-0.17	1.00	-0.22	1.00	-0.05	1.00	1.58	1.00	0.06	1.00	-1.53	1.00	0.18	1.00	-1.58	1.00	-0.10	1.00	-1.41	1.00	-0.06	1.00	1.35	1.00
f. HTCC2089	0.00	0.00	0.00	0.00	0.00	0.00	-0.17	1.00	-0.22	1.00	-0.05	1.00	0.48	1.00	0.16	1.00	-0.32	1.00	0.05	1.00	-0.60	1.00	-0.33	1.00	-0.43	1.00	-0.28	1.00	0.15	1.00
g. *Halomonas*	0.00	0.00	0.00	0.00	0.00	0.00	-0.17	1.00	-0.22	1.00	-0.05	1.00	-0.63	1.00	-0.52	1.00	0.11	1.00	-0.54	1.00	-0.09	1.00	-0.24	1.00	0.08	1.00	-0.20	1.00	-0.28	1.00
g. *Actinobacillus*	0.00	0.00	0.00	0.00	0.00	0.00	-0.62	1.00	-0.74	1.00	-0.11	1.00	-0.17	1.00	0.06	1.00	0.24	1.00	1.01	1.00	0.56	1.00	0.21	1.00	1.18	1.00	0.32	1.00	-0.86	1.00
g. *Aggregatibacter*	0.01	0.00	0.00	0.00	0.00	0.00	-0.32	1.00	-0.84	1.00	-0.52	1.00	-0.34	1.00	-0.64	1.00	-0.30	1.00	0.54	1.00	0.56	1.00	0.34	1.00	0.88	1.00	0.86	1.00	-0.03	1.00
g. *Gallibacterium*	0.16	0.02	0.00	0.02	0.00	0.00	-0.16	1.00	-5.50	0.03	-5.34	0.93	-3.68	0.90	-4.49	0.46	-0.81	1.00	5.96	0.96	9.48	0.00	4.95	1.00	9.64	0.00	10.29	0.00	0.65	1.00

	g. *Haemophilus*	0.01	0.00	0.00	0.00	0.00	0.00	-6.83	1.00	-21.77	1.00	-14.93	0.53	4.09	1.00	2.07	1.00	-2.02	1.00	2.92	1.00	-8.00	1.00	-20.92	1.00	-1.17	1.00	-5.98	1.00	-4.81	1.00
	g. *Mannheimia*	0.00	0.00	0.00	0.00	0.00	0.00	0.24	1.00	-0.35	1.00	-0.59	1.00	0.31	1.00	0.14	1.00	-0.17	1.00	-0.11	1.00	-0.18	1.00	-0.60	1.00	-0.42	1.00	-0.01	1.00	0.41	1.00
	f. Pasteurellaceae	0.00	0.00	0.00	0.01	0.03	0.01	0.79	1.00	-0.65	1.00	-1.44	1.00	1.04	1.00	2.37	1.00	1.33	1.00	-0.70	1.00	-0.95	1.00	-3.71	1.00	-1.74	1.00	-2.28	1.00	-0.54	1.00
	g. *Acinetobacter*	0.24	0.01	0.01	0.12	0.06	0.02	-3.34	0.01	-3.74	0.00	-0.40	1.00	-2.10	0.55	-2.56	0.15	-0.46	1.00	1.31	1.00	0.07	1.00	0.13	1.00	3.41	0.01	0.53	1.00	-2.88	0.74
	g. *Alkanindiges*	0.00	0.00	0.00	0.00	0.00	0.00	-2.64	1.00	-2.70	1.00	-0.06	1.00	-0.17	1.00	0.06	1.00	0.24	1.00	2.84	1.00	0.38	1.00	0.08	1.00	3.02	1.00	0.14	1.00	-2.88	1.00
	g. *Enhydrobacter*	0.00	0.00	0.00	0.00	0.00	0.00	-0.17	1.00	-0.22	1.00	-0.05	1.00	0.31	1.00	0.14	1.00	-0.17	1.00	-0.13	1.00	-0.61	1.00	-0.49	1.00	-0.43	1.00	-0.44	1.00	-0.01	1.00
	f. Moraxellaceae	0.01	0.00	0.00	0.00	0.00	0.00	-6.36	1.00	-5.81	1.00	0.55	1.00	-2.79	1.00	-1.95	1.00	0.84	1.00	3.68	1.00	0.10	1.00	-0.18	1.00	6.46	1.00	-0.73	1.00	-7.20	1.00
	g. *Psychrobacter*	0.00	0.00	0.00	0.00	0.00	0.00	-0.17	1.00	-0.22	1.00	-0.05	1.00	-0.72	1.00	-0.48	1.00	0.24	1.00	-0.54	1.00	0.00	1.00	-0.28	1.00	0.18	1.00	-0.24	1.00	-0.41	1.00
	f. Pseudomonadaceae	0.01	0.00	0.00	0.02	0.00	0.01	-1.28	1.00	-2.56	0.55	-1.28	1.00	-4.64	0.20	-2.16	0.92	2.49	1.00	-1.92	1.00	1.44	1.00	-2.32	1.00	2.73	0.72	-1.04	1.00	-3.77	1.00
	g. *Pseudomonas*	0.04	0.01	0.00	0.03	0.00	0.01	-3.81	0.02	-5.12	0.00	-1.31	1.00	-2.92	0.55	-1.35	1.00	1.57	1.00	2.12	1.00	1.23	1.00	-1.65	1.00	5.04	0.00	-0.34	1.00	-5.38	0.17
	g. *Methylophaga*	0.00	0.00	0.00	0.00	0.00	0.00	-3.15	1.00	-3.27	1.00	-0.11	1.00	-0.87	1.00	-0.89	1.00	-0.01	1.00	2.07	1.00	-0.21	1.00	-0.31	1.00	2.94	1.00	-0.20	1.00	-3.14	1.00
	f. Sinobacteraceae	0.00	0.00	0.00	0.00	0.00	0.00	-0.62	1.00	-0.73	1.00	-0.11	1.00	-0.08	1.00	0.76	1.00	0.84	1.00	0.91	1.00	0.37	1.00	-0.58	1.00	0.99	1.00	-0.47	1.00	-1.46	1.00
	g. *Luteimonas*	0.01	0.00	0.00	0.01	0.00	0.00	-3.25	1.00	-4.06	1.00	-0.81	1.00	-2.27	1.00	-2.36	1.00	-0.10	1.00	0.22	1.00	-0.77	1.00	-1.48	1.00	2.49	1.00	-0.67	1.00	-3.16	1.00
	f. Xanthomonadaceae	0.02	0.02	0.00	0.02	0.01	0.01	-1.73	0.67	-3.21	0.05	-1.48	1.00	0.21	1.00	-0.54	1.00	-0.75	1.00	1.95	1.00	0.01	1.00	-0.72	1.00	1.74	0.73	0.76	1.00	-0.98	1.00
	g. *Pseudoxanthomonas*	0.00	0.00	0.00	0.00	0.00	0.00	-0.80	1.00	-0.92	1.00	-0.12	1.00	-0.17	1.00	0.06	1.00	0.24	1.00	1.00	1.00	0.38	1.00	0.02	1.00	1.18	1.00	0.14	1.00	-1.04	1.00
	g. *Stenotrophomonas*	0.12	0.00	0.00	0.02	0.00	0.00	-5.82	1.00	-5.73	1.00	0.10	1.00	-0.10	1.00	-1.28	1.00	-1.18	1.00	4.16	1.00	-1.56	1.00	-0.28	1.00	4.26	1.00	-0.38	1.00	-4.64	1.00
Spirochaetes		0.67	0.07	0.12	0.61	0.26	0.13	-5.12	0.01	-5.98	0.00	-0.86	1.00	3.32	0.28	1.36	0.99	-1.97	0.84	1.84	0.99	-6.60	0.00	-5.50	0.04	-1.49	0.83	-4.63	0.11	-3.15	0.97
	g. *Sphaerochaeta*	0.00	0.00	0.00	0.00	0.00	0.00	-0.98	1.00	-1.75	1.00	-0.76	1.00	0.31	1.00	0.03	1.00	-0.29	1.00	1.51	1.00	0.22	1.00	-0.26	1.00	1.20	1.00	0.50	1.00	-0.70	1.00
	g. *Treponema*	0.67	0.07	0.12	0.61	0.26	0.13	-4.70	0.05	-5.50	0.01	-0.81	1.00	3.32	0.52	1.22	1.00	-2.10	1.00	0.34	1.00	-7.67	0.00	-6.38	0.18	-2.98	0.41	-5.57	1.00	-2.60	1.00
Synergistetes		0.54	0.05	0.09	0.23	0.06	0.02	-4.71	0.00	-3.08	0.07	1.63	1.00	-2.40	0.39	-3.32	0.06	-0.91	0.96	2.96	0.99	0.65	1.00	3.20	0.59	5.36	0.00	1.57	1.00	-3.79	0.97
	f. Dethiosulfovibrionaceae	0.00	0.00	0.00	0.00	0.00	0.00	-0.17	1.00	-0.22	1.00	-0.05	1.00	-0.84	1.00	-0.54	1.00	0.30	1.00	-0.77	1.00	-0.10	1.00	-0.45	1.00	0.08	1.00	-0.40	1.00	-0.48	1.00
	g. *Pyramidobacter*	0.04	0.05	0.09	0.11	0.06	0.02	2.15	0.90	3.74	0.12	1.59	1.00	-0.64	1.00	-0.30	1.00	0.34	1.00	-1.83	1.00	0.96	1.00	2.21	1.00	-1.19	1.00	0.62	1.00	1.81	1.00
	f. Synergistaceae	0.48	0.00	0.00	0.11	0.00	0.00	-8.83	0.00	-9.41	0.00	-0.59	1.00	-2.74	1.00	-4.30	0.57	-1.56	1.00	5.66	1.00	-0.42	1.00	0.55	1.00	8.40	0.00	1.14	1.00	-7.27	0.45
	g. *Synergistes*	0.01	0.00	0.00	0.00	0.00	0.00	-5.05	1.00	-5.05	1.00	0.00	1.00	-0.83	1.00	-0.53	1.00	0.30	1.00	4.38	1.00	0.16	1.00	-0.15	1.00	5.21	1.00	-0.14	1.00	-5.35	1.00
Tenericutes		1.21	0.82	1.79	0.27	0.18	2.76	1.90	0.21	0.05	1.00	-1.85	1.00	-0.66	0.98	3.56	0.03	4.22	0.01	1.60	0.99	4.15	0.01	-1.92	0.83	2.25	0.81	-0.07	1.00	-2.32	0.97
	g. *Mycoplasma*	0.00	0.00	0.00	0.00	0.00	0.00	-1.02	1.00	-1.08	1.00	-0.06	1.00	-0.49	1.00	-1.64	1.00	-1.15	1.00	-0.70	1.00	-1.24	1.00	-0.15	1.00	-0.22	1.00	-0.09	1.00	0.13	1.00
	o. RF39	1.19	0.82	1.79	0.25	0.17	2.76	1.65	0.90	0.21	1.00	-1.44	1.00	0.26	1.00	3.86	0.06	3.60	0.62	2.36	1.00	3.75	0.17	-1.29	1.00	2.10	0.66	0.15	1.00	-1.94	1.00
	o. ML615J-28	0.02	0.00	0.00	0.02	0.00	0.00	-6.62	1.00	-6.72	0.88	-0.10	1.00	-2.87	1.00	-1.90	1.00	0.97	1.00	3.41	1.00	-0.34	1.00	-1.41	1.00	6.28	1.00	-1.31	1.00	-7.59	1.00
TM7	f. Rs-045	0.00	0.00	0.00	0.00	0.00	0.00	1.58	1.00	0.26	1.00	-1.32	1.00	1.35	1.00	0.44	1.00	-0.91	1.00	0.13	1.00	0.36	1.00	-0.05	1.00	-1.22	1.00	1.27	1.00	2.49	1.00
Verrucomicrobia		0.17	0.00	0.00	0.21	0.02	0.00	-4.71	0.04	-3.73	0.12	0.98	1.00	0.05	0.98	-4.94	0.06	-4.98	0.08	0.24	0.99	-4.51	0.06	1.45	0.93	0.20	0.90	0.47	1.00	0.27	0.97
	f. [Cerasicoccaceae]	0.00	0.00	0.00	0.00	0.00	0.00	-0.95	1.00	-1.81	1.00	-0.86	1.00	0.11	1.00	-0.46	1.00	-0.57	1.00	1.37	1.00	0.31	1.00	0.02	1.00	1.26	1.00	0.88	1.00	-0.37	1.00
	g. *Opitutus*	0.00	0.00	0.00	0.00	0.00	0.00	-0.62	1.00	-0.73	1.00	-0.11	1.00	-0.17	1.00	0.06	1.00	0.24	1.00	0.95	1.00	0.50	1.00	0.15	1.00	1.12	1.00	0.26	1.00	-0.86	1.00
	f. RFP12	0.15	0.00	0.00	0.20	0.01	0.00	-9.84	0.90	-6.46	1.00	3.38	1.00	-1.90	1.00	-7.02	1.00	-5.11	1.00	-1.57	1.00	-9.51	0.89	-1.02	1.00	0.33	1.00	-4.39	1.00	-4.73	1.00
	f. WCHB1-25	0.00	0.00	0.00	0.00	0.01	0.00	-0.17	1.00	-0.22	1.00	-0.05	1.00	0.56	1.00	-1.65	1.00	-2.21	1.00	-2.19	1.00	-2.92	1.00	-0.76	1.00	-2.74	1.00	-0.71	1.00	2.03	1.00
	g. *Akkermansia*	0.01	0.00	0.00	0.00	0.00	0.00	-24.87	1.00	4.83	1.00	29.70	0.00	16.58	1.00	-12.95	1.00	-29.52	0.00	22.57	1.00	-18.88	1.00	40.35	1.00	6.00	1.00	10.65	1.00	4.65	1.00
	g. *Luteolibacter*	0.01	0.00	0.00	0.00	0.00	0.00	-4.02	1.00	-4.07	1.00	-0.05	1.00	-0.76	1.00	-0.48	1.00	0.28	1.00	3.36	1.00	0.10	1.00	-0.24	1.00	4.12	1.00	-0.19	1.00	-4.31	1.00
WPS-2		0.00	0.00	0.00	0.00	0.00	0.00	-0.78	1.00	-0.90	1.00	-0.12	1.00	-0.17	1.00	0.06	1.00	0.24	1.00	0.94	1.00	0.33	1.00	-0.03	1.00	1.11	1.00	0.09	1.00	-1.02	1.00
WWE1	g. *Candidatus Cloacamonas*	0.00	0.00	0.00	0.00	0.00	0.00	0.45	1.00	0.52	1.00	0.08	1.00	0.47	1.00	0.16	1.00	-0.31	1.00	0.43	1.00	0.41	1.00	0.80	1.00	-0.04	1.00	0.72	1.00	0.76	1.00

Supplementary Table 2. Faecal taxa

								Early-weaned						Late-weaned																	
		Early-weaned			Late-weaned			Wk 5 vs Wk 7		Wk 5 vs Wk 9		Wk 7 vs Wk 9		Wk 5 vs Wk 7		Wk 5 vs Wk 9		Wk 7 vs Wk 9		Early Wk 5 vs Late Wk 5		Early Wk 7 vs Late Wk 7		Early Wk 9 vs Late Wk 9		Early Wk 5 vs Late Wk 7		Early Wk 7 vs Late Wk 9		(Early Wk 7 - Wk 5) vs (Late Wk 9 - Wk 7)	
**Phylum**	**Taxa**	**Wk 5**	**Wk7**	**Wk9**	**Wk 5**	**Wk7**	**Wk9**	**Log2 FC**	**p-value**	**Log2 FC**	**p-value**	**Log2 FC**	**p-value**	**Log2 FC**	**p-value**	**Log2 FC**	**p-value**	**Log2 FC**	**p-value**	**Log2 FC**	**p-value**	**Log2 FC**	**p-value**	**Log2 FC**	**p-value**	**Log2 FC**	**p-value**	**Log2 FC**	**p-value**	**Log2 FC**	**p-value**
Acidobacteria	o. Solibacterales	0.00	0.00	0.00	0.00	0.00	0.00	-0.056	1.000	-0.050	1.00	0.006	1.000	-1.253	1.000	-0.581	1.000	0.672	1.000	-0.702	1.000	0.495	1.000	-0.171	1.000	0.551	1.000	-0.177	1.000	-0.728	1.000
Actinobacteria		5.67	3.33	2.30	6.00	2.92	2.93	-1.165	0.128	-1.240	0.14	-0.076	0.991	-0.443	0.964	-0.712	0.756	-0.269	0.994	0.266	0.991	-0.455	0.996	-0.262	0.991	0.710	0.653	-0.186	1.000	-0.896	0.819
	g. *Actinomyces*	0.03	0.00	0.00	0.01	0.00	0.00	-4.665	0.216	-5.863	0.06	-1.198	1.000	-3.500	1.000	-3.674	0.484	-0.174	1.000	2.344	1.000	1.178	1.000	0.155	1.000	5.844	0.429	1.352	1.000	-4.491	1.000
	g. *Mobiluncus*	0.00	0.00	0.00	0.00	0.00	0.00	-0.054	1.000	1.118	1.00	1.172	1.000	-0.487	1.000	1.312	1.000	1.800	1.000	-0.549	1.000	-0.116	1.000	-0.744	1.000	-0.062	1.000	-1.916	1.000	-1.854	1.000
	g. *Trueperella*	0.00	0.00	0.00	0.00	0.00	0.00	-0.056	1.000	-0.050	1.00	0.006	1.000	-1.042	1.000	-0.639	1.000	0.404	1.000	-1.305	1.000	-0.319	1.000	-0.716	1.000	-0.263	1.000	-0.722	1.000	-0.460	1.000
	f. Cellulomonadaceae	0.00	0.00	0.00	0.00	0.00	0.00	-0.716	1.000	-0.725	1.00	-0.009	1.000	-0.681	1.000	-0.052	1.000	0.629	1.000	0.672	1.000	0.637	1.000	-0.001	1.000	1.353	1.000	0.008	1.000	-1.346	1.000
	g. *Corynebacterium*	0.02	0.00	0.00	0.00	0.02	0.00	-3.509	0.395	-3.340	0.40	0.169	1.000	1.216	1.000	-1.961	1.000	-3.177	0.807	0.671	1.000	-4.054	0.349	-0.708	1.000	-0.545	1.000	-0.878	1.000	-0.332	1.000
	g. *Dietzia*	0.00	0.00	0.00	0.00	0.00	0.00	-0.056	1.000	-0.050	1.00	0.006	1.000	-0.898	1.000	0.159	1.000	1.057	1.000	-0.817	1.000	0.025	1.000	-1.025	1.000	0.081	1.000	-1.032	1.000	-1.113	1.000
	f. Geodermatophilaceae	0.00	0.00	0.00	0.00	0.00	0.00	-0.056	1.000	-0.050	1.00	0.006	1.000	-0.504	1.000	-0.463	1.000	0.041	1.000	-0.830	1.000	-0.382	1.000	-0.417	1.000	-0.326	1.000	-0.423	1.000	-0.097	1.000
	g. *Frigoribacterium*	0.00	0.00	0.00	0.00	0.00	0.00	-0.056	1.000	-0.050	1.00	0.006	1.000	-0.625	1.000	0.555	1.000	1.180	1.000	-0.193	1.000	0.376	1.000	-0.797	1.000	0.432	1.000	-0.804	1.000	-1.236	1.000
	g. *Leucobacter*	0.00	0.00	0.00	0.00	0.01	0.00	-0.056	1.000	-0.050	1.00	0.006	1.000	3.261	1.000	0.070	1.000	-3.191	1.000	-0.058	1.000	-3.375	1.000	-0.178	1.000	-3.319	1.000	-0.184	1.000	3.135	1.000
	g. *Rathayibacter*	0.00	0.00	0.00	0.00	0.00	0.00	-0.056	1.000	-0.050	1.00	0.006	1.000	-0.274	1.000	-0.293	1.000	-0.019	1.000	-0.456	1.000	-0.238	1.000	-0.213	1.000	-0.182	1.000	-0.219	1.000	-0.037	1.000
	f. Micrococcaceae	0.00	0.00	0.00	0.00	0.00	0.00	-1.321	1.000	-1.295	1.00	0.026	1.000	-0.172	1.000	-0.047	1.000	0.125	1.000	1.046	1.000	-0.103	1.000	-0.202	1.000	1.217	1.000	-0.228	1.000	-1.445	1.000
	g. *Rhodococcus*	0.00	0.00	0.00	0.00	0.00	0.00	-0.541	1.000	-0.513	1.00	0.028	1.000	-0.681	1.000	-0.052	1.000	0.629	1.000	0.581	1.000	0.721	1.000	0.120	1.000	1.262	1.000	0.092	1.000	-1.170	1.000
	g. *Saccharomonospora*	0.00	0.00	0.00	0.00	0.00	0.00	-0.056	1.000	-0.050	1.00	0.006	1.000	-1.182	1.000	-0.522	1.000	0.660	1.000	-0.812	1.000	0.314	1.000	-0.339	1.000	0.370	1.000	-0.345	1.000	-0.716	1.000
	g. *Bifidobacterium*	3.63	1.68	0.06	4.61	0.73	0.16	-5.701	0.000	-6.080	0.00	-0.380	1.000	-3.111	0.331	-4.646	0.000	-1.536	1.000	1.483	1.000	-1.108	1.000	0.048	1.000	4.593	0.006	0.428	1.000	-4.165	1.000
	g. *Adlercreutzia*	0.02	0.00	0.00	0.00	0.00	0.00	-0.823	1.000	-2.139	1.00	-1.317	1.000	0.509	1.000	1.478	1.000	0.969	1.000	3.926	1.000	2.595	1.000	0.309	1.000	3.417	1.000	1.626	1.000	-1.792	1.000
	g. *Atopobium*	0.00	0.00	0.00	0.00	0.00	0.00	-0.056	1.000	-0.050	1.00	0.006	1.000	-0.625	1.000	0.555	1.000	1.180	1.000	-0.193	1.000	0.376	1.000	-0.797	1.000	0.432	1.000	-0.804	1.000	-1.236	1.000
	g. *Collinsella*	0.90	0.07	0.02	1.01	0.30	0.04	-5.312	0.000	-4.910	0.00	0.402	1.000	-1.772	0.970	-6.937	0.000	-5.165	0.000	-0.329	1.000	-3.869	0.005	1.698	1.000	1.443	1.000	1.296	1.000	-0.147	1.000
	g. *Eggerthella*	0.01	0.00	0.00	0.00	0.00	0.00	-3.809	1.000	-4.519	1.00	-0.710	1.000	-0.651	1.000	0.037	1.000	0.688	1.000	3.381	1.000	0.222	1.000	-1.175	1.000	4.032	1.000	-0.465	1.000	-4.497	1.000
	f. Coriobacteriaceae	1.05	1.57	2.20	0.35	1.86	2.71	1.648	0.261	2.451	0.01	0.803	1.000	2.579	0.264	3.939	0.000	1.359	0.910	1.367	1.000	0.436	1.000	-0.120	1.000	-1.212	1.000	-0.923	1.000	0.289	1.000
	g. Slackia	0.00	0.00	0.00	0.00	0.00	0.00	0.330	1.000	2.224	1.00	1.894	1.000	-0.086	1.000	2.915	1.000	3.001	1.000	0.570	1.000	0.986	1.000	-0.121	1.000	0.656	1.000	-2.015	1.000	-2.671	1.000
Bacteroidetes		40.14	44.69	42.94	42.24	50.07	44.61	0.255	0.537	0.186	0.93	-0.070	0.991	0.343	0.964	0.226	0.983	-0.118	0.994	-0.077	0.991	-0.165	0.996	-0.117	0.991	-0.420	0.653	-0.047	1.000	0.373	0.819
	f. [Barnesiellaceae]	0.50	0.10	0.10	0.13	0.26	0.17	-0.760	1.000	-0.954	1.00	-0.194	1.000	1.495	1.000	1.443	0.699	-0.052	1.000	1.884	1.000	-0.371	1.000	-0.513	1.000	0.389	1.000	-0.319	1.000	-0.709	1.000
	g. *Butyricimonas*	0.26	0.35	0.15	0.28	0.11	0.23	0.081	1.000	-1.626	1.00	-1.707	1.000	0.538	1.000	0.116	1.000	-0.422	1.000	1.660	1.000	1.203	1.000	-0.082	1.000	1.122	1.000	1.625	1.000	0.503	1.000
	g. *Odoribacter*	0.57	0.55	0.37	0.38	1.01	0.52	0.403	1.000	0.044	1.00	-0.359	1.000	2.385	0.264	1.855	0.054	-0.530	1.000	1.577	1.000	-0.404	1.000	-0.233	1.000	-0.808	1.000	0.126	1.000	0.933	1.000
	g. *[Prevotella]*	5.49	5.31	3.37	3.78	6.62	4.84	1.671	0.182	1.155	0.63	-0.517	1.000	0.942	1.000	1.300	0.294	0.358	1.000	-0.155	1.000	0.575	1.000	-0.300	1.000	-1.096	1.000	0.217	1.000	1.313	1.000
	g. *CF231*	0.00	0.00	0.00	0.00	0.00	0.00	-0.056	1.000	-0.050	1.00	0.006	1.000	-1.182	1.000	-0.522	1.000	0.660	1.000	-0.812	1.000	0.314	1.000	-0.339	1.000	0.370	1.000	-0.345	1.000	-0.716	1.000
	f. [Paraprevotellaceae]	0.07	0.01	0.01	0.01	0.00	0.01	-2.213	1.000	-1.944	1.00	0.269	1.000	-0.586	1.000	0.771	1.000	1.357	1.000	2.034	1.000	0.407	1.000	-0.681	1.000	2.620	1.000	-0.950	1.000	-3.570	1.000
	g. *YRC22*	0.00	0.11	0.05	0.00	0.00	0.01	0.235	1.000	0.525	1.00	0.289	1.000	-0.892	1.000	0.966	1.000	1.857	1.000	0.545	1.000	1.672	1.000	0.104	1.000	1.436	1.000	-0.186	1.000	-1.622	1.000
	g. *Bacteroides*	27.17	18.58	18.80	30.75	33.87	21.62	-0.314	1.000	-0.185	1.00	0.129	1.000	-0.415	1.000	-0.332	1.000	0.082	1.000	-0.360	1.000	-0.259	1.000	-0.212	1.000	0.055	1.000	-0.341	1.000	-0.396	1.000
	f. Bacteroidaceae	0.00	0.00	0.00	0.00	0.00	0.00	-0.593	1.000	-0.612	1.00	-0.020	1.000	-0.658	1.000	0.482	1.000	1.140	1.000	0.412	1.000	0.477	1.000	-0.682	1.000	1.070	1.000	-0.662	1.000	-1.732	1.000
	f.BS11	0.00	0.00	0.00	0.00	0.00	0.00	-0.199	1.000	0.325	1.00	0.524	1.000	-0.681	1.000	-0.052	1.000	0.629	1.000	0.207	1.000	0.689	1.000	0.584	1.000	0.888	1.000	0.060	1.000	-0.828	1.000
	o. Bacteroidales	0.06	1.47	1.52	0.05	0.57	1.09	4.086	0.000	4.270	0.00	0.184	1.000	1.653	0.970	2.793	0.011	1.140	1.000	-0.096	1.000	2.338	0.147	1.382	1.000	-1.748	1.000	1.198	1.000	2.946	1.000
	g. *Paludibacter*	0.00	0.00	0.00	0.00	0.00	0.00	-0.056	1.000	-0.050	1.00	0.006	1.000	-0.850	1.000	0.362	1.000	1.212	1.000	-0.494	1.000	0.300	1.000	-0.906	1.000	0.356	1.000	-0.912	1.000	-1.268	1.000
	g. *Parabacteroides*	3.60	3.05	2.25	4.20	6.15	3.01	0.519	1.000	0.167	1.00	-0.352	1.000	0.798	1.000	0.489	1.000	-0.309	1.000	-0.029	1.000	-0.308	1.000	-0.351	1.000	-0.827	1.000	0.001	1.000	0.828	1.000
	f. Prevotellaceae	0.00	0.00	0.00	0.00	0.00	0.00	3.339	1.000	1.808	1.00	-1.531	1.000	-1.252	1.000	0.067	1.000	1.319	1.000	-0.533	1.000	4.058	1.000	1.208	1.000	0.719	1.000	2.739	1.000	2.020	1.000
	g. *Prevotella*	0.89	9.78	4.93	0.59	0.39	5.77	4.474	0.000	3.104	0.00	-1.370	1.000	-0.337	1.000	3.981	0.000	4.319	0.000	1.029	1.000	5.840	0.000	0.152	1.000	1.366	1.000	1.522	1.000	0.155	1.000
	f. RF16	0.00	0.00	0.00	0.00	0.00	0.00	0.354	1.000	0.355	1.00	0.001	1.000	0.076	1.000	1.364	1.000	1.288	1.000	0.506	1.000	0.784	1.000	-0.503	1.000	0.430	1.000	-0.504	1.000	-0.934	1.000
	f. Rikenellaceae	0.95	0.64	0.61	1.16	0.74	0.45	-0.065	1.000	-0.029	1.00	0.036	1.000	0.001	1.000	-0.379	1.000	-0.380	1.000	0.018	1.000	-0.048	1.000	0.367	1.000	0.017	1.000	0.332	1.000	0.315	1.000
	f. S24-7	0.57	4.74	10.79	0.90	0.34	6.89	5.644	0.000	6.873	0.00	1.229	1.000	0.284	1.000	7.502	0.000	7.218	0.000	1.011	1.000	6.371	0.000	0.382	1.000	0.727	1.000	-0.848	1.000	-1.575	1.000
	Wautersiella	0.00	0.00	0.00	0.00	0.00	0.00	-0.056	1.000	-0.050	1.00	0.006	1.000	-0.064	1.000	0.042	1.000	0.106	1.000	-0.058	1.000	-0.050	1.000	-0.150	1.000	0.006	1.000	-0.156	1.000	-0.162	1.000
Chlamydiae	f. Parachlamydiaceae	0.00	0.00	0.00	0.00	0.00	0.00	-0.606	1.000	-0.610	1.00	-0.003	1.000	-0.681	1.000	-0.052	1.000	0.629	1.000	0.685	1.000	0.759	1.000	0.127	1.000	1.366	1.000	0.130	1.000	-1.236	1.000
Cyanobacteria	o. YS2	0.19	0.65	0.77	0.15	0.43	0.39	1.840	0.197	2.031	0.06	0.191	1.000	2.037	0.588	2.444	0.011	0.407	1.000	0.903	1.000	0.706	1.000	0.490	1.000	-1.134	1.000	0.299	1.000	1.433	1.000
Cyanobacteria	o. Streptophyta	0.00	0.00	0.00	0.00	0.00	0.00	-0.553	1.000	-0.525	1.00	0.028	1.000	-0.038	1.000	0.665	1.000	0.702	1.000	0.451	1.000	-0.065	1.000	-0.739	1.000	0.488	1.000	-0.767	1.000	-1.256	1.000
Elusimicrobia	f. Elusimicrobiaceae	0.02	0.02	0.02	0.02	0.00	0.01	-3.933	1.000	-2.653	1.00	1.281	1.000	-0.776	1.000	1.017	1.000	1.793	1.000	2.771	1.000	-0.386	1.000	-0.899	1.000	3.547	1.000	-2.179	1.000	-5.727	1.000
Fibrobacteres	g. *Fibrobacter*	0.00	0.00	0.00	0.00	0.00	0.00	-0.100	1.000	0.456	1.00	0.557	1.000	-0.681	1.000	-0.052	1.000	0.629	1.000	0.069	1.000	0.650	1.000	0.577	1.000	0.750	1.000	0.020	1.000	-0.730	1.000

Firmicutes		46.38	38.77	44.40	45.28	40.34	38.00	-0.283	0.391	-0.103	0.93	0.180	0.991	-0.054	0.996	-0.111	0.983	-0.057	0.994	0.160	0.991	-0.070	0.996	0.167	0.991	0.214	0.706	-0.013	1.000	-0.227	0.819
	g. *Anaerobacillus*	0.00	0.00	0.00	0.00	0.00	0.00	-0.056	1.000	-0.050	1.00	0.006	1.000	-0.177	1.000	-0.047	1.000	0.129	1.000	-0.300	1.000	-0.179	1.000	-0.302	1.000	-0.123	1.000	-0.308	1.000	-0.185	1.000
	g. *Bacillus*	0.00	0.00	0.00	0.00	0.00	0.00	0.488	1.000	0.508	1.00	0.020	1.000	0.295	1.000	1.464	1.000	1.169	1.000	0.167	1.000	0.360	1.000	-0.789	1.000	-0.128	1.000	-0.809	1.000	-0.682	1.000
	f. Bacillaceae	0.00	0.00	0.00	0.00	0.00	0.00	-0.716	1.000	-0.725	1.00	-0.009	1.000	-0.681	1.000	-0.052	1.000	0.629	1.000	0.672	1.000	0.637	1.000	-0.001	1.000	1.353	1.000	0.008	1.000	-1.346	1.000
	o. Bacillales	0.00	0.00	0.00	0.00	0.00	0.00	0.326	1.000	0.363	1.00	0.036	1.000	-0.681	1.000	-0.052	1.000	0.629	1.000	0.464	1.000	1.471	1.000	0.878	1.000	1.145	1.000	0.842	1.000	-0.303	1.000
	f. Paenibacillaceae	0.00	0.00	0.00	0.00	0.00	0.00	-0.056	1.000	-0.050	1.00	0.006	1.000	-1.276	1.000	-0.602	1.000	0.673	1.000	-0.815	1.000	0.405	1.000	-0.262	1.000	0.461	1.000	-0.268	1.000	-0.729	1.000
	g. *Lysinibacillus*	0.00	0.00	0.00	0.00	0.00	0.00	0.342	1.000	0.354	1.00	0.012	1.000	-0.681	1.000	-0.052	1.000	0.629	1.000	0.461	1.000	1.483	1.000	0.867	1.000	1.142	1.000	0.854	1.000	-0.287	1.000
	g. *Macrococcus*	0.00	0.00	0.00	0.00	0.00	0.00	-1.238	1.000	-1.179	1.00	0.059	1.000	-0.350	1.000	-0.290	1.000	0.060	1.000	0.250	1.000	-0.638	1.000	-0.639	1.000	0.600	1.000	-0.698	1.000	-1.298	1.000
	g. *Staphylococcus*	0.00	0.00	0.00	0.00	0.00	0.00	-0.558	1.000	0.091	1.00	0.648	1.000	-0.681	1.000	-0.052	1.000	0.629	1.000	1.040	1.000	1.163	1.000	1.182	1.000	1.721	1.000	0.534	1.000	-1.187	1.000
	g. *Aerococcus*	0.00	0.00	0.00	0.00	0.00	0.00	-0.056	1.000	-0.050	1.00	0.006	1.000	-0.711	1.000	0.446	1.000	1.158	1.000	-0.367	1.000	0.288	1.000	-0.863	1.000	0.344	1.000	-0.869	1.000	-1.214	1.000
	g. *Facklamia*	0.00	0.00	0.00	0.00	0.00	0.00	-0.034	1.000	0.440	1.00	0.474	1.000	-0.470	1.000	0.129	1.000	0.599	1.000	-0.980	1.000	-0.545	1.000	-0.670	1.000	-0.511	1.000	-1.144	1.000	-0.634	1.000
	f. Aerococcaceae	0.00	0.00	0.00	0.00	0.00	0.00	-0.056	1.000	-0.050	1.00	0.006	1.000	-1.276	1.000	-0.602	1.000	0.673	1.000	-0.815	1.000	0.405	1.000	-0.262	1.000	0.461	1.000	-0.268	1.000	-0.729	1.000
	g. *Trichococcus*	0.00	0.00	0.00	0.00	0.00	0.00	-0.066	1.000	0.473	1.00	0.539	1.000	-0.819	1.000	0.391	1.000	1.210	1.000	-0.028	1.000	0.725	1.000	0.055	1.000	0.791	1.000	-0.485	1.000	-1.276	1.000
	g. *Enterococcus*	0.05	0.00	0.00	0.02	0.11	0.00	-6.213	0.058	-7.567	0.01	-1.354	1.000	-0.313	1.000	-5.310	0.081	-4.997	0.304	0.837	1.000	-5.062	0.349	-1.420	1.000	1.151	1.000	-0.066	1.000	-1.216	1.000
	g. *Lactobacillus*	1.46	0.09	0.01	0.60	1.76	0.02	-5.768	0.000	-5.645	0.00	0.123	1.000	1.091	1.000	-3.305	0.011	-4.396	0.001	0.505	1.000	-6.354	0.000	-1.835	1.000	-0.586	1.000	-1.958	1.000	-1.372	1.000
	f. Lactobacillaceae	0.00	0.00	0.00	0.00	0.00	0.00	-0.056	1.000	-0.050	1.00	0.006	1.000	0.596	1.000	0.674	1.000	0.078	1.000	-0.360	1.000	-1.012	1.000	-1.084	1.000	-0.956	1.000	-1.090	1.000	-0.134	1.000
	g. *Leuconostoc*	0.03	0.00	0.00	0.01	0.01	0.00	-7.028	0.001	-7.005	0.00	0.022	1.000	-0.751	1.000	-5.548	0.011	-4.796	0.091	1.388	1.000	-4.888	0.127	-0.070	1.000	2.139	1.000	-0.092	1.000	-2.231	1.000
	o. Lactobacillales	0.00	0.00	0.00	0.00	0.00	0.00	-2.063	1.000	-2.641	1.00	-0.578	1.000	-1.334	1.000	-0.553	1.000	0.782	1.000	0.849	1.000	0.120	1.000	-1.239	1.000	2.183	1.000	-0.662	1.000	-2.845	1.000
	g. *Lactococcus*	0.01	0.00	0.00	0.02	0.04	0.00	-5.286	0.058	-6.702	0.01	-1.417	1.000	-1.987	1.000	-5.839	0.006	-3.852	0.358	-0.738	1.000	-4.036	0.492	-1.601	1.000	1.249	1.000	-0.185	1.000	-1.434	1.000
	g. *Streptococcus*	5.97	0.72	0.01	3.37	0.95	0.09	-7.547	0.000	-10.428	0.00	-2.881	1.000	-2.380	0.588	-6.642	0.000	-4.263	0.000	1.378	1.000	-3.789	0.005	-2.407	1.000	3.758	0.014	0.474	1.000	-3.284	1.000
	g. *Turicibacter*	0.00	0.00	0.00	0.00	0.00	0.00	-1.269	1.000	-1.209	1.00	0.060	1.000	-0.169	1.000	-0.102	1.000	0.067	1.000	1.175	1.000	0.074	1.000	0.068	1.000	1.343	1.000	0.008	1.000	-1.336	1.000
	g. *Anaerovorax*	0.00	0.00	0.00	0.00	0.00	0.00	0.232	1.000	0.399	1.00	0.167	1.000	-0.470	1.000	1.355	1.000	1.825	1.000	0.949	1.000	1.651	1.000	-0.007	1.000	1.419	1.000	-0.174	1.000	-1.593	1.000
	g. *Mogibacterium*	0.14	0.20	0.23	0.02	0.11	0.20	1.756	0.206	1.604	0.22	-0.152	1.000	1.848	0.643	3.444	0.000	1.595	0.504	2.379	1.000	2.287	0.086	0.539	1.000	0.531	1.000	0.692	1.000	0.160	1.000
	f. [Mogibacteriaceae]	0.10	0.28	0.31	0.06	0.25	0.34	2.000	0.057	2.251	0.01	0.251	1.000	1.908	0.565	2.787	0.000	0.880	1.000	0.687	1.000	0.779	1.000	0.150	1.000	-1.221	1.000	-0.101	1.000	1.120	1.000
	g. *Anaerococcus*	0.00	0.00	0.00	0.00	0.00	0.00	-0.056	1.000	-0.050	1.00	0.006	1.000	-0.721	1.000	0.492	1.000	1.213	1.000	-0.257	1.000	0.408	1.000	-0.799	1.000	0.464	1.000	-0.805	1.000	-1.269	1.000
	g. *Christensenella*	0.03	0.02	0.00	0.02	0.02	0.01	-0.381	1.000	-2.276	0.14	-1.895	1.000	0.107	1.000	-1.280	0.857	-1.388	1.000	0.689	1.000	0.200	1.000	-0.307	1.000	0.581	1.000	1.588	1.000	1.007	1.000
	f. Christensenellaceae	0.01	0.01	0.01	0.00	0.00	0.02	-1.147	1.000	-1.765	1.00	-0.618	1.000	2.272	1.000	4.569	0.146	2.296	1.000	5.165	1.000	1.746	1.000	-1.168	1.000	2.893	1.000	-0.550	1.000	-3.444	1.000
	g. *Candidatus Arthromitus*	0.00	0.00	0.00	0.00	0.00	0.00	-0.009	1.000	1.882	1.00	1.890	1.000	-0.746	1.000	1.040	1.000	1.786	1.000	-0.239	1.000	0.498	1.000	0.603	1.000	0.507	1.000	-1.287	1.000	-1.794	1.000
	g. *Clostridium*	0.05	1.88	0.95	0.06	0.20	0.94	5.798	0.000	5.222	0.00	-0.575	1.000	1.584	1.000	4.981	0.000	3.397	0.002	0.151	1.000	4.365	0.000	0.392	1.000	-1.433	1.000	0.968	1.000	2.401	1.000
	f. Clostridiaceae	0.17	0.22	0.20	0.10	0.12	0.09	1.015	1.000	0.256	1.00	-0.758	1.000	0.036	1.000	0.736	1.000	0.700	1.000	1.890	1.000	2.869	0.021	1.411	1.000	1.854	1.000	2.169	1.000	0.315	1.000
	g. *SMB53*	0.00	0.00	0.00	0.00	0.00	0.00	-0.056	1.000	-0.050	1.00	0.006	1.000	-0.169	1.000	-0.102	1.000	0.067	1.000	-0.290	1.000	-0.177	1.000	-0.238	1.000	-0.121	1.000	-0.244	1.000	-0.123	1.000
	g. *Dehalobacterium*	0.00	0.00	0.00	0.00	0.00	0.01	1.033	1.000	1.869	1.00	0.836	1.000	-2.113	1.000	3.041	1.000	5.154	1.000	0.661	1.000	3.807	1.000	-0.511	1.000	2.774	1.000	-1.347	1.000	-4.121	1.000
	f. Dehalobacteriaceae	0.00	0.00	0.00	0.00	0.00	0.00	-0.056	1.000	-0.050	1.00	0.006	1.000	-0.851	1.000	0.366	1.000	1.217	1.000	-0.593	1.000	0.202	1.000	-1.009	1.000	0.258	1.000	-1.015	1.000	-1.273	1.000
	f. EtOH8	0.00	0.00	0.00	0.00	0.00	0.00	-3.938	1.000	-3.912	1.00	0.026	1.000	-0.681	1.000	-0.052	1.000	0.629	1.000	3.825	1.000	0.568	1.000	-0.036	1.000	4.506	1.000	-0.061	1.000	-4.567	1.000
	g. *Anaerofustis*	0.00	0.00	0.00	0.00	0.00	0.00	0.173	1.000	-0.434	1.00	-0.607	1.000	0.963	1.000	-0.442	1.000	-1.405	1.000	-0.241	1.000	-1.031	1.000	-0.233	1.000	-1.204	1.000	0.374	1.000	1.578	1.000
	g. *Pseudoramibacter_Eubacterium*	0.19	0.07	0.08	0.04	0.10	0.06	0.161	1.000	-0.247	1.00	-0.408	1.000	0.756	1.000	1.121	1.000	0.364	1.000	1.171	1.000	0.576	1.000	-0.197	1.000	0.415	1.000	0.212	1.000	-0.203	1.000
	f. Gracilibacteraceae	0.00	0.00	0.00	0.00	0.00	0.00	-0.335	1.000	0.079	1.00	0.413	1.000	-0.681	1.000	-0.052	1.000	0.629	1.000	2.077	1.000	2.423	1.000	2.207	1.000	2.758	1.000	1.794	1.000	-0.964	1.000
	g. *[Ruminococcus]*	2.74	0.17	0.03	1.35	0.31	0.03	-5.238	0.000	-6.988	0.00	-1.749	1.000	-2.428	0.588	-5.645	0.000	-3.217	0.029	1.326	1.000	-1.484	1.000	-0.016	1.000	3.754	0.014	1.733	1.000	-2.021	1.000
	g. *Anaerostipes*	0.00	0.01	0.01	0.00	0.00	0.01	3.576	1.000	3.801	1.00	0.225	1.000	-1.619	1.000	0.030	1.000	1.649	1.000	-2.302	1.000	2.893	1.000	1.469	1.000	-0.683	1.000	1.244	1.000	1.927	1.000
	g. *Blautia*	8.49	1.45	0.94	9.48	9.51	1.26	-2.400	0.001	-2.788	0.00	-0.388	1.000	0.107	1.000	-2.382	0.000	-2.489	0.000	0.192	1.000	-2.314	0.002	-0.214	1.000	0.085	1.000	0.174	1.000	0.089	1.000
	g. *Butyrivibrio*	0.01	0.00	0.02	0.01	0.05	0.02	-0.921	1.000	1.673	1.00	2.594	1.000	-0.016	1.000	-0.599	1.000	-0.584	1.000	-1.814	1.000	-2.719	1.000	0.458	1.000	-1.798	1.000	-2.136	1.000	-0.337	1.000
	g. *Coprococcus*	0.73	0.55	0.39	0.86	0.52	0.28	-0.838	1.000	-0.871	1.00	-0.033	1.000	-1.151	1.000	-1.315	0.390	-0.164	1.000	-0.090	1.000	0.224	1.000	0.355	1.000	1.062	1.000	0.388	1.000	-0.674	1.000
	g. *Dorea*	1.15	0.43	0.93	0.95	0.97	0.63	-1.387	0.020	-0.336	1.00	1.051	1.000	-0.210	1.000	-0.134	1.000	0.076	1.000	0.686	1.000	-0.491	1.000	0.484	1.000	0.896	1.000	-0.567	1.000	-1.463	1.000
	g. *Epulopiscium*	0.00	0.00	0.00	0.00	0.00	0.00	1.155	1.000	-0.075	1.00	-1.230	1.000	-0.681	1.000	-0.052	1.000	0.629	1.000	0.486	1.000	2.322	1.000	0.462	1.000	1.167	1.000	1.692	1.000	0.525	1.000
	g. *Lachnobacterium*	0.00	0.00	0.00	0.00	0.00	0.01	0.289	1.000	0.764	1.00	0.474	1.000	0.573	1.000	2.569	1.000	1.996	1.000	1.591	1.000	1.307	1.000	-0.215	1.000	1.018	1.000	-0.689	1.000	-1.707	1.000
	g. *Lachnospira*	0.00	0.00	0.00	0.00	0.00	0.00	0.723	1.000	1.293	1.00	0.570	1.000	-0.819	1.000	0.249	1.000	1.068	1.000	-0.313	1.000	1.230	1.000	0.731	1.000	0.506	1.000	0.161	1.000	-0.345	1.000
	g. *Moryella*	0.00	0.00	0.00	0.00	0.01	0.00	-0.772	1.000	-0.122	1.00	0.650	1.000	0.386	1.000	-0.341	1.000	-0.727	1.000	0.247	1.000	-0.911	1.000	0.466	1.000	-0.139	1.000	-0.184	1.000	-0.045	1.000
	f. Lachnospiraceae	5.62	4.71	5.75	4.35	5.65	6.34	0.019	1.000	0.283	1.00	0.264	1.000	0.032	1.000	1.075	0.084	1.043	0.277	0.635	1.000	0.622	1.000	-0.157	1.000	0.603	1.000	-0.421	1.000	-1.024	1.000
	g. *Roseburia*	0.01	0.24	0.22	0.00	0.01	0.14	2.138	0.905	4.620	0.01	2.482	1.000	1.440	1.000	6.099	0.002	4.660	0.028	2.351	1.000	3.049	0.506	0.872	1.000	0.912	1.000	-1.610	1.000	-2.522	1.000
	g. *Shuttleworthia*	0.00	0.03	0.01	0.00	0.01	0.01	2.258	1.000	3.143	1.00	0.886	1.000	0.833	1.000	2.419	1.000	1.586	1.000	-0.680	1.000	0.745	1.000	0.045	1.000	-1.513	1.000	-0.841	1.000	0.672	1.000
	o. Clostridiales	2.78	7.62	9.11	2.06	2.86	7.48	1.427	0.020	1.945	0.00	0.518	1.000	0.678	1.000	2.593	0.000	1.915	0.000	1.095	1.000	1.844	0.001	0.447	1.000	0.417	1.000	-0.071	1.000	-0.488	1.000

	f. Peptococcaceae	0.00	0.00	0.00	0.00	0.00	0.00	-0.056	1.000	-0.050	1.00	0.006	1.000	-0.296	1.000	-0.191	1.000	0.105	1.000	-0.557	1.000	-0.317	1.000	-0.416	1.000	-0.261	1.000	-0.422	1.000	-0.161	1.000
	g. *Peptococcus*	0.00	0.00	0.00	0.00	0.00	0.00	-0.056	1.000	-0.050	1.00	0.006	1.000	-0.455	1.000	0.187	1.000	0.642	1.000	-0.326	1.000	0.073	1.000	-0.563	1.000	0.129	1.000	-0.569	1.000	-0.698	1.000
	g. *rc4-4*	0.03	0.13	0.08	0.03	0.18	0.10	3.402	0.036	3.009	0.05	-0.393	1.000	3.716	0.264	3.608	0.011	-0.108	1.000	0.379	1.000	0.064	1.000	-0.220	1.000	-3.338	0.243	0.172	1.000	3.510	1.000
	f. Peptostreptococcaceae	0.06	0.05	0.01	0.03	0.02	0.02	-0.098	1.000	-0.851	1.00	-0.754	1.000	-0.440	1.000	-1.356	1.000	-0.916	1.000	0.136	1.000	0.479	1.000	0.641	1.000	0.577	1.000	1.395	1.000	0.818	1.000
	g. *Anaerotruncus*	0.01	0.00	0.00	0.00	0.00	0.00	-2.749	1.000	-1.438	1.00	1.311	1.000	-1.148	1.000	-0.734	1.000	0.414	1.000	1.614	1.000	0.013	1.000	0.909	1.000	2.762	1.000	-0.402	1.000	-3.164	1.000
	g. *Butyricicoccus*	0.00	0.00	0.00	0.00	0.00	0.00	-0.854	1.000	-1.496	1.00	-0.642	1.000	-2.755	1.000	-2.119	1.000	0.636	1.000	-0.411	1.000	1.490	1.000	0.212	1.000	2.344	1.000	0.853	1.000	-1.491	1.000
	g. *Faecalibacterium*	2.24	1.03	0.26	4.20	1.95	0.22	-1.832	0.216	-2.322	0.04	-0.489	1.000	-1.613	0.829	-4.160	0.000	-2.547	0.045	-1.060	1.000	-1.280	1.000	0.779	1.000	0.553	1.000	1.268	1.000	0.715	1.000
	f. Ruminococcaceae	5.02	10.65	17.26	7.75	6.05	13.33	1.349	0.036	2.080	0.00	0.731	1.000	-0.822	0.970	1.036	0.092	1.858	0.001	-0.659	1.000	1.512	0.016	0.385	1.000	0.163	1.000	-0.346	1.000	-0.509	1.000
	g. *Oscillospira*	2.61	1.83	1.74	3.74	2.20	1.42	-0.324	1.000	-0.330	1.00	-0.005	1.000	-1.183	0.309	-1.146	0.013	0.037	1.000	-0.398	1.000	0.460	1.000	0.418	1.000	0.785	1.000	0.423	1.000	-0.361	1.000
	g. *Ruminococcus*	1.10	3.41	3.91	1.32	1.30	3.15	2.516	0.009	2.653	0.00	0.137	1.000	0.405	1.000	2.448	0.005	2.044	0.077	0.243	1.000	2.354	0.021	0.448	1.000	-0.161	1.000	0.311	1.000	0.472	1.000
	g. *Acidaminococcus*	0.02	0.01	0.00	0.03	0.01	0.00	-1.949	0.890	-3.072	0.19	-1.123	1.000	0.447	1.000	-0.693	1.000	-1.140	1.000	0.792	1.000	-1.604	1.000	-1.587	1.000	0.345	1.000	-0.465	1.000	-0.809	1.000
	g. *Anaerovibrio*	0.01	0.04	0.02	0.00	0.01	0.03	0.969	1.000	0.860	1.00	-0.109	1.000	2.026	1.000	4.463	0.041	2.438	1.000	4.725	1.000	3.669	0.220	1.122	1.000	2.699	1.000	1.231	1.000	-1.468	1.000
	g. *Dialister*	0.00	0.02	0.04	0.01	0.02	0.04	3.150	0.085	4.427	0.00	1.277	1.000	0.954	1.000	3.584	0.011	2.630	0.134	-0.889	1.000	1.307	1.000	-0.047	1.000	-1.843	1.000	-1.323	1.000	0.520	1.000
	g. *Megasphaera*	0.19	0.05	0.03	0.04	0.15	0.04	1.014	1.000	-0.454	1.00	-1.469	1.000	2.624	0.821	1.968	0.511	-0.656	1.000	1.287	1.000	-0.323	1.000	-1.135	1.000	-1.337	1.000	0.333	1.000	1.671	1.000
	g. *Mitsuokella*	0.02	0.01	0.00	0.00	0.01	0.00	1.064	1.000	-1.767	1.00	-2.831	1.000	1.788	1.000	2.442	1.000	0.654	1.000	2.385	1.000	1.662	1.000	-1.824	1.000	0.597	1.000	1.007	1.000	0.410	1.000
	f. Veillonellaceae	0.01	0.06	0.10	0.02	0.01	0.07	1.927	0.426	2.390	0.13	0.463	1.000	-0.867	1.000	2.343	0.110	3.210	0.045	0.190	1.000	2.984	0.102	0.237	1.000	1.057	1.000	-0.226	1.000	-1.283	1.000
	g. *Phascolarctobacterium*	0.48	0.56	0.48	0.25	0.47	0.30	-0.208	1.000	-0.320	1.00	-0.112	1.000	0.621	1.000	0.681	1.000	0.061	1.000	1.239	1.000	0.410	1.000	0.237	1.000	0.618	1.000	0.349	1.000	-0.269	1.000
	g. *Succiniclasticum*	0.01	0.00	0.00	0.00	0.01	0.00	-0.149	1.000	-1.690	1.00	-1.541	1.000	1.280	1.000	-0.343	1.000	-1.623	1.000	1.629	1.000	0.199	1.000	0.281	1.000	0.348	1.000	1.823	1.000	1.474	1.000
	g. *Veillonella*	0.19	0.49	0.01	0.61	0.01	0.00	-2.017	1.000	-6.388	0.03	-4.372	1.000	-3.660	1.000	-4.308	0.547	-0.648	1.000	4.662	1.000	6.305	0.102	2.582	1.000	8.322	0.016	6.953	0.658	-1.369	1.000
	o. Natranaerobiales	0.00	0.00	0.00	0.00	0.00	0.00	0.232	1.000	0.153	1.00	-0.079	1.000	-0.681	1.000	-0.052	1.000	0.629	1.000	0.380	1.000	1.293	1.000	0.585	1.000	1.061	1.000	0.664	1.000	-0.397	1.000
	g. *[Eubacterium]*	0.97	0.25	0.05	0.36	0.10	0.06	-2.486	0.078	-3.991	0.00	-1.505	1.000	-1.851	0.822	-2.156	0.094	-0.304	1.000	1.459	1.000	0.825	1.000	-0.376	1.000	3.310	0.025	1.129	1.000	-2.182	1.000
	g. *Bulleidia*	0.02	0.03	0.01	0.01	0.02	0.04	1.209	1.000	0.137	1.00	-1.072	1.000	0.541	1.000	3.332	0.012	2.791	0.091	1.210	1.000	1.878	0.843	-1.985	1.000	0.669	1.000	-0.913	1.000	-1.582	1.000
	g. *Catenibacterium*	0.06	0.04	0.06	0.02	0.17	0.05	2.888	0.538	1.034	1.00	-1.854	1.000	5.126	0.309	5.095	0.013	-0.030	1.000	1.466	1.000	-0.772	1.000	-2.596	1.000	-3.660	1.000	-0.742	1.000	2.918	1.000
	g. *Clostridium*	0.00	0.00	0.00	0.00	0.00	0.00	-1.255	1.000	-1.289	1.00	-0.034	1.000	-0.384	1.000	-0.879	1.000	-0.495	1.000	0.130	1.000	-0.741	1.000	-0.280	1.000	0.514	1.000	-0.246	1.000	-0.760	1.000
	g. *Coprobacillus*	0.32	0.09	0.04	0.48	0.09	0.01	-0.626	1.000	-3.063	0.04	-2.437	1.000	-2.606	0.588	-6.058	0.000	-3.452	0.074	-1.164	1.000	0.816	1.000	1.831	1.000	1.442	1.000	4.268	0.108	2.826	1.000
	g. *Holdemania*	0.03	0.02	0.02	0.02	0.05	0.02	-0.457	1.000	-0.364	1.00	0.093	1.000	2.346	0.588	1.586	0.484	-0.760	1.000	1.655	1.000	-1.148	1.000	-0.295	1.000	-0.691	1.000	-0.388	1.000	0.302	1.000
	g. *L7A_E11*	0.00	0.00	0.00	0.00	0.00	0.00	-0.056	1.000	-0.050	1.00	0.006	1.000	0.380	1.000	-1.162	1.000	-1.543	1.000	-1.653	1.000	-2.089	1.000	-0.540	1.000	-2.033	1.000	-0.546	1.000	1.487	1.000
	f. Erysipelotrichaceae	3.05	0.75	0.34	2.76	3.29	0.66	-2.134	0.058	-2.900	0.00	-0.766	1.000	-0.322	1.000	-2.011	0.041	-1.689	0.333	0.023	1.000	-1.789	0.318	-0.866	1.000	0.345	1.000	-0.100	1.000	-0.445	1.000
	g. *p-75-a5*	0.01	0.03	0.02	0.01	0.01	0.02	1.436	1.000	1.257	1.00	-0.180	1.000	-0.139	1.000	1.457	0.782	1.596	1.000	-0.105	1.000	1.470	1.000	-0.306	1.000	0.033	1.000	-0.126	1.000	-0.159	1.000
	g. *RFN20*	0.10	0.24	0.05	0.09	0.20	0.07	2.934	0.216	0.662	1.00	-2.272	1.000	3.186	0.636	3.220	0.094	0.034	1.000	1.101	1.000	0.850	1.000	-1.456	1.000	-2.084	1.000	0.816	1.000	2.901	1.000
	g. *Sharpea*	0.07	0.25	0.62	0.07	0.38	0.32	3.336	0.057	4.525	0.00	1.189	1.000	2.586	0.822	5.699	0.000	3.113	0.091	2.018	1.000	2.767	0.318	0.844	1.000	-0.569	1.000	-0.345	1.000	0.223	1.000
Fusobacteria		1.87	0.28	0.14	1.98	0.82	3.03	-3.379	0.128	-4.256	0.05	-0.877	0.991	-2.418	0.689	2.810	0.237	5.228	0.001	3.218	0.160	2.257	0.496	-3.848	0.241	5.636	0.001	-2.971	1.000	-8.607	0.000
	g. *Cetobacterium*	0.00	0.00	0.00	0.00	0.00	0.00	-0.089	1.000	0.463	1.00	0.552	1.000	-0.808	1.000	0.889	1.000	1.697	1.000	-0.525	1.000	0.194	1.000	-0.951	1.000	0.283	1.000	-1.503	1.000	-1.786	1.000
	g. *Fusobacterium*	1.87	0.28	0.14	1.98	0.82	3.02	-3.138	0.148	-4.031	0.01	-0.893	1.000	-3.174	0.636	2.528	0.263	5.702	0.001	2.892	1.000	2.929	0.492	-3.666	1.000	6.067	0.006	-2.773	1.000	-8.840	0.000
	f. Fusobacteriaceae	0.00	0.00	0.00	0.00	0.00	0.00	-0.011	1.000	0.532	1.00	0.543	1.000	-0.140	1.000	-0.066	1.000	0.074	1.000	-0.101	1.000	0.028	1.000	0.497	1.000	0.039	1.000	-0.046	1.000	-0.085	1.000
Lentisphaerae	f. Victivallaceae	0.00	0.01	0.00	0.00	0.00	0.00	6.924	1.000	-2.559	1.00	-9.483	1.000	-3.679	1.000	5.292	1.000	8.971	1.000	-0.686	1.000	9.916	1.000	-8.537	1.000	2.992	1.000	0.946	1.000	-2.047	1.000
Proteobacteria		4.20	6.69	4.61	3.86	2.54	6.02	0.702	0.480	0.806	0.57	0.104	0.991	-0.514	0.964	0.933	0.756	1.448	0.167	0.038	0.991	1.254	0.496	-0.090	0.991	0.552	0.706	-0.194	1.000	-0.746	0.819
	g. *Mycoplana*	0.00	0.00	0.00	0.00	0.00	0.00	-0.056	1.000	-0.050	1.00	0.006	1.000	-0.630	1.000	0.565	1.000	1.195	1.000	-0.182	1.000	0.392	1.000	-0.796	1.000	0.448	1.000	-0.802	1.000	-1.250	1.000
	f. Caulobacteraceae	0.00	0.00	0.00	0.00	0.00	0.00	0.514	1.000	0.000	1.00	-0.514	1.000	0.843	1.000	2.370	1.000	1.527	1.000	-0.932	1.000	-1.261	1.000	-3.302	1.000	-1.775	1.000	-2.788	1.000	-1.013	1.000
	g. *Phenylobacterium*	0.00	0.00	0.00	0.00	0.00	0.00	-0.056	1.000	-0.050	1.00	0.006	1.000	-1.169	1.000	-0.510	1.000	0.659	1.000	-0.703	1.000	0.409	1.000	-0.243	1.000	0.465	1.000	-0.250	1.000	-0.715	1.000
	p. Alphaproteobacteria	0.01	0.00	0.00	0.00	0.00	0.00	-2.724	1.000	-2.615	1.00	0.110	1.000	-1.241	1.000	-0.631	1.000	0.609	1.000	2.589	1.000	1.105	1.000	0.606	1.000	3.830	1.000	0.496	1.000	-3.334	1.000
	o.RF32	1.76	0.47	0.56	0.55	1.05	0.58	0.621	1.000	0.843	1.00	0.221	1.000	0.891	1.000	1.051	1.000	0.160	1.000	1.138	1.000	0.869	1.000	0.930	1.000	0.248	1.000	0.709	1.000	0.461	1.000
	f. Beijerinckiaceae	0.00	0.00	0.00	0.00	0.00	0.00	-0.056	1.000	-0.050	1.00	0.006	1.000	-1.308	1.000	-0.095	1.000	1.214	1.000	-0.921	1.000	0.331	1.000	-0.877	1.000	0.387	1.000	-0.883	1.000	-1.270	1.000
	f. Brucellaceae	0.00	0.00	0.00	0.00	0.00	0.00	0.532	1.000	0.020	1.00	-0.512	1.000	-1.965	1.000	-3.157	1.000	-1.192	1.000	-3.303	1.000	-0.807	1.000	-0.127	1.000	-1.338	1.000	0.385	1.000	1.724	1.000
	g. *Ochrobactrum*	0.00	0.00	0.00	0.00	0.00	0.00	-0.056	1.000	-0.050	1.00	0.006	1.000	-0.294	1.000	-0.538	1.000	-0.244	1.000	-2.221	1.000	-1.983	1.000	-1.733	1.000	-1.927	1.000	-1.739	1.000	0.188	1.000
	g. *Devosia*	0.00	0.00	0.00	0.00	0.00	0.00	-0.437	1.000	0.171	1.00	0.608	1.000	-1.400	1.000	0.491	1.000	1.891	1.000	-0.410	1.000	0.552	1.000	-0.731	1.000	0.989	1.000	-1.339	1.000	-2.328	1.000
	c. Rhizobiales	0.00	0.00	0.00	0.00	0.00	0.00	-1.333	1.000	-1.308	1.00	0.025	1.000	0.510	1.000	-0.024	1.000	-0.534	1.000	1.115	1.000	-0.728	1.000	-0.169	1.000	0.605	1.000	-0.194	1.000	-0.799	1.000
	g. *Agrobacterium*	0.00	0.00	0.00	0.00	0.00	0.00	-0.138	1.000	-1.081	1.00	-0.944	1.000	-0.078	1.000	0.019	1.000	0.097	1.000	1.281	1.000	1.222	1.000	0.181	1.000	1.359	1.000	1.125	1.000	-0.234	1.000
	f. Rhodobacteraceae	0.00	0.00	0.00	0.00	0.00	0.00	-0.056	1.000	-0.050	1.00	0.006	1.000	-0.287	1.000	-0.134	1.000	0.153	1.000	-0.306	1.000	-0.075	1.000	-0.222	1.000	-0.019	1.000	-0.228	1.000	-0.209	1.000
	g. *Paracoccus*	0.00	0.00	0.00	0.00	0.00	0.00	-0.553	1.000	-0.525	1.00	0.028	1.000	-0.185	1.000	-0.100	1.000	0.085	1.000	0.550	1.000	0.182	1.000	0.125	1.000	0.735	1.000	0.097	1.000	-0.638	1.000
	g. *Novispirillum*	0.00	0.00	0.00	0.00	0.00	0.00	-0.553	1.000	-0.525	1.00	0.028	1.000	-0.681	1.000	-0.052	1.000	0.629	1.000	0.689	1.000	0.816	1.000	0.215	1.000	1.369	1.000	0.187	1.000	-1.183	1.000

	g. *Sphingomonas*	0.00	0.00	0.00	0.00	0.00	0.00	-0.056	1.000	-0.050	1.00	0.006	1.000	-0.110	1.000	0.027	1.000	0.137	1.000	-0.120	1.000	-0.066	1.000	-0.197	1.000	-0.010	1.000	-0.203	1.000	-0.193	1.000
	f. Alcaligenaceae	0.02	0.00	0.00	0.03	0.01	0.00	-3.342	0.204	-7.023	0.00	-3.681	1.000	-2.609	0.822	-4.868	0.011	-2.259	1.000	-0.772	1.000	-1.505	1.000	-2.927	1.000	1.837	1.000	0.754	1.000	-1.083	1.000
	g. *Sutterella*	0.78	0.76	1.08	0.61	0.59	0.89	0.772	1.000	1.615	0.11	0.843	1.000	-0.422	1.000	0.768	1.000	1.190	0.910	-0.255	1.000	0.939	1.000	0.592	1.000	0.167	1.000	-0.251	1.000	-0.418	1.000
	g. *Comamonas*	0.01	0.00	0.00	0.00	0.00	0.10	-27.416	1.000	-27.268	1.00	0.148	1.000	-1.402	1.000	12.920	1.000	14.322	0.226	12.468	1.000	-13.546	0.349	-27.719	0.002	13.870	1.000	-27.867	0.001	-41.738	0.000
	f. Comamonadaceae	0.00	0.00	0.00	0.00	0.00	0.00	-1.151	1.000	-1.146	1.00	0.005	1.000	-0.823	1.000	1.073	1.000	1.896	1.000	0.619	1.000	0.291	1.000	-1.600	1.000	1.442	1.000	-1.605	1.000	-3.047	1.000
	f. Oxalobacteraceae	0.00	0.00	0.00	0.00	0.00	0.00	0.125	1.000	0.301	1.00	0.175	1.000	-0.681	1.000	-0.052	1.000	0.629	1.000	0.236	1.000	1.043	1.000	0.589	1.000	0.917	1.000	0.413	1.000	-0.504	1.000
	g. *Oxalobacter*	0.00	0.00	0.00	0.00	0.00	0.00	-0.149	1.000	-0.066	1.00	0.083	1.000	-0.699	1.000	2.030	1.000	2.729	1.000	0.971	1.000	1.521	1.000	-1.125	1.000	1.670	1.000	-1.208	1.000	-2.878	1.000
	g. *Bilophila*	0.02	0.01	0.00	0.04	0.00	0.00	-2.323	1.000	-4.738	0.19	-2.415	1.000	-5.554	0.609	-1.954	1.000	3.601	1.000	1.081	1.000	4.313	0.843	-1.703	1.000	6.636	0.295	0.712	1.000	-5.924	1.000
	g. *Desulfovibrio*	0.32	0.23	0.22	0.34	0.17	0.15	-0.810	1.000	-0.128	1.00	0.682	1.000	-0.230	1.000	-0.918	1.000	-0.688	1.000	0.635	1.000	0.054	1.000	1.424	1.000	0.864	1.000	0.743	1.000	-0.122	1.000
	f. Desulfovibrionaceae	0.01	0.03	0.01	0.02	0.02	0.01	2.077	0.890	-0.365	1.00	-2.442	1.000	1.518	1.000	1.167	1.000	-0.351	1.000	0.065	1.000	0.624	1.000	-1.467	1.000	-1.453	1.000	0.975	1.000	2.428	1.000
	g. *Campylobacter*	0.00	0.00	0.00	0.00	0.00	0.00	-0.056	1.000	-0.050	1.00	0.006	1.000	0.651	1.000	0.051	1.000	-0.600	1.000	-0.094	1.000	-0.800	1.000	-0.194	1.000	-0.744	1.000	-0.200	1.000	0.544	1.000
	g. *Helicobacter*	0.00	0.00	0.00	0.00	0.00	0.00	-0.203	1.000	0.334	1.00	0.537	1.000	-0.681	1.000	-0.052	1.000	0.629	1.000	0.305	1.000	0.783	1.000	0.691	1.000	0.986	1.000	0.154	1.000	-0.832	1.000
	f. Helicobacteraceae	0.00	0.00	0.00	0.00	0.00	0.00	-0.056	1.000	-0.050	1.00	0.006	1.000	-0.296	1.000	-0.282	1.000	0.014	1.000	-0.305	1.000	-0.065	1.000	-0.073	1.000	-0.009	1.000	-0.079	1.000	-0.070	1.000
	f. Succinivibrionaceae	0.00	0.02	0.00	0.00	0.01	0.00	4.073	0.814	0.539	1.00	-3.534	1.000	2.935	1.000	1.396	1.000	-1.540	1.000	0.427	1.000	1.565	1.000	-0.429	1.000	-2.509	1.000	3.105	1.000	5.613	1.000
	g. *Ruminobacter*	0.00	0.01	0.01	0.00	0.00	0.00	1.523	1.000	4.322	1.00	2.799	1.000	-0.843	1.000	0.344	1.000	1.186	1.000	1.222	1.000	3.588	1.000	5.201	1.000	2.065	1.000	2.402	1.000	0.337	1.000
	g. *Succinivibrio*	0.70	4.92	2.64	0.40	0.17	4.10	3.250	0.001	3.265	0.00	0.015	1.000	0.064	1.000	5.112	0.000	5.048	0.000	1.684	1.000	4.870	0.000	-0.163	1.000	1.620	1.000	-0.178	1.000	-1.798	1.000
	g. *Rheinheimera*	0.00	0.00	0.00	0.00	0.00	0.00	-0.056	1.000	-0.050	1.00	0.006	1.000	-0.659	1.000	0.461	1.000	1.120	1.000	-0.182	1.000	0.421	1.000	-0.693	1.000	0.477	1.000	-0.699	1.000	-1.176	1.000
	g. *Citrobacter*	0.00	0.00	0.00	0.02	0.00	0.00	-17.002	1.000	0.264	1.00	17.266	1.000	0.134	1.000	-27.725	1.000	-27.859	0.000	-9.230	1.000	-26.366	0.000	18.760	1.000	-9.364	1.000	1.494	1.000	10.857	1.000
	g. *Escherichia*	0.00	0.00	0.00	0.00	0.00	0.00	0.481	1.000	-0.070	1.00	-0.551	1.000	-1.255	1.000	-1.031	1.000	0.224	1.000	-1.847	1.000	-0.110	1.000	-0.885	1.000	-0.591	1.000	-0.334	1.000	0.258	1.000
	f. Enterobacteriaceae	0.52	0.24	0.09	1.84	0.49	0.17	-3.598	0.020	-3.741	0.01	-0.143	1.000	-1.303	1.000	-3.515	0.011	-2.212	0.544	-0.393	1.000	-2.688	0.303	-0.619	1.000	0.910	1.000	-0.476	1.000	-1.386	1.000
	g. *Serratia*	0.00	0.00	0.00	0.00	0.00	0.00	-0.457	1.000	-0.472	1.00	-0.016	1.000	-0.638	1.000	-0.581	1.000	0.056	1.000	-0.052	1.000	0.129	1.000	0.057	1.000	0.585	1.000	0.072	1.000	-0.513	1.000
	g. *Tatlockia*	0.00	0.00	0.00	0.00	0.00	0.00	-0.056	1.000	-0.050	1.00	0.006	1.000	0.041	1.000	0.174	1.000	0.132	1.000	-0.058	1.000	-0.156	1.000	-0.282	1.000	-0.100	1.000	-0.288	1.000	-0.188	1.000
	g. *Aggregatibacter*	0.00	0.00	0.00	0.00	0.00	0.00	-0.056	1.000	-0.050	1.00	0.006	1.000	-0.669	1.000	-1.707	1.000	-1.038	1.000	-1.727	1.000	-1.113	1.000	-0.070	1.000	-1.057	1.000	-0.076	1.000	0.982	1.000
	g. *Gallibacterium*	0.03	0.01	0.00	0.00	0.00	0.00	-2.069	1.000	-3.008	1.00	-0.940	1.000	-2.995	1.000	-2.320	1.000	0.676	1.000	1.524	1.000	2.451	1.000	0.835	1.000	4.519	1.000	1.775	1.000	-2.744	1.000
	g. *Acinetobacter*	0.00	0.00	0.00	0.00	0.00	0.00	-1.338	1.000	-1.369	1.00	-0.031	1.000	0.597	1.000	0.201	1.000	-0.396	1.000	1.526	1.000	-0.409	1.000	-0.043	1.000	0.930	1.000	-0.012	1.000	-0.942	1.000
	g. *Enhydrobacter*	0.00	0.00	0.00	0.00	0.00	0.00	-0.056	1.000	-0.050	1.00	0.006	1.000	-0.169	1.000	-0.102	1.000	0.067	1.000	-0.290	1.000	-0.177	1.000	-0.238	1.000	-0.121	1.000	-0.244	1.000	-0.123	1.000
	f. Pseudomonadaceae	0.00	0.00	0.00	0.00	0.00	0.00	-0.567	1.000	-0.026	1.00	0.541	1.000	-0.465	1.000	0.799	1.000	1.264	1.000	0.230	1.000	0.128	1.000	-0.595	1.000	0.696	1.000	-1.136	1.000	-1.832	1.000
	g. *Pseudomonas*	0.00	0.00	0.00	0.00	0.00	0.00	-0.056	1.000	-0.050	1.00	0.006	1.000	-0.547	1.000	0.569	1.000	1.116	1.000	-0.066	1.000	0.425	1.000	-0.684	1.000	0.481	1.000	-0.690	1.000	-1.172	1.000
	g. *Steroidobacter*	0.00	0.00	0.00	0.00	0.00	0.00	-0.056	1.000	-0.050	1.00	0.006	1.000	-1.276	1.000	-0.602	1.000	0.673	1.000	-0.815	1.000	0.405	1.000	-0.262	1.000	0.461	1.000	-0.268	1.000	-0.729	1.000
	f. Xanthomonadaceae	0.00	0.00	0.00	0.00	0.00	0.00	-0.056	1.000	-0.050	1.00	0.006	1.000	-0.064	1.000	0.042	1.000	0.106	1.000	-0.058	1.000	-0.050	1.000	-0.150	1.000	0.006	1.000	-0.156	1.000	-0.162	1.000
Spirochaetes		0.33	1.76	1.58	0.02	0.02	0.44	2.733	0.128	3.772	0.08	1.039	0.991	1.204	0.964	6.677	0.000	5.472	0.001	4.073	0.160	5.602	0.002	1.168	0.991	2.869	0.243	0.129	1.000	-2.739	0.819
	g. *Sphaerochaeta*	0.00	0.00	0.00	0.00	0.00	0.00	0.504	1.000	-0.073	1.00	-0.577	1.000	-0.681	1.000	-0.052	1.000	0.629	1.000	0.350	1.000	1.536	1.000	0.329	1.000	1.031	1.000	0.907	1.000	-0.125	1.000
	g. *Treponema*	0.33	1.75	1.58	0.02	0.02	0.44	2.987	0.275	4.308	0.02	1.321	1.000	0.395	1.000	6.393	0.000	5.998	0.001	3.644	1.000	6.236	0.001	1.559	1.000	3.249	1.000	0.238	1.000	-3.011	1.000
Synergistetes		0.02	0.00	0.00	0.00	0.01	0.00	-5.187	0.128	-2.865	0.25	2.322	0.991	3.086	0.689	-1.081	0.983	-4.167	0.167	4.068	0.160	-4.204	0.496	2.284	0.991	0.983	0.768	-0.037	1.000	-1.020	0.982
	g. *Pyramidobacter*	0.00	0.00	0.00	0.00	0.00	0.00	-0.056	1.000	-0.050	1.00	0.006	1.000	1.355	1.000	0.412	1.000	-0.944	1.000	-0.666	1.000	-2.077	1.000	-1.127	1.000	-2.021	1.000	-1.134	1.000	0.888	1.000
	f. Synergistaceae	0.02	0.00	0.00	0.00	0.01	0.00	-8.216	0.538	-3.453	1.00	4.762	1.000	4.379	1.000	-25.999	1.000	-30.378	0.000	11.532	1.000	-1.062	1.000	34.078	0.000	7.153	1.000	29.316	0.000	22.162	1.000
	g. *Synergistes*	0.00	0.00	0.00	0.00	0.00	0.00	-0.618	1.000	-0.553	1.00	0.065	1.000	-0.681	1.000	-0.052	1.000	0.629	1.000	0.575	1.000	0.638	1.000	0.074	1.000	1.256	1.000	0.008	1.000	-1.247	1.000
Tenericutes		1.09	3.81	3.23	0.44	2.83	4.57	2.158	0.128	2.149	0.14	-0.009	0.991	3.391	0.015	4.176	0.000	0.786	0.994	1.244	0.991	0.012	0.996	-0.783	0.991	-2.147	0.243	-0.774	1.000	1.373	0.819
	o. Acholeplasmatales	0.00	0.00	0.00	0.00	0.00	0.00	0.488	1.000	-0.072	1.00	-0.560	1.000	-0.681	1.000	-0.052	1.000	0.629	1.000	0.141	1.000	1.310	1.000	0.121	1.000	0.822	1.000	0.681	1.000	-0.141	1.000
	g. *Anaeroplasma*	0.00	0.21	0.05	0.01	0.00	0.03	7.697	0.020	6.403	0.04	-1.295	1.000	-1.520	1.000	5.155	0.129	6.675	0.091	-0.145	1.000	9.073	0.005	1.103	1.000	1.375	1.000	2.397	1.000	1.022	1.000
	f. Anaeroplasmataceae	0.03	0.19	0.17	0.04	0.06	1.88	4.585	0.074	3.694	0.18	-0.890	1.000	3.481	0.970	7.737	0.000	4.256	0.150	2.205	1.000	3.309	0.591	-1.837	1.000	-1.276	1.000	-0.947	1.000	0.329	1.000
	o. RF39	1.05	3.32	2.94	0.40	2.77	2.64	2.133	0.204	2.286	0.09	0.153	1.000	3.112	0.264	4.373	0.000	1.261	1.000	1.915	1.000	0.936	1.000	-0.172	1.000	-1.197	1.000	-0.325	1.000	0.872	1.000
	o. ML615J-28	0.00	0.10	0.07	0.00	0.00	0.02	1.488	1.000	2.171	1.00	0.683	1.000	-0.483	1.000	2.396	1.000	2.880	1.000	1.462	1.000	3.433	1.000	1.236	1.000	1.945	1.000	0.553	1.000	-1.392	1.000
[Thermi]		0.00	0.00	0.00	0.00	0.00	0.00	-0.146	0.426	1.026	1.00	1.171	1.000	-0.035	1.000	0.007	1.000	0.042	1.000	0.299	1.000	0.189	1.000	1.318	1.000	0.334	1.000	0.147	1.000	-0.187	1.000
	g. *Deinococcus*	0.00	0.00	0.00	0.00	0.00	0.00	-0.066	1.000	0.479	1.000	0.545	1.000	-0.681	1.000	-0.052	1.000	0.629	1.000	0.220	0.996	0.834	1.000	0.750	0.999	0.900	1.000	0.205	1.000	-0.696	1.000
	g. *Thermus*	0.00	0.00	0.00	0.00	0.00	0.00	-0.102	1.000	0.474	1.000	0.576	1.000	-0.681	1.000	-0.052	1.000	0.629	1.000	0.162	0.996	0.740	1.000	0.687	0.999	0.842	1.000	0.111	1.000	-0.731	1.000
Verrucomicrobia	g. *Akkermansia*	0.11	0.01	0.01	0.01	0.01	0.00	-3.984	0.093	-2.190	0.473	1.793	1.000	-0.142	0.987	0.493	0.896	0.636	0.990	5.233	0.254	1.392	0.843	2.549	0.999	5.375	0.061	0.756	0.956	-4.619	0.473

Table S3. Ruminal KEGG L2

	Relative abundance						P-values									
	Early-weaned			Late-weaned			Early-weaned			Late-weaned						
**KEGG pathway**	**Wk5**	**Wk7**	**Wk9**	**Wk5**	**Wk7**	**Wk9**	**Wk 5 vs Wk 7**	**Wk 5 vs Wk 9**	**Wk 7 vs Wk 9**	**Wk 5vs Wk 7**	**Wk 5 vs Wk 9**	**Wk 7 vs Wk 9**	**Early vs Late Wk 5**	**Early vs Late Wk 7**	**Early vs Late Wk 9**	**(Early Wk7-Wk 5) vs (Late Wk9-Wk7)**
Cellular Processes																
Cell Communication	0.00	0.00	0.00	0.00	0.00	0.00	1.000	0.972	1.000	0.985	0.947	1.000	0.997	1.000	0.959	0.950
Cell Growth and Death	0.57	0.54	0.50	0.58	0.56	0.52	0.639	0.008	1.000	0.985	0.048	0.204	0.997	0.880	0.959	0.934
Cell Motility	1.64	1.57	1.37	1.44	1.45	1.43	0.924	0.440	1.000	0.985	0.835	0.806	0.943	0.880	0.959	0.934
Transport and Catabolism	0.32	0.24	0.20	0.32	0.25	0.22	0.000	0.000	1.000	0.066	0.000	0.204	0.943	0.880	0.959	0.886
Environmental Information Processing																
Membrane Transport	10.45	12.25	13.21	9.37	11.45	12.75	0.002	0.000	0.554	0.001	0.000	0.031	0.801	0.460	0.959	0.934
Signal Transduction	1.35	1.23	1.16	1.21	1.15	1.18	0.350	0.146	1.000	0.985	0.729	0.290	0.888	0.656	0.959	0.886
Signaling Molecules and Interaction	0.19	0.21	0.25	0.19	0.20	0.23	0.350	0.000	1.000	0.543	0.000	0.110	0.997	0.908	0.959	0.934
Genetic Information Processing																
Folding, Sorting and Degradation	2.58	2.48	2.36	2.65	2.61	2.43	0.987	0.146	0.554	0.985	0.415	0.204	0.943	0.707	0.959	0.934
Replication and Repair	9.57	9.41	9.27	9.87	9.49	9.40	0.407	0.324	1.000	0.985	0.460	0.227	0.943	0.880	0.959	0.950
Transcription	2.57	2.66	2.80	2.54	2.52	2.74	0.089	0.000	0.554	0.880	0.000	0.013	0.943	0.460	0.959	0.934
Translation	6.43	6.46	6.48	6.43	6.45	6.55	0.089	0.005	1.000	0.520	0.000	0.031	0.943	0.656	0.959	0.934
Human Diseases																
Cancers	0.12	0.12	0.11	0.12	0.14	0.12	0.697	0.636	1.000	0.300	0.835	0.204	0.997	0.656	0.959	0.886
Cardiovascular Diseases	0.00	0.00	0.00	0.00	0.00	0.00	0.000	0.000	1.000	0.018	0.000	0.483	0.943	1.000	0.959	0.111
Infectious Diseases	0.39	0.38	0.37	0.40	0.41	0.38	0.987	0.917	1.000	0.437	0.835	0.297	0.997	0.656	0.959	0.934
Metabolic Diseases	0.11	0.11	0.11	0.12	0.11	0.11	0.834	0.079	1.000	0.985	0.568	0.424	0.943	1.000	0.959	0.934
Neurodegenerative Diseases	0.15	0.10	0.12	0.12	0.09	0.11	0.034	0.283	1.000	0.543	0.947	0.228	0.943	0.880	0.959	0.125
Metabolism																
Amino Acid Metabolism	10.06	9.73	9.60	10.31	9.90	9.63	0.828	0.440	1.000	0.543	0.924	0.403	0.943	0.880	0.959	0.934
Biosynthesis of Other Secondary Metabolites	1.00	0.93	0.90	1.06	1.02	0.90	0.602	0.166	1.000	0.771	0.000	0.031	0.943	0.460	0.959	0.934
Carbohydrate Metabolism	10.71	10.54	11.02	10.72	10.73	10.82	0.613	0.002	0.554	0.697	0.009	0.204	0.943	0.990	0.959	0.934
Energy Metabolism	5.91	5.93	5.72	5.91	5.88	5.82	0.350	0.598	1.000	0.985	0.114	0.264	0.943	0.656	0.959	0.950
Enzyme Families	2.14	2.23	2.24	2.22	2.22	2.22	0.000	0.000	0.798	0.520	0.000	0.066	0.943	0.460	0.959	0.886
Glycan Biosynthesis and Metabolism	2.57	2.30	1.99	2.78	2.43	2.11	0.350	0.000	0.554	0.400	0.000	0.204	0.943	0.880	0.959	0.934
Lipid Metabolism	2.79	2.58	2.58	2.74	2.65	2.56	0.407	0.598	1.000	0.985	0.947	0.806	0.943	1.000	0.959	0.934
Metabolism of Cofactors and Vitamins	4.34	4.17	3.78	4.52	4.30	3.93	0.943	0.006	0.554	0.771	0.020	0.204	0.943	1.000	0.959	0.934
Metabolism of Other Amino Acids	1.54	1.51	1.52	1.55	1.58	1.51	0.943	0.470	1.000	0.691	0.636	0.806	0.997	0.880	0.959	0.934
Metabolism of Terpenoids and Polyketides	1.79	1.72	1.68	1.83	1.80	1.68	0.943	0.598	1.000	0.985	0.219	0.204	0.943	0.707	0.959	0.934
Nucleotide Metabolism	4.49	4.49	4.40	4.56	4.61	4.45	0.188	0.166	1.000	0.400	0.059	0.806	0.997	1.000	0.959	0.934
Xenobiotics Biodegradation and Metabolism	1.75	1.55	1.59	1.71	1.68	1.56	0.089	0.344	1.000	0.985	0.753	0.586	0.943	0.707	0.959	0.934
Organismal Systems							0.000	0.000	1.000	0.001	0.000	0.264	0.943	1.000	0.959	0.111
Circulatory System	0.01	0.00	0.00	0.00	0.00	0.00										
Digestive System	0.07	0.06	0.04	0.09	0.07	0.05	0.834	0.001	1.000	0.752	0.000	0.031	0.943	0.707	0.959	0.934
Endocrine System	0.34	0.25	0.21	0.38	0.31	0.22	0.003	0.000	1.000	0.279	0.000	0.001	0.943	0.460	0.959	0.934
Environmental Adaptation	0.15	0.15	0.14	0.15	0.15	0.15	0.269	0.470	1.000	0.465	0.316	0.841	0.997	1.000	0.959	0.934
Immune System	0.09	0.09	0.09	0.10	0.10	0.09	0.350	0.166	1.000	0.985	0.207	0.204	0.653	0.656	0.959	0.886
Nervous System	0.10	0.10	0.10	0.11	0.10	0.10	0.943	0.866	1.000	0.985	0.753	0.806	0.943	1.000	0.959	0.934
Unclassified																
Cellular Processes and Signaling	3.63	3.60	3.53	3.81	3.46	3.54	0.544	0.440	1.000	0.337	0.924	0.204	0.943	0.460	0.959	0.934
Genetic Information Processing	2.67	2.82	2.92	2.67	2.72	2.92	0.001	0.000	0.554	0.400	0.000	0.001	0.943	0.460	0.959	0.934
Metabolism	2.37	2.26	2.27	2.49	2.24	2.27	0.834	0.675	1.000	0.023	0.416	0.204	0.943	0.826	0.959	0.886
Poorly Characterized	4.94	5.19	5.32	4.88	5.10	5.28	0.000	0.000	0.554	0.023	0.000	0.008	0.943	0.460	0.959	0.934

Table 4. Ruminal KEGG L3

KEGG pathway	Early-wean			Late-wean (L)			P-values									
	Wk 5	Wk 7	Wk 9	Wk 5	Wk 7	Wk 9	E5 vs E7	E5 vs E9	E7 vs E9	L5vs L7	L5 vs L9	L7 vs L9	E5 vs L5	E7 vs L7	E9 vs L9	(E7-E5) vs (L9-L7)
Adherens junction	0.00	0.00	0.00	0.00	0.00	0.00	1.000	1.000	1.000	1.000	1.000	1.000	1.000	1.000	1.000	1.000
Focal adhesion	0.00	0.00	0.00	0.00	0.00	0.00	1.000	1.000	1.000	1.000	1.000	1.000	1.000	1.000	1.000	1.000
Tight junction	0.00	0.00	0.00	0.00	0.00	0.00	1.000	1.000	1.000	1.000	1.000	1.000	1.000	1.000	1.000	1.000
Apoptosis	0.01	0.00	0.00	0.00	0.00	0.00	0.000	0.000	1.000	0.003	0.000	0.798	0.564	1.000	1.000	0.006
Cell cycle	0.00	0.00	0.00	0.00	0.00	0.00	0.448	0.178	1.000	0.444	0.023	0.912	0.955	1.000	1.000	1.000
Cell cycle - Caulobacter	0.55	0.53	0.50	0.57	0.55	0.51	0.692	0.944	1.000	1.000	0.340	0.796	0.955	1.000	1.000	1.000
Cell cycle - yeast	0.00	0.00	0.00	0.00	0.00	0.00	1.000	1.000	1.000	1.000	1.000	1.000	1.000	1.000	1.000	1.000
Meiosis - yeast	0.01	0.01	0.00	0.01	0.01	0.00	0.830	0.132	1.000	1.000	0.627	0.796	1.000	1.000	1.000	1.000
Oocyte meiosis	0.00	0.00	0.00	0.00	0.00	0.00	1.000	1.000	1.000	1.000	1.000	1.000	1.000	1.000	1.000	1.000
p53 sig1ling pathway	0.00	0.00	0.00	0.00	0.00	0.00	0.000	0.000	1.000	0.015	0.000	0.819	0.714	1.000	1.000	0.145
Bacterial chemotaxis	0.33	0.34	0.28	0.27	0.28	0.30	1.000	0.761	1.000	1.000	0.423	0.961	0.895	1.000	1.000	1.000
Bacterial motility proteins	0.69	0.65	0.56	0.58	0.59	0.59	0.908	0.570	1.000	1.000	0.664	1.000	0.907	1.000	1.000	1.000
Cytoskeleton proteins	0.34	0.36	0.35	0.36	0.35	0.35	0.014	0.002	1.000	0.997	0.160	0.847	0.955	1.000	1.000	1.000
Flagellar assembly	0.28	0.23	0.17	0.24	0.22	0.20	0.497	0.080	1.000	1.000	0.755	1.000	1.000	1.000	1.000	1.000
Regulation of actin cytoskeleton	0.00	0.00	0.00	0.00	0.00	0.00	1.000	1.000	1.000	1.000	1.000	1.000	1.000	1.000	1.000	1.000
Endocytosis	0.00	0.00	0.00	0.00	0.00	0.00	1.000	1.000	1.000	1.000	1.000	0.005	1.000	0.000	1.000	1.000
Lysosome	0.12	0.09	0.09	0.11	0.08	0.09	0.184	0.248	1.000	0.494	0.619	0.912	1.000	1.000	1.000	1.000
Peroxisome	0.21	0.15	0.11	0.21	0.17	0.13	0.000	0.000	0.258	0.257	0.000	0.014	1.000	1.000	1.000	1.000
Phagosome	0.00	0.00	0.00	0.00	0.00	0.00	0.448	0.178	1.000	0.444	0.023	0.912	0.955	1.000	1.000	1.000
ABC transporters	2.66	3.20	3.31	2.33	3.01	3.24	0.000	0.000	1.000	0.003	0.000	0.107	0.564	1.000	1.000	1.000
Bacterial secretion system	0.66	0.58	0.50	0.68	0.65	0.53	0.409	0.000	0.258	1.000	0.012	0.054	1.000	1.000	1.000	1.000
Phosphotransferase system (PTS)	0.41	0.75	1.14	0.30	0.58	0.99	0.073	0.000	0.675	0.257	0.000	0.342	0.955	1.000	1.000	1.000
Secretion system	1.18	1.07	1.00	1.11	1.13	1.04	0.482	0.049	1.000	0.560	0.619	0.995	0.564	1.000	1.000	1.000
Transporters	5.55	6.63	7.27	4.95	6.08	6.95	0.001	0.000	0.548	0.015	0.000	0.040	0.564	0.886	1.000	1.000
Calcium sig1ling pathway	0.00	0.00	0.00	0.00	0.00	0.00	0.033	0.002	1.000	0.444	0.001	0.386	1.000	1.000	1.000	1.000
ErbB sig1ling pathway	0.00	0.00	0.00	0.00	0.00	0.00	1.000	1.000	1.000	1.000	1.000	1.000	1.000	1.000	1.000	1.000
Hedgehog sig1ling pathway	0.00	0.00	0.00	0.00	0.00	0.00	1.000	1.000	1.000	1.000	1.000	1.000	1.000	1.000	1.000	1.000
MAPK sig1ling pathway	0.00	0.00	0.00	0.00	0.00	0.00	1.000	1.000	1.000	1.000	1.000	1.000	1.000	1.000	1.000	1.000
MAPK sig1ling pathway - yeast	0.05	0.05	0.06	0.05	0.05	0.05	0.828	0.006	0.529	0.967	0.148	0.856	1.000	1.000	1.000	1.000
mTOR sig1ling pathway	0.00	0.00	0.00	0.00	0.00	0.00	0.448	0.178	1.000	0.444	0.023	0.912	0.955	1.000	1.000	1.000
Notch sig1ling pathway	0.00	0.00	0.00	0.00	0.00	0.00	1.000	1.000	1.000	1.000	1.000	1.000	1.000	1.000	1.000	1.000
Phosphatidylinositol sig1ling system	0.10	0.08	0.08	0.09	0.08	0.08	0.001	0.065	0.675	1.000	0.664	1.000	0.479	1.000	1.000	0.374
TGF-beta sig1ling pathway	0.00	0.00	0.00	0.00	0.00	0.00	1.000	1.000	1.000	1.000	1.000	1.000	1.000	1.000	1.000	1.000
Two-component system	1.21	1.10	1.02	1.07	1.01	1.04	0.635	0.283	1.000	1.000	0.445	0.507	0.564	1.000	1.000	1.000
VEGF sig1ling pathway	0.00	0.00	0.00	0.00	0.00	0.00	1.000	1.000	1.000	1.000	1.000	1.000	1.000	1.000	1.000	1.000
Wnt sig1ling pathway	0.00	0.00	0.00	0.00	0.00	0.00	1.000	1.000	1.000	1.000	1.000	1.000	1.000	1.000	1.000	1.000
Bacterial toxins	0.12	0.15	0.21	0.11	0.14	0.19	0.019	0.000	0.258	0.257	0.000	0.019	1.000	1.000	1.000	1.000
CAM ligands	0.00	0.00	0.00	0.00	0.00	0.00	1.000	1.000	1.000	1.000	1.000	1.000	1.000	1.000	1.000	1.000
Cellular antigens	0.06	0.04	0.02	0.07	0.05	0.03	0.209	0.000	0.258	0.503	0.000	0.054	0.965	1.000	1.000	1.000
Cytokine receptors	0.00	0.00	0.00	0.00	0.00	0.00	1.000	1.000	1.000	1.000	1.000	1.000	1.000	1.000	1.000	1.000
Cytokine-cytokine receptor interaction	0.00	0.00	0.00	0.00	0.00	0.00	1.000	1.000	1.000	1.000	1.000	1.000	1.000	1.000	1.000	1.000
ECM-receptor interaction	0.00	0.00	0.00	0.00	0.00	0.00	1.000	1.000	1.000	1.000	1.000	1.000	1.000	1.000	1.000	1.000
G protein-coupled receptors	0.00	0.00	0.00	0.00	0.00	0.00	1.000	1.000	1.000	1.000	1.000	1.000	1.000	1.000	1.000	1.000
Glycan bindng proteins	0.00	0.00	0.00	0.00	0.00	0.00	1.000	1.000	1.000	1.000	1.000	1.000	1.000	1.000	1.000	1.000
GTP-binding proteins	0.00	0.00	0.00	0.00	0.00	0.00	1.000	1.000	1.000	1.000	1.000	1.000	1.000	1.000	1.000	1.000
Ion channels	0.02	0.01	0.01	0.01	0.02	0.01	0.403	0.111	1.000	0.054	0.035	0.829	0.439	1.000	1.000	1.000
Neuroactive ligand-receptor interaction	0.00	0.00	0.00	0.00	0.00	0.00	1.000	1.000	1.000	1.000	1.000	1.000	1.000	1.000	1.000	1.000
Chaperones and folding catalysts	1.06	1.00	0.92	1.12	1.05	0.96	1.000	0.205	0.675	0.819	0.028	0.602	0.714	1.000	1.000	1.000
Proteasome	0.05	0.05	0.05	0.05	0.05	0.05	0.012	0.002	1.000	0.666	0.183	1.000	0.714	1.000	1.000	1.000
Protein export	0.68	0.66	0.63	0.69	0.69	0.64	0.372	0.531	1.000	0.444	0.369	0.912	1.000	1.000	1.000	1.000
Protein processing in endoplasmic reticulum	0.09	0.08	0.07	0.10	0.09	0.07	1.000	0.065	0.574	0.772	0.002	0.154	0.587	1.000	1.000	1.000
R1 degradation	0.50	0.50	0.50	0.51	0.51	0.51	0.008	0.000	0.638	0.666	0.003	0.262	0.955	1.000	1.000	1.000
Sulfur relay system	0.20	0.20	0.19	0.18	0.21	0.20	0.898	0.837	1.000	0.163	0.004	1.000	0.657	1.000	1.000	1.000
Ubiquitin system	0.00	0.00	0.00	0.00	0.00	0.00	0.000	0.000	1.000	0.444	0.000	0.044	1.000	0.020	1.000	0.617
Base excision repair	0.46	0.45	0.46	0.46	0.44	0.46	0.325	0.001	0.548	1.000	0.002	0.014	1.000	1.000	1.000	1.000
Chromosome	1.59	1.59	1.53	1.67	1.61	1.56	0.014	0.010	1.000	1.000	0.453	0.856	0.714	1.000	1.000	1.000
D1 repair and recombi1tion proteins	3.06	2.99	2.95	3.17	3.04	2.99	0.059	0.001	1.000	1.000	0.089	0.275	0.898	1.000	1.000	1.000
D1 replication	0.74	0.74	0.74	0.75	0.73	0.75	0.036	0.000	0.574	1.000	0.000	0.010	1.000	1.000	1.000	1.000
D1 replication proteins	1.35	1.32	1.30	1.38	1.31	1.33	0.206	0.040	1.000	1.000	0.035	0.071	1.000	1.000	1.000	1.000
Homologous recombi1tion	1.04	1.02	1.01	1.07	1.05	1.02	0.073	0.001	1.000	1.000	0.097	0.663	0.955	1.000	1.000	1.000
Mismatch repair	0.88	0.87	0.84	0.90	0.85	0.86	0.063	0.010	1.000	1.000	0.078	0.090	1.000	1.000	1.000	1.000
Non-homologous end-joining	0.02	0.00	0.00	0.02	0.01	0.00	0.000	0.000	1.000	1.000	0.002	0.054	1.000	0.044	1.000	1.000
Nucleotide excision repair	0.43	0.43	0.42	0.44	0.44	0.43	0.007	0.001	1.000	0.466	0.007	0.628	1.000	1.000	1.000	1.000
Basal transcription factors	0.01	0.01	0.01	0.00	0.01	0.01	0.375	0.248	1.000	0.523	0.110	1.000	1.000	1.000	1.000	1.000
R1 polymerase	0.21	0.21	0.22	0.19	0.21	0.22	0.089	0.006	1.000	0.158	0.000	0.147	0.714	1.000	1.000	1.000
Spliceosome	0.00	0.00	0.00	0.00	0.00	0.00	1.000	1.000	1.000	1.000	1.000	1.000	1.000	1.000	1.000	1.000
Transcription factors	1.39	1.50	1.68	1.31	1.37	1.60	0.066	0.000	0.529	0.553	0.000	0.050	0.965	1.000	1.000	1.000
Transcription machinery	0.97	0.94	0.90	1.03	0.94	0.91	0.372	0.428	1.000	0.424	0.377	0.819	0.564	1.000	1.000	1.000
Aminoacyl-tR1 biosynthesis	1.33	1.33	1.34	1.31	1.32	1.35	0.025	0.000	0.695	0.444	0.000	0.034	0.968	1.000	1.000	1.000
mR1 surveillance pathway	0.00	0.00	0.00	0.00	0.00	0.00	0.449	0.179	1.000	0.444	0.023	0.912	0.955	1.000	1.000	1.000
Ribosome	2.79	2.74	2.69	2.81	2.80	2.74	0.072	0.009	1.000	0.602	0.005	0.360	1.000	1.000	1.000	1.000

Ribosome Biogenesis	1.48	1.53	1.58	1.49	1.49	1.58	0.001	0.000	0.529	0.602	0.000	0.005	1.000	0.686	1.000	1.000
Ribosome biogenesis in eukaryotes	0.07	0.07	0.07	0.06	0.07	0.07	0.585	0.644	1.000	0.586	0.026	0.796	0.968	1.000	1.000	1.000
R1 transport	0.14	0.16	0.17	0.12	0.13	0.17	0.070	0.001	1.000	0.509	0.000	0.014	0.854	0.686	1.000	1.000
Translation factors	0.62	0.63	0.63	0.63	0.64	0.63	0.000	0.000	0.675	0.257	0.000	0.052	1.000	1.000	1.000	1.000
Bladder cancer	0.00	0.00	0.00	0.00	0.00	0.00	0.828	0.047	1.000	1.000	0.704	1.000	0.714	1.000	1.000	1.000
Chronic myeloid leukemia	0.00	0.00	0.00	0.00	0.00	0.00	1.000	1.000	1.000	1.000	1.000	1.000	1.000	1.000	1.000	1.000
Colorectal cancer	0.00	0.00	0.00	0.00	0.00	0.00	0.000	0.000	1.000	0.015	0.000	0.805	0.714	1.000	1.000	0.145
Glioma	0.00	0.00	0.00	0.00	0.00	0.00	1.000	1.000	1.000	1.000	1.000	1.000	1.000	1.000	1.000	1.000
Pancreatic cancer	0.00	0.00	0.00	0.00	0.00	0.00	1.000	1.000	1.000	1.000	1.000	1.000	1.000	1.000	1.000	1.000
Pathways in cancer	0.06	0.06	0.06	0.06	0.07	0.06	0.139	0.067	1.000	0.203	0.427	0.507	1.000	1.000	1.000	0.519
Prostate cancer	0.04	0.05	0.05	0.05	0.05	0.05	0.066	0.007	1.000	1.000	0.891	1.000	0.564	1.000	1.000	1.000
Re1l cell carcinoma	0.01	0.01	0.01	0.01	0.02	0.01	0.394	0.572	1.000	0.003	0.009	0.433	0.854	1.000	1.000	0.941
Small cell lung cancer	0.00	0.00	0.00	0.00	0.00	0.00	0.000	0.000	1.000	0.015	0.000	0.805	0.714	1.000	1.000	0.145
Arrhythmogenic right ventricular cardiomyopathy (ARVC)	0.00	0.00	0.00	0.00	0.00	0.00	1.000	1.000	1.000	1.000	1.000	1.000	1.000	1.000	1.000	1.000
Dilated cardiomyopathy (DCM)	0.00	0.00	0.00	0.00	0.00	0.00	1.000	1.000	1.000	1.000	1.000	1.000	1.000	1.000	1.000	1.000
Hypertrophic cardiomyopathy (HCM)	0.00	0.00	0.00	0.00	0.00	0.00	0.051	0.002	1.000	0.848	0.132	0.911	1.000	1.000	1.000	1.000
Viral myocarditis	0.00	0.00	0.00	0.00	0.00	0.00	0.000	0.000	1.000	0.015	0.000	0.829	0.714	1.000	1.000	0.145
Primary immunodeficiency	0.04	0.04	0.04	0.04	0.04	0.04	1.000	0.644	1.000	0.997	0.174	0.153	1.000	1.000	1.000	1.000
Rheumatoid arthritis	0.00	0.00	0.00	0.00	0.00	0.00	1.000	1.000	1.000	1.000	1.000	1.000	1.000	1.000	1.000	1.000
Systemic lupus erythematosus	0.00	0.00	0.00	0.00	0.00	0.00	0.001	0.000	1.000	0.772	0.007	0.216	1.000	1.000	1.000	1.000
African trypanosomiasis	0.00	0.00	0.00	0.00	0.00	0.00	0.000	0.000	1.000	0.015	0.000	0.912	0.714	1.000	1.000	0.006
Amoebiasis	0.01	0.00	0.00	0.00	0.00	0.00	0.000	0.000	1.000	0.444	0.000	0.309	0.854	1.000	1.000	1.000
Bacterial invasion of epithelial cells	0.00	0.00	0.00	0.00	0.00	0.00	0.002	0.242	1.000	0.028	0.006	1.000	0.787	1.000	1.000	0.374
Chagas disease (American trypanosomiasis)	0.00	0.00	0.00	0.00	0.00	0.00	0.000	0.000	1.000	0.178	0.016	1.000	0.895	1.000	1.000	0.145
Epithelial cell sig1ling in Helicobacter pylori infection	0.09	0.09	0.09	0.10	0.09	0.09	0.005	0.001	1.000	0.997	0.866	0.693	0.564	1.000	1.000	1.000
Hepatitis C	0.00	0.00	0.00	0.00	0.00	0.00	0.448	0.178	1.000	0.444	0.023	0.912	0.955	1.000	1.000	1.000
Influenza A	0.00	0.00	0.00	0.00	0.00	0.00	0.150	0.117	1.000	1.000	0.499	0.869	0.564	1.000	1.000	1.000
Leishmaniasis	0.00	0.00	0.00	0.00	0.00	0.00	1.000	1.000	1.000	1.000	1.000	1.000	1.000	1.000	1.000	1.000
Measles	0.00	0.00	0.00	0.00	0.00	0.00	0.448	0.178	1.000	0.444	0.023	0.912	0.955	1.000	1.000	1.000
Pathogenic Escherichia coli infection	0.00	0.00	0.00	0.00	0.00	0.00	1.000	1.000	1.000	1.000	1.000	1.000	1.000	1.000	1.000	1.000
Pertussis	0.03	0.01	0.00	0.03	0.02	0.01	0.002	0.000	0.258	0.853	0.000	0.014	1.000	0.443	1.000	1.000
Shigellosis	0.00	0.00	0.00	0.00	0.00	0.00	1.000	1.000	1.000	1.000	1.000	1.000	1.000	1.000	1.000	1.000
Staphylococcus aureus infection	0.03	0.04	0.04	0.02	0.06	0.04	0.828	0.670	1.000	0.007	0.007	0.693	0.564	1.000	1.000	1.000
Toxoplasmosis	0.00	0.00	0.00	0.00	0.00	0.00	0.000	0.000	1.000	0.015	0.000	0.805	0.714	1.000	1.000	0.145
Tuberculosis	0.16	0.15	0.16	0.16	0.15	0.16	0.828	0.027	0.574	0.870	0.162	0.089	1.000	1.000	1.000	1.000
Vibrio cholerae infection	0.00	0.00	0.00	0.00	0.00	0.00	0.485	0.200	1.000	0.444	0.021	0.912	0.955	1.000	1.000	1.000
Vibrio cholerae pathogenic cycle	0.07	0.07	0.07	0.08	0.08	0.08	0.019	0.003	1.000	1.000	1.000	1.000	0.439	1.000	1.000	1.000
Type I diabetes mellitus	0.06	0.05	0.05	0.06	0.05	0.05	0.122	0.074	1.000	0.997	0.016	0.275	1.000	1.000	1.000	1.000
Type II diabetes mellitus	0.06	0.06	0.07	0.06	0.06	0.07	0.089	0.000	0.529	1.000	0.015	0.216	0.968	1.000	1.000	1.000
Alzheimer's disease	0.07	0.05	0.05	0.06	0.05	0.05	0.058	0.034	1.000	0.966	0.888	1.000	0.714	1.000	1.000	1.000
Amyotrophic lateral sclerosis (ALS)	0.02	0.01	0.02	0.01	0.01	0.02	0.830	0.424	0.675	1.000	0.023	0.178	0.568	1.000	1.000	1.000
Huntington's disease	0.05	0.02	0.03	0.04	0.03	0.03	0.000	0.011	1.000	0.424	0.183	1.000	0.955	1.000	1.000	0.164
Parkinson's disease	0.01	0.00	0.00	0.01	0.00	0.00	0.000	0.000	1.000	0.003	0.000	0.626	0.965	1.000	1.000	0.145
Prion diseases	0.00	0.01	0.02	0.00	0.00	0.02	0.005	0.000	1.000	0.203	0.000	0.014	0.714	0.686	1.000	1.000
Alanine, aspartate and glutamate metabolism	1.14	1.12	1.10	1.20	1.19	1.10	0.072	0.003	1.000	0.963	1.000	0.912	0.564	1.000	1.000	0.941
Amino acid related enzymes	1.61	1.62	1.58	1.62	1.65	1.60	0.000	0.000	1.000	0.097	0.000	0.263	1.000	1.000	1.000	1.000
Arginine and proline metabolism	1.19	1.15	1.12	1.26	1.16	1.12	0.150	0.023	1.000	0.444	0.627	0.829	0.564	1.000	1.000	1.000
Cysteine and methionine metabolism	0.95	0.99	0.99	0.99	0.98	0.98	0.000	0.000	0.675	0.645	0.000	0.089	0.854	1.000	1.000	1.000
Glycine, serine and threonine metabolism	0.87	0.83	0.78	0.93	0.85	0.80	0.828	0.853	1.000	0.260	0.014	1.000	0.564	1.000	1.000	1.000
Histidine metabolism	0.67	0.65	0.69	0.68	0.65	0.67	0.692	0.001	0.398	1.000	0.070	0.119	1.000	1.000	1.000	1.000
Lysine biosynthesis	0.81	0.83	0.86	0.84	0.79	0.84	0.057	0.000	0.550	1.000	0.031	0.071	1.000	1.000	1.000	1.000
Lysine degradation	0.14	0.11	0.12	0.12	0.11	0.12	0.214	0.420	1.000	0.967	0.870	0.663	0.714	1.000	1.000	0.953
Phenylalanine metabolism	0.20	0.18	0.16	0.22	0.19	0.16	0.090	0.001	0.795	0.203	0.000	0.040	0.564	1.000	1.000	1.000
Phenylalanine, tyrosine and tryptophan biosynthesis	0.88	0.88	0.86	0.95	0.91	0.86	0.032	0.005	1.000	1.000	1.000	1.000	0.564	1.000	1.000	1.000
Tryptophan metabolism	0.17	0.13	0.12	0.13	0.14	0.12	0.061	0.012	1.000	1.000	1.000	1.000	0.564	1.000	1.000	1.000
Tyrosine metabolism	0.36	0.35	0.36	0.38	0.37	0.35	0.825	0.029	0.583	1.000	0.730	1.000	1.000	1.000	1.000	1.000
Valine, leucine and isoleucine biosynthesis	0.77	0.70	0.69	0.75	0.69	0.70	0.613	0.906	1.000	0.772	0.310	0.131	0.955	1.000	1.000	0.617
Valine, leucine and isoleucine degradation	0.29	0.21	0.17	0.25	0.22	0.18	0.004	0.000	0.574	0.827	0.023	0.508	0.714	1.000	1.000	1.000
beta-Lactam resistance	0.02	0.01	0.01	0.01	0.02	0.01	0.030	0.000	1.000	0.444	0.521	0.076	0.714	0.686	1.000	1.000
Betalain biosynthesis	0.00	0.00	0.00	0.00	0.00	0.00	0.423	0.086	1.000	0.477	0.202	1.000	1.000	1.000	1.000	1.000
Butirosin and neomycin biosynthesis	0.08	0.08	0.09	0.10	0.09	0.08	0.184	0.009	1.000	1.000	0.774	0.965	0.564	1.000	1.000	1.000
Caffeine metabolism	0.00	0.00	0.00	0.00	0.00	0.00	0.014	0.025	1.000	0.681	0.183	1.000	1.000	1.000	1.000	1.000
Clavulanic acid biosynthesis	0.00	0.00	0.00	0.00	0.00	0.00	1.000	1.000	1.000	1.000	1.000	1.000	1.000	1.000	1.000	1.000
Flavone and flavonol biosynthesis	0.01	0.00	0.00	0.00	0.00	0.00	0.003	0.000	1.000	0.444	0.139	1.000	0.564	1.000	1.000	0.478
Flavonoid biosynthesis	0.01	0.00	0.00	0.01	0.01	0.00	0.064	0.048	1.000	1.000	0.023	0.302	1.000	0.886	1.000	1.000
Indole alkaloid biosynthesis	0.00	0.00	0.00	0.00	0.00	0.00	0.502	0.117	1.000	0.482	0.142	1.000	1.000	1.000	1.000	1.000
Isoflavonoid biosynthesis	0.00	0.00	0.00	0.00	0.00	0.00	0.005	0.000	1.000	1.000	0.000	0.005	1.000	0.045	1.000	1.000
Isoquinoline alkaloid biosynthesis	0.06	0.06	0.04	0.07	0.06	0.05	0.828	0.070	0.356	1.000	0.070	0.305	0.968	1.000	1.000	1.000
Novobiocin biosynthesis	0.15	0.15	0.14	0.15	0.16	0.14	0.022	0.728	0.529	0.645	1.000	0.607	0.975	1.000	1.000	0.374
Penicillin and cephalosporin biosynthesis	0.02	0.01	0.01	0.01	0.02	0.01	0.001	0.000	0.791	0.772	0.118	0.054	0.564	0.855	1.000	1.000
Phenylpropanoid biosynthesis	0.17	0.16	0.18	0.20	0.17	0.18	0.945	0.157	0.548	0.602	0.678	0.995	0.854	1.000	1.000	1.000
Stilbenoid, diarylheptanoid and gingerol biosynthesis	0.00	0.00	0.00	0.00	0.00	0.00	0.000	0.000	1.000	0.257	0.014	1.000	1.000	1.000	1.000	0.191
Streptomycin biosynthesis	0.36	0.32	0.31	0.37	0.36	0.31	0.692	0.622	1.000	1.000	0.058	0.216	0.955	1.000	1.000	1.000
Tropane, piperidine and pyridine alkaloid biosynthesis	0.12	0.13	0.12	0.13	0.13	0.12	0.000	0.095	0.529	1.000	0.293	0.829	0.498	1.000	1.000	0.145

Amino sugar and nucleotide sugar metabolism	1.45	1.67	1.83	1.46	1.58	1.76	0.000	0.000	0.258	0.252	0.000	0.014	1.000	1.000	1.000	1.000
Ascorbate and aldarate metabolism	0.14	0.12	0.12	0.11	0.14	0.12	0.679	0.531	1.000	0.281	0.309	0.796	0.568	1.000	1.000	1.000
Butanoate metabolism	0.66	0.57	0.53	0.62	0.61	0.54	0.014	0.000	1.000	1.000	0.369	0.547	0.564	1.000	1.000	1.000
C5-Branched dibasic acid metabolism	0.31	0.30	0.29	0.29	0.26	0.29	0.813	0.821	1.000	0.997	0.033	0.052	0.854	0.443	1.000	1.000
Citrate cycle (TCA cycle)	0.73	0.55	0.43	0.72	0.61	0.49	0.002	0.000	0.258	0.444	0.000	0.068	1.000	1.000	1.000	1.000
Fructose and mannose metabolism	0.93	1.04	1.16	0.99	1.04	1.09	0.002	0.000	0.529	0.505	0.002	0.342	0.968	1.000	1.000	1.000
Galactose metabolism	0.73	0.71	0.78	0.72	0.71	0.74	0.801	0.003	0.452	1.000	0.057	0.430	1.000	1.000	1.000	1.000
Glycolysis / Gluconeogenesis	1.15	1.19	1.37	1.14	1.13	1.32	0.150	0.000	0.258	0.997	0.000	0.014	1.000	1.000	1.000	1.000
Glyoxylate and dicarboxylate metabolism	0.53	0.51	0.47	0.54	0.53	0.47	0.990	0.149	0.548	0.997	0.252	0.216	1.000	1.000	1.000	1.000
Inositol phosphate metabolism	0.10	0.07	0.06	0.09	0.08	0.07	0.000	0.000	0.574	0.778	0.061	0.796	0.439	1.000	1.000	0.419
Pentose and glucuro1te interconversions	0.56	0.46	0.43	0.61	0.55	0.44	0.154	0.034	1.000	0.667	0.000	0.092	0.854	0.886	1.000	1.000
Pentose phosphate pathway	0.84	0.91	0.97	0.84	0.91	0.94	0.005	0.000	0.574	0.257	0.000	0.342	1.000	1.000	1.000	1.000
Propanoate metabolism	0.53	0.50	0.53	0.49	0.50	0.51	1.000	0.078	0.548	0.653	0.001	0.178	0.564	1.000	1.000	1.000
Pyruvate metabolism	1.06	0.97	1.04	1.03	1.02	1.01	0.830	0.086	0.452	0.997	0.065	0.626	0.968	1.000	1.000	1.000
Starch and sucrose metabolism	1.00	0.97	1.03	1.08	1.06	1.00	0.411	0.002	0.563	1.000	0.976	1.000	0.714	1.000	1.000	1.000
Carbon fixation in photosynthetic organisms	0.69	0.73	0.72	0.71	0.75	0.72	0.000	0.000	1.000	0.120	0.002	1.000	0.968	1.000	1.000	0.412
Carbon fixation pathways in prokaryotes	1.07	0.98	0.87	1.10	1.02	0.91	0.517	0.000	0.258	0.444	0.000	0.253	1.000	1.000	1.000	1.000
Methane metabolism	1.35	1.43	1.42	1.28	1.34	1.44	0.150	0.029	1.000	0.681	0.004	0.275	1.000	1.000	1.000	1.000
Nitrogen metabolism	0.69	0.68	0.66	0.68	0.68	0.66	0.283	0.065	1.000	1.000	0.124	0.725	1.000	1.000	1.000	1.000
Oxidative phosphorylation	1.15	1.06	0.99	1.18	1.06	1.02	0.828	0.342	1.000	0.503	0.330	1.000	1.000	1.000	1.000	1.000
Photosynthesis	0.35	0.41	0.41	0.35	0.39	0.42	0.034	0.003	1.000	0.444	0.001	0.402	1.000	1.000	1.000	1.000
Photosynthesis - anten1 proteins	0.00	0.00	0.00	0.00	0.00	0.00	0.003	0.000	1.000	0.363	0.001	0.663	1.000	1.000	1.000	1.000
Photosynthesis proteins	0.36	0.41	0.41	0.35	0.39	0.42	0.037	0.003	1.000	0.444	0.002	0.406	1.000	1.000	1.000	1.000
Sulfur metabolism	0.25	0.24	0.23	0.25	0.24	0.23	0.830	0.701	1.000	1.000	1.000	1.000	1.000	1.000	1.000	1.000
Cytochrome P450	0.00	0.00	0.00	0.00	0.00	0.00	0.863	0.761	1.000	1.000	1.000	1.000	1.000	1.000	1.000	1.000
Peptidases	1.90	2.00	2.01	2.00	2.01	1.99	0.000	0.000	0.563	0.444	0.000	0.217	0.564	1.000	1.000	0.478
Protein ki1ses	0.24	0.23	0.23	0.22	0.20	0.23	1.000	0.428	1.000	1.000	0.016	0.060	0.564	0.686	1.000	1.000
Glycosaminoglycan biosynthesis - chondroitin sulfate	0.00	0.00	0.00	0.00	0.00	0.00	1.000	1.000	1.000	1.000	1.000	1.000	1.000	1.000	1.000	1.000
Glycosaminoglycan degradation	0.08	0.09	0.12	0.08	0.07	0.11	1.000	0.086	0.574	1.000	0.207	0.273	1.000	1.000	1.000	1.000
Glycosphingolipid biosynthesis - ganglio series	0.06	0.05	0.05	0.06	0.05	0.05	0.828	1.000	1.000	0.653	0.445	1.000	1.000	1.000	1.000	1.000
Glycosphingolipid biosynthesis - globo series	0.12	0.10	0.10	0.12	0.10	0.10	0.830	1.000	1.000	0.505	0.453	1.000	1.000	1.000	1.000	1.000
Glycosphingolipid biosynthesis - lacto and neolacto series	0.00	0.00	0.00	0.00	0.00	0.00	0.000	0.000	1.000	0.028	0.002	1.000	1.000	1.000	1.000	0.374
Glycosylphosphatidylinositol(GPI)-anchor biosynthesis	0.00	0.00	0.00	0.00	0.00	0.00	1.000	1.000	1.000	1.000	1.000	1.000	1.000	1.000	1.000	1.000
Glycosyltransferases	0.35	0.29	0.23	0.37	0.33	0.25	0.090	0.000	0.258	0.573	0.000	0.043	1.000	1.000	1.000	1.000
Lipopolysaccharide biosynthesis	0.32	0.26	0.14	0.38	0.30	0.19	0.497	0.000	0.258	0.827	0.001	0.076	1.000	1.000	1.000	1.000
Lipopolysaccharide biosynthesis proteins	0.42	0.37	0.26	0.47	0.40	0.31	0.502	0.001	0.452	0.835	0.004	0.207	1.000	1.000	1.000	1.000
N-Glycan biosynthesis	0.03	0.03	0.02	0.04	0.03	0.02	0.786	0.005	0.529	0.997	0.016	0.275	1.000	1.000	1.000	1.000
Other glycan degradation	0.30	0.22	0.21	0.31	0.24	0.21	0.068	0.029	1.000	0.285	0.007	0.995	1.000	1.000	1.000	1.000
Other types of O-glycan biosynthesis	0.00	0.00	0.00	0.00	0.00	0.00	1.000	1.000	1.000	1.000	1.000	1.000	1.000	1.000	1.000	1.000
Peptidoglycan biosynthesis	0.90	0.89	0.86	0.96	0.91	0.86	0.251	0.111	1.000	1.000	0.850	1.000	0.854	1.000	1.000	1.000
Various types of N-glycan biosynthesis	0.00	0.00	0.00	0.00	0.00	0.00	0.828	0.853	1.000	0.645	0.073	0.912	0.564	1.000	1.000	1.000
alpha-Linolenic acid metabolism	0.00	0.00	0.00	0.00	0.00	0.00	0.001	0.000	1.000	1.000	0.016	0.107	1.000	0.443	1.000	1.000
Arachidonic acid metabolism	0.04	0.04	0.03	0.05	0.05	0.03	1.000	0.054	0.529	1.000	0.015	0.103	0.907	1.000	1.000	1.000
Biosynthesis of unsaturated fatty acids	0.12	0.11	0.10	0.12	0.11	0.10	0.801	1.000	1.000	0.997	0.381	1.000	1.000	1.000	1.000	1.000
Ether lipid metabolism	0.00	0.00	0.00	0.00	0.00	0.00	0.000	0.000	1.000	0.252	0.031	1.000	0.564	1.000	1.000	0.145
Fatty acid biosynthesis	0.49	0.48	0.50	0.49	0.49	0.48	0.497	0.005	0.574	0.997	0.104	0.720	1.000	1.000	1.000	1.000
Fatty acid elongation in mitochondria	0.00	0.00	0.00	0.00	0.00	0.00	0.873	0.853	1.000	1.000	1.000	1.000	1.000	1.000	1.000	1.000
Fatty acid metabolism	0.25	0.19	0.17	0.23	0.20	0.18	0.000	0.000	1.000	0.444	0.004	0.693	0.787	1.000	1.000	0.770
Glycerolipid metabolism	0.36	0.36	0.39	0.34	0.37	0.38	0.448	0.008	0.695	0.444	0.001	0.508	0.907	1.000	1.000	1.000
Glycerophospholipid metabolism	0.52	0.56	0.58	0.49	0.53	0.57	0.002	0.000	0.563	0.111	0.000	0.007	0.698	0.686	1.000	1.000
Linoleic acid metabolism	0.06	0.06	0.06	0.07	0.08	0.06	0.828	0.169	1.000	0.772	0.618	0.305	0.907	1.000	1.000	1.000
Lipid biosynthesis proteins	0.63	0.56	0.53	0.66	0.60	0.54	0.382	0.082	1.000	0.473	0.002	0.354	0.955	1.000	1.000	1.000
Primary bile acid biosynthesis	0.02	0.02	0.03	0.02	0.02	0.03	1.000	0.065	0.574	1.000	0.020	0.076	0.955	1.000	1.000	1.000
Secondary bile acid biosynthesis	0.02	0.02	0.03	0.02	0.02	0.03	0.830	0.036	0.574	1.000	0.018	0.089	0.968	1.000	1.000	1.000
Sphingolipid metabolism	0.20	0.14	0.12	0.20	0.15	0.12	0.002	0.000	1.000	0.133	0.000	0.626	1.000	1.000	1.000	1.000
Steroid biosynthesis	0.00	0.00	0.00	0.00	0.00	0.00	0.558	0.153	1.000	1.000	0.073	0.474	1.000	1.000	1.000	1.000
Steroid hormone biosynthesis	0.01	0.00	0.00	0.01	0.00	0.00	0.014	0.003	1.000	0.653	0.866	0.912	0.568	1.000	1.000	0.478
Synthesis and degradation of ketone bodies	0.04	0.03	0.03	0.02	0.03	0.03	0.503	0.089	1.000	0.630	0.497	1.000	0.498	1.000	1.000	1.000
Biotin metabolism	0.15	0.15	0.14	0.14	0.15	0.14	0.585	0.875	1.000	0.509	0.183	1.000	0.968	1.000	1.000	1.000
Folate biosynthesis	0.41	0.34	0.29	0.43	0.40	0.31	0.032	0.000	0.491	0.966	0.000	0.007	1.000	0.686	1.000	1.000
Lipoic acid metabolism	0.05	0.01	0.01	0.04	0.02	0.01	0.000	0.000	0.548	0.444	0.000	0.076	0.955	0.319	1.000	0.478
Nicoti1te and nicoti1mide metabolism	0.50	0.50	0.48	0.55	0.53	0.49	0.036	0.021	1.000	1.000	0.689	0.805	0.439	1.000	1.000	0.478
One carbon pool by folate	0.69	0.70	0.69	0.75	0.72	0.69	0.013	0.001	1.000	1.000	1.000	1.000	0.439	1.000	1.000	1.000
Pantothe1te and CoA biosynthesis	0.65	0.60	0.55	0.68	0.63	0.57	0.430	0.001	0.529	0.444	0.000	0.216	0.854	1.000	1.000	1.000
Porphyrin and chlorophyll metabolism	0.70	0.74	0.63	0.67	0.66	0.66	0.517	0.853	0.695	1.000	0.678	1.000	1.000	1.000	1.000	1.000
Retinol metabolism	0.03	0.02	0.01	0.04	0.03	0.01	0.007	0.000	0.548	0.444	0.000	0.010	0.854	0.443	1.000	1.000
Riboflavin metabolism	0.24	0.22	0.17	0.25	0.22	0.19	0.802	0.001	0.297	0.778	0.030	0.626	1.000	1.000	1.000	1.000
Thiamine metabolism	0.47	0.52	0.53	0.45	0.47	0.53	0.000	0.000	0.554	0.061	0.000	0.000	0.714	0.001	1.000	1.000
Ubiquinone and other terpenoid-quinone biosynthesis	0.22	0.17	0.11	0.26	0.22	0.14	0.298	0.000	0.258	0.827	0.000	0.054	0.955	1.000	1.000	1.000
Vitamin B6 metabolism	0.23	0.20	0.17	0.25	0.23	0.18	0.520	0.003	0.548	0.752	0.000	0.011	0.564	0.686	1.000	1.000
beta-Alanine metabolism	0.19	0.16	0.14	0.19	0.17	0.15	0.208	0.023	1.000	0.778	0.046	0.693	1.000	1.000	1.000	1.000
Cyanoamino acid metabolism	0.31	0.30	0.33	0.34	0.32	0.32	0.692	0.011	0.563	0.997	1.000	0.912	0.787	1.000	1.000	1.000
D-Alanine metabolism	0.12	0.11	0.11	0.11	0.13	0.11	0.802	0.449	1.000	0.257	0.431	0.607	1.000	1.000	1.000	1.000
D-Arginine and D-ornithine metabolism	0.00	0.00	0.00	0.00	0.00	0.00	0.497	0.805	1.000	0.997	0.124	0.794	0.968	1.000	1.000	1.000

D-Glutamine and D-glutamate metabolism	0.17	0.16	0.15	0.17	0.17	0.15	0.089	0.089	1.000	0.466	0.340	1.000	1.000	1.000	1.000	1.000
Glutathione metabolism	0.21	0.20	0.17	0.20	0.21	0.18	0.830	0.023	0.563	0.966	0.835	0.626	1.000	1.000	1.000	1.000
Phospho1te and phosphi1te metabolism	0.06	0.06	0.08	0.06	0.06	0.08	0.679	0.005	0.529	0.444	0.008	0.723	1.000	1.000	1.000	1.000
Selenocompound metabolism	0.37	0.41	0.43	0.36	0.42	0.42	0.001	0.000	0.529	0.015	0.000	0.342	1.000	1.000	1.000	1.000
Taurine and hypotaurine metabolism	0.12	0.10	0.10	0.12	0.11	0.10	0.395	0.088	1.000	0.550	0.051	0.961	1.000	1.000	1.000	1.000
Biosynthesis of 12-, 14- and 16-membered macrolides	0.00	0.00	0.00	0.00	0.00	0.00	1.000	1.000	1.000	1.000	1.000	1.000	1.000	1.000	1.000	1.000
Biosynthesis of ansamycins	0.10	0.12	0.12	0.10	0.11	0.12	0.002	0.000	1.000	0.178	0.000	0.491	1.000	1.000	1.000	1.000
Biosynthesis of siderophore group nonribosomal peptides	0.02	0.02	0.01	0.02	0.03	0.02	0.828	0.531	0.754	0.997	0.089	0.076	0.854	1.000	1.000	0.624
Biosynthesis of type II polyketide backbone	0.00	0.00	0.00	0.00	0.00	0.00	1.000	1.000	1.000	1.000	1.000	1.000	1.000	1.000	1.000	1.000
Biosynthesis of type II polyketide products	0.00	0.00	0.00	0.00	0.00	0.00	0.072	0.191	1.000	0.592	0.278	1.000	1.000	1.000	1.000	1.000
Biosynthesis of vancomycin group antibiotics	0.07	0.07	0.06	0.07	0.07	0.06	0.873	1.000	1.000	0.444	0.895	0.508	1.000	1.000	1.000	1.000
Carotenoid biosynthesis	0.01	0.00	0.00	0.01	0.00	0.00	0.000	0.000	1.000	0.444	0.000	0.005	1.000	0.000	1.000	1.000
Geraniol degradation	0.07	0.03	0.01	0.07	0.04	0.02	0.000	0.000	0.258	0.444	0.000	0.007	1.000	0.686	1.000	1.000
Limonene and pinene degradation	0.11	0.08	0.09	0.11	0.09	0.09	0.004	0.254	0.548	0.444	0.381	1.000	1.000	1.000	1.000	0.412
Polyketide sugar unit biosynthesis	0.23	0.22	0.21	0.23	0.24	0.21	0.517	0.330	1.000	0.550	0.730	0.911	1.000	1.000	1.000	1.000
Prenyltransferases	0.36	0.34	0.31	0.39	0.36	0.32	1.000	0.252	0.675	0.666	0.012	0.485	0.714	1.000	1.000	1.000
Sesquiterpenoid biosynthesis	0.00	0.00	0.00	0.00	0.00	0.00	1.000	1.000	1.000	1.000	1.000	1.000	1.000	1.000	1.000	1.000
Terpenoid backbone biosynthesis	0.64	0.65	0.63	0.66	0.67	0.63	0.000	0.000	1.000	0.444	0.073	1.000	0.854	1.000	1.000	0.164
Tetracycline biosynthesis	0.12	0.13	0.16	0.11	0.12	0.15	0.627	0.001	0.497	0.560	0.000	0.090	0.854	1.000	1.000	1.000
Zeatin biosynthesis	0.06	0.06	0.05	0.07	0.06	0.05	1.000	0.384	1.000	0.545	0.001	0.262	0.564	1.000	1.000	1.000
Purine metabolism	2.42	2.43	2.38	2.45	2.52	2.41	0.004	0.000	1.000	0.126	0.001	1.000	1.000	1.000	1.000	1.000
Pyrimidine metabolism	2.07	2.06	2.02	2.11	2.09	2.04	0.007	0.000	1.000	0.590	0.002	0.305	1.000	1.000	1.000	1.000
1,1,1-Trichloro-2,2-bis(4-chlorophenyl)ethane (DDT) degradation	0.00	0.00	0.00	0.00	0.00	0.00	0.007	0.000	1.000	0.630	0.026	0.693	1.000	1.000	1.000	1.000
Aminobenzoate degradation	0.14	0.11	0.11	0.13	0.12	0.10	0.008	0.007	1.000	1.000	0.153	0.602	0.787	1.000	1.000	1.000
Atrazine degradation	0.01	0.01	0.01	0.01	0.01	0.01	0.323	0.010	1.000	0.997	0.689	1.000	0.854	1.000	1.000	1.000
Benzoate degradation	0.24	0.23	0.25	0.21	0.23	0.24	1.000	0.086	0.563	0.466	0.007	0.634	0.564	1.000	1.000	1.000
Bisphenol degradation	0.07	0.07	0.08	0.08	0.08	0.08	0.513	0.001	0.529	0.653	0.276	1.000	1.000	1.000	1.000	1.000
Caprolactam degradation	0.03	0.02	0.01	0.02	0.03	0.02	0.030	0.000	1.000	0.963	0.494	0.342	0.714	1.000	1.000	1.000
Chloroalkane and chloroalkene degradation	0.18	0.16	0.17	0.17	0.17	0.17	0.898	0.419	0.777	1.000	0.381	1.000	1.000	1.000	1.000	1.000
Chlorocyclohexane and chlorobenzene degradation	0.01	0.00	0.00	0.01	0.00	0.00	0.000	0.000	1.000	0.203	0.000	0.693	0.907	1.000	1.000	1.000
Dioxin degradation	0.06	0.05	0.07	0.05	0.06	0.06	1.000	0.130	0.548	0.505	0.026	0.873	0.854	1.000	1.000	1.000
Drug metabolism - cytochrome P450	0.04	0.01	0.01	0.03	0.02	0.01	0.000	0.000	0.529	0.444	0.000	0.076	1.000	0.686	1.000	1.000
Drug metabolism - other enzymes	0.35	0.36	0.34	0.37	0.36	0.35	0.009	0.016	1.000	1.000	0.870	1.000	0.714	1.000	1.000	1.000
Ethylbenzene degradation	0.06	0.05	0.05	0.07	0.06	0.05	0.139	0.227	1.000	0.424	0.000	0.076	0.439	0.443	1.000	1.000
Fluorobenzoate degradation	0.00	0.00	0.00	0.00	0.00	0.00	0.000	0.000	1.000	0.444	0.000	0.014	1.000	0.443	1.000	1.000
Metabolism of xenobiotics by cytochrome P450	0.04	0.01	0.01	0.03	0.02	0.01	0.000	0.000	0.529	0.444	0.000	0.078	1.000	0.686	1.000	1.000
1phthalene degradation	0.17	0.13	0.14	0.19	0.15	0.14	0.090	0.853	0.574	0.444	0.022	0.912	0.907	1.000	1.000	1.000
Nitrotoluene degradation	0.05	0.06	0.06	0.04	0.05	0.06	0.692	0.566	1.000	0.586	0.008	0.508	0.714	1.000	1.000	1.000
Polycyclic aromatic hydrocarbon degradation	0.12	0.12	0.14	0.11	0.12	0.13	0.325	0.000	0.258	0.778	0.000	0.076	1.000	1.000	1.000	1.000
Styrene degradation	0.02	0.02	0.02	0.01	0.03	0.02	0.325	0.640	1.000	0.017	0.183	0.245	0.968	1.000	1.000	0.478
Toluene degradation	0.10	0.08	0.05	0.11	0.09	0.06	0.119	0.000	0.258	0.848	0.000	0.054	1.000	1.000	1.000	1.000
Xylene degradation	0.05	0.05	0.07	0.05	0.06	0.06	0.540	0.007	0.574	0.397	0.024	1.000	1.000	1.000	1.000	1.000
Cardiac muscle contraction	0.01	0.00	0.00	0.00	0.00	0.00	0.000	0.000	1.000	0.003	0.000	0.626	0.968	1.000	1.000	0.145
Vascular smooth muscle contraction	0.00	0.00	0.00	0.00	0.00	0.00	1.000	1.000	1.000	1.000	1.000	1.000	1.000	1.000	1.000	1.000
Bile secretion	0.00	0.00	0.00	0.00	0.00	0.00	0.553	0.300	1.000	0.457	0.026	0.914	0.955	1.000	1.000	1.000
Carbohydrate digestion and absorption	0.03	0.02	0.02	0.04	0.03	0.02	0.854	0.116	1.000	1.000	0.005	0.054	0.854	1.000	1.000	1.000
Fat digestion and absorption	0.00	0.00	0.00	0.00	0.00	0.00	1.000	1.000	1.000	1.000	1.000	1.000	1.000	1.000	1.000	1.000
Gastric acid secretion	0.00	0.00	0.00	0.00	0.00	0.00	1.000	1.000	1.000	1.000	1.000	1.000	1.000	1.000	1.000	1.000
Mineral absorption	0.01	0.02	0.01	0.01	0.01	0.01	0.020	0.365	1.000	0.772	0.011	0.360	0.968	0.651	1.000	1.000
Pancreatic secretion	0.00	0.00	0.00	0.00	0.00	0.00	1.000	1.000	1.000	1.000	1.000	1.000	1.000	1.000	1.000	1.000
Protein digestion and absorption	0.03	0.02	0.01	0.04	0.03	0.02	0.266	0.000	0.297	0.482	0.000	0.060	0.895	1.000	1.000	1.000
Salivary secretion	0.00	0.00	0.00	0.00	0.00	0.00	1.000	1.000	1.000	1.000	1.000	1.000	1.000	1.000	1.000	1.000
Adipocytokine sig1ling pathway	0.09	0.05	0.03	0.10	0.06	0.03	0.000	0.000	0.258	0.057	0.000	0.005	0.907	0.709	1.000	1.000
GnRH sig1ling pathway	0.00	0.00	0.00	0.00	0.00	0.00	1.000	1.000	1.000	1.000	1.000	0.005	1.000	0.000	1.000	1.000
Insulin sig1ling pathway	0.08	0.08	0.08	0.09	0.10	0.08	0.623	0.531	1.000	0.592	0.961	0.386	1.000	1.000	1.000	1.000
Melanogenesis	0.00	0.00	0.00	0.00	0.00	0.00	1.000	1.000	1.000	1.000	1.000	1.000	1.000	1.000	1.000	1.000
PPAR sig1ling pathway	0.12	0.08	0.05	0.13	0.10	0.06	0.000	0.000	0.258	0.158	0.000	0.005	0.968	0.686	1.000	1.000
Progesterone-mediated oocyte maturation	0.04	0.05	0.05	0.05	0.05	0.05	0.008	0.001	1.000	1.000	1.000	1.000	0.439	1.000	1.000	0.478
Renin-angiotensin system	0.00	0.00	0.00	0.00	0.00	0.00	0.000	0.000	1.000	0.017	0.000	0.253	1.000	1.000	1.000	0.640
Circadian rhythm - plant	0.00	0.00	0.00	0.00	0.00	0.00	0.000	0.000	1.000	0.653	0.023	0.626	1.000	1.000	1.000	0.617
Plant-pathogen interaction	0.15	0.15	0.14	0.15	0.15	0.15	0.027	0.057	1.000	0.444	0.047	1.000	1.000	1.000	1.000	1.000
Aldosterone-regulated sodium reabsorption	0.00	0.00	0.00	0.00	0.00	0.00	1.000	1.000	1.000	1.000	1.000	1.000	1.000	1.000	1.000	1.000
Endocrine and other factor-regulated calcium reabsorption	0.00	0.00	0.00	0.00	0.00	0.00	1.000	1.000	1.000	1.000	1.000	1.000	1.000	1.000	1.000	1.000
Proximal tubule bicarbo1te reclamation	0.02	0.01	0.00	0.02	0.01	0.00	0.000	0.000	1.000	0.444	0.000	0.005	1.000	0.443	1.000	1.000
Vasopressin-regulated water reabsorption	0.00	0.00	0.00	0.00	0.00	0.00	1.000	1.000	1.000	1.000	1.000	1.000	1.000	1.000	1.000	1.000
Antigen processing and presentation	0.04	0.05	0.05	0.05	0.05	0.05	0.008	0.001	1.000	1.000	1.000	1.000	0.439	1.000	1.000	0.478
Complement and coagulation cascades	0.00	0.00	0.00	0.00	0.00	0.00	1.000	1.000	1.000	1.000	1.000	1.000	1.000	1.000	1.000	1.000
Cytosolic D1-sensing pathway	0.00	0.00	0.00	0.00	0.00	0.00	1.000	1.000	1.000	1.000	1.000	1.000	1.000	1.000	1.000	1.000
Fc epsilon RI sig1ling pathway	0.00	0.00	0.00	0.00	0.00	0.00	1.000	1.000	1.000	1.000	1.000	1.000	1.000	1.000	1.000	1.000
Fc gamma R-mediated phagocytosis	0.00	0.00	0.00	0.00	0.00	0.00	1.000	1.000	1.000	1.000	1.000	0.005	1.000	0.000	1.000	1.000
Hematopoietic cell lineage	0.00	0.00	0.00	0.00	0.00	0.00	1.000	1.000	1.000	1.000	1.000	1.000	1.000	1.000	1.000	1.000
Leukocyte transendothelial migration	0.00	0.00	0.00	0.00	0.00	0.00	1.000	1.000	1.000	1.000	1.000	1.000	1.000	1.000	1.000	1.000
NOD-like receptor sig1ling pathway	0.05	0.05	0.05	0.05	0.05	0.05	0.073	0.011	1.000	1.000	0.350	0.663	0.439	1.000	1.000	0.519
RIG-I-like receptor sig1ling pathway	0.00	0.00	0.00	0.00	0.00	0.00	0.000	0.000	1.000	0.203	0.014	1.000	0.564	1.000	1.000	0.006

Cholinergic sy1pse	0.00	0.00	0.00	0.00	0.00	0.00	1.000	1.000	1.000	1.000	1.000	1.000	1.000	1.000	1.000	1.000
Glutamatergic sy1pse	0.10	0.10	0.10	0.11	0.10	0.10	0.304	0.031	1.000	1.000	0.850	1.000	0.968	1.000	1.000	1.000
Long-term depression	0.00	0.00	0.00	0.00	0.00	0.00	1.000	1.000	1.000	1.000	1.000	1.000	1.000	1.000	1.000	1.000
Long-term potentiation	0.00	0.00	0.00	0.00	0.00	0.00	1.000	1.000	1.000	1.000	1.000	1.000	1.000	1.000	1.000	1.000
Neurotrophin sig1ling pathway	0.00	0.00	0.00	0.00	0.00	0.00	1.000	1.000	1.000	1.000	1.000	1.000	1.000	1.000	1.000	1.000
Olfactory transduction	0.00	0.00	0.00	0.00	0.00	0.00	1.000	1.000	1.000	1.000	1.000	1.000	1.000	1.000	1.000	1.000
Phototransduction	0.00	0.00	0.00	0.00	0.00	0.00	1.000	1.000	1.000	1.000	1.000	1.000	1.000	1.000	1.000	1.000
Phototransduction - fly	0.00	0.00	0.00	0.00	0.00	0.00	1.000	1.000	1.000	1.000	1.000	1.000	1.000	1.000	1.000	1.000
Cell division	0.07	0.06	0.06	0.07	0.07	0.06	0.085	0.001	1.000	1.000	0.051	0.344	1.000	1.000	1.000	1.000
Cell motility and secretion	0.18	0.16	0.13	0.19	0.18	0.15	0.468	0.000	0.356	1.000	0.014	0.131	1.000	1.000	1.000	1.000
Electron transfer carriers	0.01	0.01	0.01	0.02	0.01	0.01	0.679	0.065	1.000	1.000	0.236	0.693	1.000	1.000	1.000	1.000
Germi1tion	0.02	0.01	0.01	0.02	0.01	0.01	0.000	0.000	1.000	0.444	0.000	0.286	1.000	0.686	1.000	1.000
Inorganic ion transport and metabolism	0.18	0.15	0.15	0.18	0.15	0.15	0.004	0.086	0.785	0.444	0.325	1.000	0.955	1.000	1.000	0.412
Membrane and intracellular structural molecules	0.62	0.50	0.40	0.71	0.58	0.44	0.090	0.000	0.529	0.444	0.000	0.076	0.907	1.000	1.000	1.000
Other ion-coupled transporters	1.08	1.14	1.08	1.11	1.05	1.10	0.003	0.005	1.000	1.000	0.024	0.054	1.000	0.178	1.000	1.000
Other transporters	0.25	0.28	0.28	0.27	0.28	0.28	0.000	0.000	1.000	0.444	0.002	0.480	0.955	1.000	1.000	1.000
Pores ion channels	0.36	0.29	0.22	0.38	0.32	0.25	0.106	0.000	0.258	0.444	0.000	0.107	1.000	1.000	1.000	1.000
Sig1l transduction mechanisms	0.41	0.45	0.49	0.41	0.42	0.47	0.002	0.000	0.258	0.586	0.000	0.007	1.000	0.686	1.000	1.000
Sporulation	0.43	0.55	0.71	0.46	0.40	0.63	0.058	0.000	0.833	1.000	0.015	0.081	1.000	1.000	1.000	1.000
Protein folding and associated processing	0.67	0.67	0.63	0.67	0.69	0.65	0.104	0.355	1.000	0.158	0.020	1.000	1.000	1.000	1.000	1.000
Replication, recombi1tion and repair proteins	0.71	0.83	0.98	0.69	0.74	0.94	0.008	0.000	0.258	0.503	0.000	0.005	1.000	1.000	1.000	1.000
Restriction enzyme	0.18	0.16	0.16	0.20	0.17	0.16	0.873	0.853	1.000	0.203	0.030	1.000	0.698	1.000	1.000	1.000
Transcription related proteins	0.01	0.02	0.01	0.01	0.01	0.02	0.411	0.440	1.000	0.444	0.007	0.796	0.854	1.000	1.000	1.000
Translation proteins	0.98	1.02	1.00	1.00	1.01	1.01	0.000	0.000	1.000	0.424	0.000	0.076	1.000	1.000	1.000	1.000
Amino acid metabolism	0.19	0.17	0.17	0.20	0.17	0.17	0.599	0.976	1.000	0.444	0.225	1.000	1.000	1.000	1.000	1.000
Biosynthesis and biodegradation of secondary metabolites	0.06	0.05	0.04	0.06	0.05	0.04	0.553	0.008	0.574	0.444	0.002	0.626	0.968	1.000	1.000	1.000
Carbohydrate metabolism	0.18	0.15	0.13	0.20	0.16	0.13	0.712	0.080	1.000	0.509	0.005	0.490	0.907	1.000	1.000	1.000
Energy metabolism	0.93	0.85	0.80	1.02	0.89	0.82	0.828	0.153	1.000	0.023	0.000	0.725	0.439	1.000	1.000	1.000
Glycan biosynthesis and metabolism	0.04	0.04	0.02	0.05	0.05	0.03	0.898	0.000	0.258	1.000	0.007	0.034	0.955	1.000	1.000	1.000
Lipid metabolism	0.13	0.13	0.12	0.15	0.15	0.12	0.830	0.640	1.000	1.000	0.403	0.384	0.854	1.000	1.000	1.000
Metabolism of cofactors and vitamins	0.10	0.10	0.08	0.10	0.10	0.09	1.000	0.063	0.529	1.000	0.347	0.607	1.000	1.000	1.000	1.000
Nucleotide metabolism	0.04	0.04	0.04	0.02	0.03	0.04	0.372	0.095	1.000	0.732	0.000	0.071	0.564	0.686	1.000	1.000
Others	0.82	0.86	1.01	0.79	0.74	0.97	0.411	0.000	0.258	1.000	0.000	0.005	1.000	0.686	1.000	0.942
Function unknown	1.29	1.30	1.29	1.24	1.34	1.30	0.116	0.011	1.000	0.028	0.000	0.796	0.714	1.000	1.000	1.000
General function prediction only	3.65	3.89	4.03	3.64	3.76	3.98	0.000	0.000	0.258	0.217	0.000	0.005	1.000	0.443	1.000	1.000

Table S5. Faeces KEGG L2

KEGG pathway	Relative abundance: Early-weaned Wk5	Relative abundance: Early-weaned Wk7	Relative abundance: Early-weaned Wk9	Relative abundance: Late-weaned Wk5	Relative abundance: Late-weaned Wk7	Relative abundance: Late-weaned Wk9	P-values: Early-weaned Wk 5 vs Wk 7	P-values: Early-weaned Wk 5 vs Wk 9	P-values: Early-weaned Wk 7 vs Wk 9	P-values: Late-weaned Wk 5vs Wk 7	P-values: Late-weaned Wk 5 vs Wk 9	P-values: Late-weaned Wk 7 vs Wk 9	P-values: Early vs Late Wk 5	P-values: Early vs Late Wk 7	P-values: Early vs Late Wk 9	P-values: (Early Wk7-Wk 5) vs (Late Wk9-Wk7)
Cellular Processes																
Cell Growth and Death	0.53	0.56	0.55	0.52	0.53	0.54	0.479	0.651	0.975	0.933	0.541	0.997	0.996	0.277	0.998	0.988
Cell Motility	1.08	1.80	2.00	1.06	1.11	1.79	0.002	0.000	0.975	0.933	0.002	0.009	0.996	0.002	0.998	0.988
Transport and Catabolism	0.30	0.35	0.36	0.32	0.34	0.35	0.295	0.182	0.975	0.933	0.541	0.997	0.996	0.838	0.998	0.988
Environmental Information Processing																
Membrane Transport	12.65	10.61	10.66	12.40	11.86	10.83	0.002	0.003	0.975	0.933	0.062	0.276	0.996	0.160	0.998	0.988
Signal Transduction	1.33	1.38	1.43	1.36	1.33	1.42	0.752	0.201	0.975	0.933	0.605	0.353	0.996	0.628	0.998	0.988
Signaling Molecules and Interaction	0.20	0.19	0.19	0.19	0.20	0.19	0.083	0.032	0.975	0.933	0.381	0.759	0.996	0.288	0.998	0.988
Genetic Information Processing																
Folding, Sorting and Degradation	2.47	2.63	2.62	2.47	2.54	2.62	0.083	0.099	0.975	0.933	0.084	0.453	0.996	0.410	0.998	0.988
Replication and Repair	9.09	9.65	9.50	9.03	9.08	9.52	0.205	0.477	0.975	0.933	0.196	0.276	0.996	0.113	0.998	0.988
Transcription	2.93	2.69	2.68	2.92	2.84	2.69	0.014	0.014	0.975	0.933	0.059	0.276	0.996	0.196	0.998	0.988
Translation	5.74	6.16	6.13	5.67	5.78	6.13	0.201	0.182	0.975	0.933	0.078	0.297	0.996	0.196	0.998	0.988
Human Diseases																
Cancers	0.09	0.11	0.12	0.09	0.10	0.11	0.023	0.002	0.975	0.933	0.002	0.276	0.996	0.277	0.998	0.988
Infectious Diseases	0.38	0.39	0.38	0.38	0.38	0.39	0.843	0.325	0.975	0.933	0.541	1.000	0.996	0.838	0.998	0.988
Metabolic Diseases	0.10	0.11	0.11	0.10	0.10	0.11	0.014	0.002	0.975	0.933	0.084	0.453	0.996	0.196	0.998	0.988
Neurodegenerative Diseases	0.09	0.10	0.10	0.08	0.09	0.10	0.595	0.264	0.975	0.933	0.094	0.353	0.996	0.454	0.998	0.988
Metabolism																
Amino Acid Metabolism	9.75	9.72	9.74	9.86	9.75	9.66	0.059	0.094	0.975	0.933	0.036	0.287	0.996	0.226	0.998	0.988
Biosynthesis of Other Secondary Metabolites	0.98	0.99	1.00	1.00	1.02	0.99	0.423	0.512	0.975	0.933	0.237	0.276	0.996	0.146	0.998	0.988
Carbohydrate Metabolism	11.25	10.69	10.67	11.29	11.30	10.80	0.000	0.000	0.975	0.933	0.002	0.009	0.996	0.000	0.998	0.988
Energy Metabolism	6.00	5.97	6.05	6.01	6.10	6.01	0.083	0.354	0.975	0.933	0.344	0.276	0.996	0.036	0.998	0.988
Enzyme Families	2.19	2.20	2.20	2.19	2.19	2.19	0.083	0.099	0.975	0.933	0.176	0.675	0.996	0.410	0.998	0.988
Glycan Biosynthesis and Metabolism	2.27	2.65	2.60	2.35	2.48	2.59	0.128	0.182	0.975	0.933	0.379	0.759	0.996	0.542	0.998	0.988
Lipid Metabolism	2.73	2.75	2.77	2.72	2.75	2.77	0.201	0.429	0.975	0.933	0.541	0.759	0.996	0.288	0.998	0.988
Metabolism of Cofactors and Vitamins	4.36	4.46	4.39	4.42	4.43	4.39	0.447	0.094	0.975	0.933	0.059	0.276	0.996	0.277	0.998	0.988
Metabolism of Other Amino Acids	1.44	1.50	1.49	1.44	1.49	1.51	0.752	0.902	0.975	0.933	0.238	0.997	0.996	0.357	0.998	0.988
Metabolism of Terpenoids and Polyketides	1.61	1.68	1.69	1.61	1.63	1.68	0.479	0.185	0.975	0.933	0.344	0.383	0.996	0.357	0.998	0.988
Nucleotide Metabolism	4.18	4.30	4.25	4.14	4.16	4.29	0.665	0.358	0.975	0.933	0.605	0.703	0.996	0.542	0.998	0.988
Xenobiotics Biodegradation and Metabolism	1.61	1.55	1.58	1.62	1.61	1.63	0.002	0.052	0.975	0.933	0.421	0.997	0.996	0.036	0.998	0.988

Organismal Systems																
Endocrine System	0.29	0.35	0.35	0.30	0.31	0.34	0.002	0.002	0.975	0.933	0.177	0.276	0.996	0.146	0.998	0.988
Environmental Adaptation	0.14	0.15	0.15	0.14	0.14	0.15	0.201	0.073	0.975	0.933	0.059	0.276	0.996	0.196	0.998	0.988
Immune System	0.09	0.10	0.10	0.09	0.09	0.10	0.083	0.026	0.975	0.933	0.068	0.759	0.996	0.361	0.998	0.988
Nervous System	0.11	0.11	0.11	0.12	0.12	0.11	0.479	0.651	0.975	0.933	0.143	0.059	0.996	0.037	0.998	0.988
Unclassified																
Cellular Processes and Signaling	3.97	4.00	3.95	4.04	4.05	3.96	0.340	0.150	0.975	0.933	0.068	0.276	0.996	0.196	0.998	0.988
Genetic Information Processing	2.63	2.67	2.66	2.63	2.60	2.64	0.479	0.525	0.975	0.933	0.421	0.997	0.996	0.653	0.998	0.988
Metabolism	2.41	2.43	2.41	2.43	2.42	2.40	0.330	0.182	0.975	0.933	0.094	0.453	0.996	0.410	0.998	0.988
Poorly Characterized	4.90	4.88	4.89	4.90	4.99	4.89	0.001	0.002	0.975	0.933	0.035	0.009	0.996	0.000	0.998	0.988
Human Diseases																
Cardiovascular Diseases	0.00	0.00	0.00	0.00	0.00	0.00	0.139	0.150	0.975	0.933	1.000	1.000	0.996	0.196	0.998	0.988
Immune System Diseases	0.05	0.05	0.04	0.04	0.04	0.04	0.265	0.018	0.975	0.933	0.605	1.000	0.996	0.357	0.998	0.988
Organismal Systems																
Circulatory System	0.00	0.00	0.00	0.00	0.00	0.00	0.245	0.182	0.975	0.933	1.000	1.000	0.996	0.196	0.998	0.988
Digestive System	0.04	0.06	0.05	0.04	0.04	0.05	0.014	0.094	0.975	0.933	0.094	0.466	0.996	0.196	0.998	0.988
Excretory System	0.01	0.02	0.03	0.02	0.02	0.02	0.010	0.002	0.975	0.933	0.078	1.000	0.996	0.357	0.998	0.988

Table S6. Faecal KEGG L3

KEGG pathway	Early-wean			Late-wean (L)			P-values									
	Wk5	Wk7	Wk9	Wk5	Wk7	Wk9	E5 vs E7	E5 vs E9	E7 vs E9	L5vs L7	L5 vs L9	L7 vs L9	E5 vs L5	E7 vs L7	E9 vs L9	(E7-E5) vs (L9-L7)
Cell cycle - Caulobacter	0.52	0.55	0.55	0.52	0.53	0.54	0.60	0.87	0.99	0.99	0.84	0.95	0.99	0.35	1.00	0.99
Bacterial chemotaxis	0.26	0.37	0.41	0.25	0.25	0.37	0.02	0.00	0.99	0.99	0.01	0.05	0.99	0.01	1.00	0.99
Bacterial motility proteins	0.34	0.75	0.87	0.33	0.36	0.76	0.00	0.00	0.99	0.99	0.00	0.02	0.99	0.01	1.00	0.99
Cytoskeleton proteins	0.39	0.37	0.38	0.39	0.38	0.37	0.13	0.25	0.99	0.99	0.06	0.29	0.99	0.50	1.00	0.99
Flagellar assembly	0.10	0.31	0.36	0.10	0.12	0.30	0.00	0.00	0.99	0.99	0.00	0.01	0.99	0.00	1.00	0.99
Lysosome	0.13	0.15	0.16	0.14	0.16	0.16	0.58	0.45	0.99	0.99	0.81	0.86	0.99	0.73	1.00	0.99
Peroxisome	0.17	0.20	0.20	0.17	0.18	0.19	0.13	0.10	0.99	0.99	0.62	0.35	0.99	0.23	1.00	0.99
ABC transporters	3.41	2.82	2.84	3.37	3.21	2.88	0.01	0.01	0.99	0.99	0.05	0.19	0.99	0.09	1.00	0.99
Bacterial secretion system	0.54	0.62	0.61	0.56	0.55	0.61	0.03	0.04	0.99	0.99	0.18	0.05	0.99	0.04	1.00	0.99
Phosphotransferase system (PTS)	0.63	0.33	0.33	0.53	0.48	0.36	0.00	0.00	0.99	0.99	0.03	0.17	0.99	0.05	1.00	0.99
Secretion system	0.95	1.12	1.16	0.96	0.95	1.13	0.01	0.00	0.99	0.99	0.02	0.01	0.99	0.00	1.00	0.99
Transporters	7.11	5.72	5.73	6.99	6.66	5.83	0.00	0.00	0.99	0.99	0.02	0.12	0.99	0.04	1.00	0.99
Phosphatidylinositol signaling system	0.09	0.09	0.09	0.10	0.10	0.09	0.35	0.46	0.99	0.99	0.25	0.35	0.99	0.17	1.00	0.99
Two-component system	1.18	1.23	1.27	1.21	1.18	1.26	0.83	0.30	0.99	0.99	0.85	0.34	0.99	0.66	1.00	0.99
Bacterial toxins	0.15	0.12	0.13	0.14	0.14	0.13	0.00	0.00	0.99	0.99	0.03	0.05	0.99	0.00	1.00	0.99
Chaperones and folding catalysts	1.01	1.09	1.08	1.02	1.06	1.08	0.14	0.32	0.99	0.99	0.76	0.91	0.99	0.98	1.00	0.99
Protein export	0.61	0.66	0.67	0.60	0.62	0.67	0.01	0.00	0.99	0.99	0.00	0.02	0.99	0.05	1.00	0.99
RNA degradation	0.48	0.51	0.51	0.47	0.48	0.51	0.14	0.22	0.99	0.99	0.15	0.19	0.99	0.15	1.00	0.99
Sulfur relay system	0.26	0.22	0.22	0.25	0.25	0.23	0.01	0.02	0.99	0.99	0.10	0.19	0.99	0.03	1.00	0.99
Base excision repair	0.45	0.44	0.44	0.45	0.45	0.44	0.21	0.15	0.99	0.99	0.64	0.34	0.99	0.40	1.00	0.99
Chromosome	1.56	1.66	1.64	1.55	1.56	1.63	0.10	0.29	0.99	0.99	0.45	0.21	0.99	0.02	1.00	0.99
DNA repair and recombination proteins	2.89	3.02	2.97	2.87	2.88	2.99	0.74	0.94	0.99	0.99	0.81	0.59	0.99	0.26	1.00	0.99
DNA replication	0.69	0.75	0.74	0.69	0.70	0.74	0.15	0.34	0.99	0.99	0.19	0.18	0.99	0.07	1.00	0.99
DNA replication proteins	1.29	1.40	1.37	1.28	1.29	1.38	0.10	0.40	0.99	0.99	0.25	0.19	0.99	0.04	1.00	0.99
Homologous recombination	0.96	1.05	1.04	0.96	0.97	1.03	0.02	0.02	0.99	0.99	0.06	0.06	0.99	0.01	1.00	0.99
Mismatch repair	0.85	0.90	0.89	0.84	0.84	0.88	0.18	0.35	0.99	0.99	0.26	0.17	0.99	0.01	1.00	0.99
Nucleotide excision repair	0.40	0.42	0.42	0.39	0.40	0.41	0.75	0.93	0.99	0.99	0.63	0.62	0.99	0.31	1.00	0.99
RNA polymerase	0.17	0.18	0.18	0.17	0.17	0.18	0.73	0.98	0.99	0.99	0.17	0.59	0.99	0.88	1.00	0.99
Transcription factors	1.76	1.44	1.42	1.73	1.63	1.43	0.00	0.00	0.99	0.99	0.00	0.07	0.99	0.06	1.00	0.99
Transcription machinery	1.00	1.08	1.08	1.02	1.04	1.07	0.05	0.02	0.99	0.99	0.38	0.84	0.99	0.61	1.00	0.99
Aminoacyl-tRNA biosynthesis	1.18	1.26	1.25	1.16	1.19	1.25	0.57	0.56	0.99	0.99	0.27	0.59	0.99	0.50	1.00	0.99
Ribosome	2.41	2.64	2.62	2.37	2.44	2.62	0.07	0.07	0.99	0.99	0.03	0.18	0.99	0.07	1.00	0.99
Ribosome Biogenesis	1.40	1.49	1.47	1.40	1.40	1.47	0.38	0.54	0.99	0.99	0.53	0.37	0.99	0.19	1.00	0.99

RNA transport	0.15	0.13	0.14	0.15	0.15	0.14	0.02	0.12	0.99	0.99	0.38	0.18	0.99	0.04	1.00	0.99
Translation factors	0.55	0.60	0.60	0.54	0.56	0.59	0.02	0.01	0.99	0.99	0.00	0.14	0.99	0.05	1.00	0.99
Epithelial cell signaling in Helicobacter pylori infection	0.10	0.09	0.09	0.10	0.10	0.09	0.19	0.21	0.99	0.99	0.15	0.10	0.99	0.19	1.00	0.99
Tuberculosis	0.15	0.16	0.16	0.15	0.15	0.15	0.48	0.56	0.99	0.99	0.98	0.44	0.99	0.05	1.00	0.99
Vibrio cholerae pathogenic cycle	0.07	0.08	0.07	0.07	0.07	0.07	0.19	0.94	0.99	0.99	0.76	0.14	0.99	0.01	1.00	0.99
Alanine, aspartate and glutamate metabolism	1.12	1.13	1.13	1.14	1.13	1.12	0.14	0.29	0.99	0.99	0.00	0.10	0.99	0.13	1.00	0.99
Amino acid related enzymes	1.51	1.57	1.56	1.51	1.53	1.57	0.92	0.94	0.99	0.99	0.81	1.00	0.99	1.00	1.00	0.99
Arginine and proline metabolism	1.26	1.21	1.22	1.29	1.28	1.21	0.05	0.12	0.99	0.99	0.00	0.03	0.99	0.02	1.00	0.99
Cysteine and methionine metabolism	0.92	0.95	0.94	0.91	0.89	0.93	0.95	0.91	0.99	0.99	0.81	0.24	0.99	0.05	1.00	0.99
Glycine, serine and threonine metabolism	0.82	0.83	0.81	0.83	0.82	0.81	0.14	0.02	0.99	0.99	0.03	0.37	0.99	0.62	1.00	0.99
Histidine metabolism	0.67	0.67	0.68	0.67	0.68	0.66	0.23	0.81	0.99	0.99	0.11	0.18	0.99	0.22	1.00	0.99
Lysine biosynthesis	0.91	0.82	0.82	0.91	0.88	0.80	0.00	0.01	0.99	0.99	0.00	0.05	0.99	0.09	1.00	0.99
Lysine degradation	0.10	0.11	0.11	0.10	0.11	0.12	0.28	0.29	0.99	0.99	0.47	0.52	0.99	1.00	1.00	0.99
Phenylalanine metabolism	0.17	0.18	0.19	0.17	0.17	0.19	0.19	0.07	0.99	0.99	0.30	0.34	0.99	0.61	1.00	0.99
Phenylalanine, tyrosine and tryptophan biosynthesis	0.87	0.86	0.88	0.88	0.87	0.86	0.27	0.80	0.99	0.99	0.47	0.54	0.99	0.55	1.00	0.99
Tryptophan metabolism	0.11	0.12	0.13	0.11	0.11	0.13	0.76	0.16	0.99	0.99	0.25	0.19	0.99	0.69	1.00	0.99
Tyrosine metabolism	0.33	0.32	0.32	0.33	0.33	0.33	0.02	0.02	0.99	0.99	0.03	0.52	0.99	0.19	1.00	0.99
Valine, leucine and isoleucine biosynthesis	0.80	0.75	0.75	0.81	0.78	0.73	0.13	0.16	0.99	0.99	0.02	0.15	0.99	0.30	1.00	0.99
Valine, leucine and isoleucine degradation	0.17	0.20	0.20	0.18	0.18	0.21	0.15	0.24	0.99	0.99	0.17	0.16	0.99	0.41	1.00	0.99
Novobiocin biosynthesis	0.13	0.14	0.14	0.14	0.13	0.14	0.96	0.81	0.99	0.99	0.48	0.52	0.99	0.31	1.00	0.99
Phenylpropanoid biosynthesis	0.18	0.19	0.19	0.18	0.19	0.19	1.00	0.98	0.99	0.99	0.95	0.53	0.99	0.61	1.00	0.99
Streptomycin biosynthesis	0.35	0.35	0.35	0.35	0.37	0.35	0.60	0.88	0.99	0.99	0.58	0.16	0.99	0.07	1.00	0.99
Tropane, piperidine and pyridine alkaloid biosynthesis	0.11	0.12	0.12	0.11	0.11	0.12	0.24	0.32	0.99	0.99	0.81	0.17	0.99	0.13	1.00	0.99
Amino sugar and nucleotide sugar metabolism	1.59	1.53	1.52	1.59	1.62	1.53	0.00	0.00	0.99	0.99	0.01	0.01	0.99	0.00	1.00	0.99
Ascorbate and aldarate metabolism	0.13	0.11	0.10	0.12	0.11	0.11	0.00	0.00	0.99	0.99	0.45	0.67	0.99	0.40	1.00	0.99
Butanoate metabolism	0.57	0.59	0.61	0.57	0.57	0.62	0.92	0.22	0.99	0.99	0.05	0.02	0.99	0.55	1.00	0.99
C5-Branched dibasic acid metabolism	0.34	0.30	0.31	0.35	0.33	0.29	0.03	0.05	0.99	0.99	0.00	0.07	0.99	0.13	1.00	0.99
Citrate cycle (TCA cycle)	0.55	0.68	0.69	0.57	0.59	0.70	0.00	0.00	0.99	0.99	0.00	0.03	0.99	0.09	1.00	0.99
Fructose and mannose metabolism	1.16	0.99	0.97	1.15	1.17	1.00	0.00	0.00	0.99	0.99	0.00	0.00	0.99	0.00	1.00	0.99
Galactose metabolism	0.92	0.78	0.78	0.91	0.90	0.77	0.00	0.00	0.99	0.99	0.00	0.01	0.99	0.00	1.00	0.99
Glycolysis / Gluconeogenesis	1.15	1.13	1.12	1.15	1.15	1.15	0.08	0.07	0.99	0.99	0.48	0.55	0.99	0.30	1.00	0.99
Glyoxylate and dicarboxylate metabolism	0.52	0.48	0.48	0.53	0.52	0.49	0.00	0.00	0.99	0.99	0.00	0.03	0.99	0.01	1.00	0.99
Inositol phosphate metabolism	0.10	0.10	0.09	0.10	0.10	0.10	0.36	0.46	0.99	0.99	0.58	0.34	0.99	0.02	1.00	0.99
Pentose and glucuronate interconversions	0.61	0.55	0.53	0.63	0.61	0.55	0.00	0.00	0.99	0.99	0.00	0.01	0.99	0.00	1.00	0.99
Pentose phosphate pathway	0.93	0.87	0.86	0.94	0.92	0.88	0.00	0.00	0.99	0.99	0.02	0.13	0.99	0.02	1.00	0.99
Propanoate metabolism	0.48	0.47	0.48	0.47	0.47	0.49	0.31	0.88	0.99	0.99	0.83	0.68	0.99	0.79	1.00	0.99

Pyruvate metabolism	1.07	1.05	1.07	1.07	1.09	1.08	0.05	0.29	0.99	0.99	0.81	0.20	0.99	0.01	1.00	0.99
Starch and sucrose metabolism	1.14	1.07	1.06	1.15	1.14	1.06	0.01	0.00	0.99	0.99	0.00	0.01	0.99	0.01	1.00	0.99
Carbon fixation in photosynthetic organisms	0.66	0.68	0.68	0.66	0.67	0.69	0.66	0.86	0.99	0.99	0.68	0.98	0.99	0.40	1.00	0.99
Carbon fixation pathways in prokaryotes	0.96	1.06	1.08	0.98	1.00	1.07	0.01	0.00	0.99	0.99	0.03	0.10	0.99	0.19	1.00	0.99
Methane metabolism	1.40	1.28	1.34	1.40	1.40	1.33	0.01	0.16	0.99	0.99	0.19	0.14	0.99	0.01	1.00	0.99
Nitrogen metabolism	0.68	0.70	0.72	0.68	0.68	0.71	0.98	0.54	0.99	0.99	0.98	0.64	0.99	0.99	1.00	0.99
Oxidative phosphorylation	1.18	1.20	1.21	1.18	1.22	1.18	0.92	1.00	0.99	0.99	0.76	0.35	0.99	0.46	1.00	0.99
Photosynthesis	0.43	0.39	0.38	0.42	0.43	0.39	0.18	0.07	0.99	0.99	0.16	0.12	0.99	0.15	1.00	0.99
Photosynthesis proteins	0.43	0.40	0.38	0.43	0.44	0.39	0.18	0.07	0.99	0.99	0.16	0.12	0.99	0.15	1.00	0.99
Sulfur metabolism	0.25	0.26	0.26	0.25	0.26	0.26	0.92	0.81	0.99	0.99	0.81	0.67	0.99	0.46	1.00	0.99
Peptidases	1.89	1.95	1.94	1.88	1.89	1.93	0.75	0.80	0.99	0.99	0.97	0.91	0.99	0.67	1.00	0.99
Protein kinases	0.30	0.25	0.26	0.31	0.30	0.26	0.00	0.01	0.99	0.99	0.00	0.02	0.99	0.00	1.00	0.99
Glycosaminoglycan degradation	0.09	0.12	0.12	0.10	0.12	0.12	0.49	0.43	0.99	0.99	0.71	0.91	0.99	0.85	1.00	0.99
Glycosphingolipid biosynthesis - ganglio series	0.06	0.08	0.09	0.07	0.08	0.09	0.18	0.15	0.99	0.99	0.40	0.86	0.99	1.00	1.00	0.99
Glycosphingolipid biosynthesis - globo series	0.12	0.14	0.15	0.13	0.14	0.14	0.45	0.25	0.99	0.99	0.84	0.87	0.99	0.98	1.00	0.99
Glycosyltransferases	0.31	0.34	0.32	0.31	0.32	0.33	0.19	0.88	0.99	0.99	0.59	0.95	0.99	0.62	1.00	0.99
Lipopolysaccharide biosynthesis	0.16	0.29	0.27	0.18	0.20	0.28	0.01	0.02	0.99	0.99	0.03	0.14	0.99	0.13	1.00	0.99
Lipopolysaccharide biosynthesis proteins	0.28	0.41	0.38	0.30	0.33	0.40	0.02	0.06	0.99	0.99	0.08	0.28	0.99	0.30	1.00	0.99
Other glycan degradation	0.37	0.38	0.38	0.39	0.41	0.38	0.95	0.97	0.99	0.99	0.71	0.38	0.99	0.32	1.00	0.99
Peptidoglycan biosynthesis	0.86	0.86	0.85	0.86	0.84	0.83	0.43	0.26	0.99	0.99	0.19	0.52	0.99	1.00	1.00	0.99
Biosynthesis of unsaturated fatty acids	0.10	0.10	0.10	0.10	0.10	0.10	1.00	0.81	0.99	0.99	0.47	0.84	0.99	0.43	1.00	0.99
Fatty acid biosynthesis	0.48	0.48	0.49	0.48	0.49	0.48	0.59	0.98	0.99	0.99	0.72	0.35	0.99	0.38	1.00	0.99
Fatty acid metabolism	0.21	0.21	0.20	0.21	0.20	0.21	0.59	0.33	0.99	0.99	0.48	0.59	0.99	1.00	1.00	0.99
Glycerolipid metabolism	0.40	0.34	0.35	0.38	0.37	0.36	0.00	0.02	0.99	0.99	0.43	0.39	0.99	0.12	1.00	0.99
Glycerophospholipid metabolism	0.55	0.54	0.54	0.54	0.54	0.54	0.18	0.56	0.99	0.99	0.82	0.64	0.99	0.42	1.00	0.99
Linoleic acid metabolism	0.09	0.08	0.08	0.09	0.09	0.08	0.00	0.00	0.99	0.99	0.45	0.02	0.99	0.00	1.00	0.99
Lipid biosynthesis proteins	0.54	0.62	0.63	0.54	0.56	0.62	0.00	0.00	0.99	0.99	0.00	0.05	0.99	0.02	1.00	0.99
Sphingolipid metabolism	0.25	0.25	0.25	0.25	0.26	0.24	0.83	0.88	0.99	0.99	0.50	0.38	0.99	0.50	1.00	0.99
Biotin metabolism	0.17	0.16	0.17	0.18	0.19	0.17	0.23	0.74	0.99	0.99	0.26	0.12	0.99	0.02	1.00	0.99
Folate biosynthesis	0.41	0.42	0.41	0.41	0.42	0.42	0.79	0.62	0.99	0.99	0.76	0.37	0.99	0.35	1.00	0.99
Nicotinate and nicotinamide metabolism	0.44	0.47	0.46	0.44	0.44	0.46	0.51	0.88	0.99	0.99	0.90	0.40	0.99	0.19	1.00	0.99
One carbon pool by folate	0.61	0.70	0.70	0.60	0.61	0.68	0.00	0.00	0.99	0.99	0.00	0.01	0.99	0.00	1.00	0.99
Pantothenate and CoA biosynthesis	0.65	0.66	0.66	0.65	0.65	0.64	0.72	0.93	0.99	0.99	0.12	0.33	0.99	1.00	1.00	0.99
Porphyrin and chlorophyll metabolism	0.98	0.80	0.77	1.02	0.98	0.81	0.00	0.00	0.99	0.99	0.00	0.01	0.99	0.00	1.00	0.99
Riboflavin metabolism	0.22	0.24	0.23	0.22	0.23	0.23	0.18	0.79	0.99	0.99	0.76	0.85	0.99	0.55	1.00	0.99
Thiamine metabolism	0.52	0.51	0.52	0.52	0.52	0.51	0.14	0.60	0.99	0.99	0.16	0.34	0.99	0.29	1.00	0.99

Ubiquinone and other terpenoid-quinone biosynthesis	0.12	0.20	0.18	0.13	0.14	0.19	0.02	0.07	0.99	0.99	0.09	0.19	0.99	0.13	1.00	0.99
Vitamin B6 metabolism	0.19	0.23	0.22	0.20	0.19	0.22	0.01	0.02	0.99	0.99	0.36	0.11	0.99	0.01	1.00	0.99
beta-Alanine metabolism	0.18	0.18	0.18	0.19	0.19	0.18	0.82	0.97	0.99	0.99	0.81	0.25	0.99	0.19	1.00	0.99
Cyanoamino acid metabolism	0.31	0.32	0.32	0.31	0.32	0.32	0.94	0.81	0.99	0.99	0.97	0.63	0.99	0.59	1.00	0.99
D-Alanine metabolism	0.10	0.10	0.11	0.10	0.10	0.10	0.52	0.97	0.99	0.99	0.98	0.46	0.99	0.43	1.00	0.99
D-Glutamine and D-glutamate metabolism	0.15	0.16	0.16	0.15	0.15	0.16	0.23	0.07	0.99	0.99	0.71	0.59	0.99	0.30	1.00	0.99
Glutathione metabolism	0.16	0.19	0.18	0.17	0.17	0.19	0.08	0.73	0.99	0.99	0.36	0.37	0.99	0.26	1.00	0.99
Selenocompound metabolism	0.37	0.36	0.36	0.36	0.38	0.37	0.02	0.01	0.99	0.99	0.98	0.37	0.99	0.05	1.00	0.99
Taurine and hypotaurine metabolism	0.10	0.12	0.12	0.11	0.11	0.12	0.01	0.06	0.99	0.99	0.10	0.55	0.99	0.26	1.00	0.99
Biosynthesis of ansamycins	0.11	0.11	0.12	0.11	0.11	0.12	0.59	1.00	0.99	0.99	0.48	0.16	0.99	0.61	1.00	0.99
Polyketide sugar unit biosynthesis	0.23	0.22	0.22	0.23	0.25	0.22	0.18	0.39	0.99	0.99	0.50	0.07	0.99	0.01	1.00	0.99
Prenyltransferases	0.30	0.35	0.34	0.31	0.31	0.34	0.01	0.01	0.99	0.99	0.11	0.16	0.99	0.01	1.00	0.99
Terpenoid backbone biosynthesis	0.56	0.60	0.61	0.56	0.56	0.59	0.16	0.05	0.99	0.99	0.25	0.15	0.99	0.01	1.00	0.99
Tetracycline biosynthesis	0.17	0.12	0.13	0.16	0.16	0.13	0.00	0.00	0.99	0.99	0.01	0.02	0.99	0.00	1.00	0.99
Purine metabolism	2.30	2.31	2.28	2.27	2.29	2.31	0.15	0.04	0.99	0.99	0.65	0.65	0.99	0.66	1.00	0.99
Pyrimidine metabolism	1.88	1.99	1.98	1.87	1.88	1.99	0.45	0.56	0.99	0.99	0.31	0.19	0.99	0.13	1.00	0.99
Aminobenzoate degradation	0.10	0.11	0.11	0.10	0.11	0.12	1.00	0.80	0.99	0.99	0.23	0.34	0.99	0.61	1.00	0.99
Benzoate degradation	0.18	0.18	0.20	0.18	0.17	0.20	0.81	0.68	0.99	0.99	0.71	0.19	0.99	0.62	1.00	0.99
Bisphenol degradation	0.10	0.09	0.09	0.10	0.10	0.09	0.00	0.00	0.99	0.99	0.10	0.03	0.99	0.00	1.00	0.99
Chloroalkane and chloroalkene degradation	0.22	0.18	0.19	0.22	0.22	0.20	0.00	0.02	0.99	0.99	0.23	0.14	0.99	0.01	1.00	0.99
Drug metabolism - other enzymes	0.32	0.35	0.34	0.32	0.32	0.34	0.15	0.39	0.99	0.99	0.76	0.14	0.99	0.03	1.00	0.99
Naphthalene degradation	0.14	0.14	0.14	0.14	0.14	0.15	0.74	0.97	0.99	0.99	0.98	0.35	0.99	0.88	1.00	0.99
Nitrotoluene degradation	0.10	0.07	0.08	0.10	0.10	0.09	0.09	0.24	0.99	0.99	0.80	0.52	0.99	0.08	1.00	0.99
Polycyclic aromatic hydrocarbon degradation	0.12	0.11	0.11	0.11	0.12	0.11	0.00	0.00	0.99	0.99	0.00	0.01	0.99	0.00	1.00	0.99
Toluene degradation	0.08	0.10	0.10	0.09	0.09	0.10	0.49	0.37	0.99	0.99	0.73	0.52	0.99	0.93	1.00	0.99
Adipocytokine signaling pathway	0.06	0.09	0.09	0.07	0.07	0.09	0.00	0.01	0.99	0.99	0.30	0.26	0.99	0.10	1.00	0.99
Insulin signaling pathway	0.08	0.08	0.09	0.08	0.08	0.09	0.88	0.88	0.99	0.99	0.98	0.95	0.99	0.99	1.00	0.99
PPAR signaling pathway	0.10	0.12	0.12	0.11	0.11	0.12	0.01	0.02	0.99	0.99	0.58	0.35	0.99	0.19	1.00	0.99
Plant-pathogen interaction	0.14	0.15	0.15	0.14	0.14	0.15	0.18	0.05	0.99	0.99	0.03	0.19	0.99	0.19	1.00	0.99
Glutamatergic synapse	0.11	0.11	0.11	0.12	0.12	0.11	0.60	0.88	0.99	0.99	0.16	0.03	0.99	0.02	1.00	0.99
Cell division	0.10	0.08	0.09	0.10	0.10	0.08	0.07	0.10	0.99	0.99	0.06	0.03	0.99	0.01	1.00	0.99
Cell motility and secretion	0.14	0.18	0.17	0.14	0.15	0.17	0.00	0.00	0.99	0.99	0.01	0.03	0.99	0.02	1.00	0.99
Inorganic ion transport and metabolism	0.18	0.17	0.17	0.19	0.19	0.17	0.08	0.05	0.99	0.99	0.05	0.14	0.99	0.04	1.00	0.99
Membrane and intracellular structural molecules	0.49	0.63	0.61	0.52	0.58	0.61	0.02	0.07	0.99	0.99	0.23	0.66	0.99	0.61	1.00	0.99
Other ion-coupled transporters	1.20	1.18	1.12	1.22	1.20	1.16	0.10	0.00	0.99	0.99	0.01	0.32	0.99	0.32	1.00	0.99
Other transporters	0.26	0.26	0.26	0.26	0.26	0.26	0.13	0.17	0.99	0.99	0.39	0.55	0.99	0.35	1.00	0.99

Pores ion channels	0.28	0.38	0.36	0.30	0.33	0.37	0.02	0.07	0.99	0.99	0.15	0.49	0.99	0.44	1.00	0.99
Signal transduction mechanisms	0.48	0.43	0.43	0.48	0.46	0.42	0.02	0.01	0.99	0.99	0.01	0.18	0.99	0.22	1.00	0.99
Sporulation	0.78	0.63	0.67	0.77	0.74	0.64	0.14	0.38	0.99	0.99	0.40	0.34	0.99	0.31	1.00	0.99
Protein folding and associated processing	0.64	0.62	0.61	0.65	0.65	0.61	0.02	0.01	0.99	0.99	0.03	0.05	0.99	0.02	1.00	0.99
Replication, recombination and repair proteins	0.81	0.77	0.76	0.81	0.78	0.75	0.08	0.09	0.99	0.99	0.03	0.36	0.99	0.61	1.00	0.99
Restriction enzyme	0.17	0.23	0.23	0.18	0.18	0.22	0.00	0.00	0.99	0.99	0.03	0.10	0.99	0.02	1.00	0.99
Translation proteins	0.87	0.94	0.94	0.86	0.86	0.94	0.06	0.02	0.99	0.99	0.01	0.01	0.99	0.00	1.00	0.99
Amino acid metabolism	0.25	0.20	0.20	0.24	0.24	0.20	0.00	0.00	0.99	0.99	0.01	0.02	0.99	0.02	1.00	0.99
Carbohydrate metabolism	0.19	0.17	0.16	0.19	0.18	0.16	0.10	0.01	0.99	0.99	0.02	0.09	0.99	0.26	1.00	0.99
Energy metabolism	0.85	0.97	0.99	0.87	0.87	0.96	0.00	0.00	0.99	0.99	0.03	0.04	0.99	0.02	1.00	0.99
Lipid metabolism	0.14	0.13	0.13	0.14	0.14	0.13	0.95	1.00	0.99	0.99	0.98	0.43	0.99	0.46	1.00	0.99
Metabolism of cofactors and vitamins	0.11	0.09	0.09	0.11	0.11	0.10	0.14	0.04	0.99	0.99	0.30	0.14	0.99	0.04	1.00	0.99
Others	0.89	0.84	0.82	0.89	0.87	0.83	0.03	0.01	0.99	0.99	0.05	0.19	0.99	0.20	1.00	0.99
Function unknown	1.21	1.23	1.25	1.21	1.24	1.26	0.59	0.98	0.99	0.99	0.89	0.95	0.99	0.22	1.00	0.99
General function prediction only	3.70	3.65	3.65	3.69	3.75	3.63	0.00	0.00	0.99	0.99	0.00	0.01	0.99	0.00	1.00	0.99
Apoptosis	0.00	0.00	0.00	0.00	0.00	0.00	0.15	0.03	0.99	0.99	0.23	0.35	0.99	0.80	1.00	0.99
Cell cycle	0.00	0.00	0.00	0.00	0.00	0.00	0.61	0.02	0.99	0.99	0.00	0.21	0.99	1.00	1.00	0.99
Meiosis - yeast	0.00	0.00	0.00	0.00	0.00	0.00	0.67	0.94	0.99	0.99	0.98	0.40	0.99	0.03	1.00	0.99
p53 signaling pathway	0.00	0.00	0.00	0.00	0.00	0.00	0.14	0.16	0.99	0.99	0.75	0.59	0.99	0.19	1.00	0.99
Phagosome	0.00	0.00	0.00	0.00	0.00	0.00	0.61	0.02	0.99	0.99	0.00	0.21	0.99	1.00	1.00	0.99
Calcium signaling pathway	0.00	0.00	0.00	0.00	0.00	0.00	1.00	0.98	0.99	0.99	0.98	1.00	0.99	1.00	1.00	0.99
MAPK signaling pathway - yeast	0.06	0.06	0.06	0.06	0.06	0.06	0.49	0.97	0.99	0.99	0.95	0.59	0.99	0.19	1.00	0.99
mTOR signaling pathway	0.00	0.00	0.00	0.00	0.00	0.00	0.61	0.02	0.99	0.99	0.00	0.21	0.99	1.00	1.00	0.99
Cellular antigens	0.03	0.05	0.05	0.04	0.04	0.05	0.07	0.20	0.99	0.99	0.45	0.49	0.99	0.32	1.00	0.99
Ion channels	0.01	0.01	0.01	0.01	0.01	0.01	0.06	0.01	0.99	0.99	0.98	0.59	0.99	1.00	1.00	0.99
Proteasome	0.05	0.05	0.05	0.05	0.04	0.05	0.58	0.45	0.99	0.99	0.98	0.59	0.99	0.19	1.00	0.99
Protein processing in endoplasmic reticulum	0.06	0.08	0.08	0.06	0.07	0.08	0.01	0.00	0.99	0.99	0.01	0.34	0.99	0.22	1.00	0.99
Ubiquitin system	0.01	0.01	0.01	0.01	0.01	0.01	0.29	0.07	0.99	0.99	0.05	0.19	0.99	0.29	1.00	0.99
Non-homologous end-joining	0.00	0.01	0.01	0.00	0.01	0.01	0.58	0.74	0.99	0.99	0.42	0.64	0.99	0.93	1.00	0.99
Basal transcription factors	0.00	0.00	0.00	0.00	0.00	0.00	0.92	0.30	0.99	0.99	0.20	0.55	0.99	0.61	1.00	0.99
mRNA surveillance pathway	0.00	0.00	0.00	0.00	0.00	0.00	0.59	0.02	0.99	0.99	0.00	0.23	0.99	1.00	1.00	0.99
Ribosome biogenesis in eukaryotes	0.05	0.05	0.05	0.05	0.05	0.06	0.23	0.20	0.99	0.99	0.09	0.07	0.99	0.10	1.00	0.99
Bladder cancer	0.00	0.00	0.00	0.00	0.00	0.00	0.07	0.02	0.99	0.99	0.47	0.35	0.99	0.08	1.00	0.99
Colorectal cancer	0.00	0.00	0.00	0.00	0.00	0.00	0.14	0.16	0.99	0.99	0.75	0.59	0.99	0.19	1.00	0.99
Pathways in cancer	0.05	0.05	0.06	0.04	0.05	0.06	0.02	0.00	0.99	0.99	0.00	0.19	0.99	0.22	1.00	0.99
Prostate cancer	0.04	0.05	0.05	0.04	0.04	0.05	0.10	0.02	0.99	0.99	0.09	0.74	0.99	0.52	1.00	0.99

Renal cell carcinoma	0.00	0.01	0.01	0.00	0.00	0.01	0.01	0.00	0.99	0.99	0.00	0.05	0.99	0.17	1.00	0.99
Small cell lung cancer	0.00	0.00	0.00	0.00	0.00	0.00	0.14	0.16	0.99	0.99	0.75	0.59	0.99	0.19	1.00	0.99
Hypertrophic cardiomyopathy (HCM)	0.00	0.00	0.00	0.00	0.00	0.00	1.00	1.00	0.99	0.99	0.98	1.00	0.99	1.00	1.00	0.99
Viral myocarditis	0.00	0.00	0.00	0.00	0.00	0.00	0.14	0.16	0.99	0.99	0.75	0.59	0.99	0.19	1.00	0.99
Primary immunodeficiency	0.05	0.05	0.04	0.04	0.04	0.04	0.28	0.02	0.99	0.99	0.81	0.69	0.99	0.46	1.00	0.99
Systemic lupus erythematosus	0.00	0.00	0.00	0.00	0.00	0.00	1.00	0.94	0.99	0.99	0.98	1.00	0.99	1.00	1.00	0.99
African trypanosomiasis	0.00	0.00	0.00	0.00	0.00	0.00	0.03	0.01	0.99	0.99	0.00	0.53	0.99	0.99	1.00	0.99
Amoebiasis	0.02	0.01	0.01	0.02	0.02	0.01	0.14	0.40	0.99	0.99	0.58	0.14	0.99	0.02	1.00	0.99
Bacterial invasion of epithelial cells	0.00	0.00	0.00	0.00	0.00	0.00	0.07	0.03	0.99	0.99	0.12	0.01	0.99	0.02	1.00	0.99
Chagas disease (American trypanosomiasis)	0.00	0.00	0.00	0.00	0.00	0.00	0.00	0.00	0.99	0.99	0.00	0.06	0.99	0.26	1.00	0.99
Hepatitis C	0.00	0.00	0.00	0.00	0.00	0.00	0.61	0.02	0.99	0.99	0.00	0.21	0.99	1.00	1.00	0.99
Influenza A	0.00	0.00	0.00	0.00	0.00	0.00	0.20	0.05	0.99	0.99	0.08	0.31	0.99	0.50	1.00	0.99
Measles	0.00	0.00	0.00	0.00	0.00	0.00	0.61	0.02	0.99	0.99	0.00	0.21	0.99	1.00	1.00	0.99
Pathogenic Escherichia coli infection	0.00	0.00	0.00	0.00	0.00	0.00	1.00	1.00	0.99	0.99	0.98	1.00	0.99	1.00	1.00	0.99
Pertussis	0.02	0.03	0.03	0.03	0.03	0.03	0.14	0.40	0.99	0.99	0.62	0.57	0.99	0.63	1.00	0.99
Shigellosis	0.00	0.00	0.00	0.00	0.00	0.00	1.00	1.00	0.99	0.99	0.98	1.00	0.99	1.00	1.00	0.99
Staphylococcus aureus infection	0.01	0.01	0.01	0.01	0.02	0.02	0.07	0.76	0.99	0.99	0.07	0.74	0.99	0.04	1.00	0.99
Toxoplasmosis	0.00	0.00	0.00	0.00	0.00	0.00	0.14	0.16	0.99	0.99	0.75	0.59	0.99	0.19	1.00	0.99
Vibrio cholerae infection	0.00	0.00	0.00	0.00	0.00	0.00	0.61	0.02	0.99	0.99	0.00	0.19	0.99	1.00	1.00	0.99
Type I diabetes mellitus	0.05	0.06	0.06	0.05	0.05	0.06	0.03	0.00	0.99	0.99	0.24	0.59	0.99	0.44	1.00	0.99
Type II diabetes mellitus	0.05	0.05	0.05	0.05	0.05	0.05	0.45	0.88	0.99	0.99	0.69	0.84	0.99	0.40	1.00	0.99
Alzheimer's disease	0.05	0.05	0.05	0.05	0.05	0.05	0.22	0.20	0.99	0.99	0.15	0.66	0.99	0.61	1.00	0.99
Amyotrophic lateral sclerosis (ALS)	0.01	0.01	0.01	0.01	0.01	0.01	0.37	0.98	0.99	0.99	0.81	0.23	0.99	0.61	1.00	0.99
Huntington's disease	0.02	0.03	0.03	0.02	0.03	0.03	0.18	0.17	0.99	0.99	0.03	0.21	0.99	0.37	1.00	0.99
Parkinson's disease	0.00	0.00	0.00	0.00	0.00	0.00	0.18	0.21	0.99	0.99	0.58	0.34	0.99	0.12	1.00	0.99
Prion diseases	0.01	0.00	0.00	0.00	0.00	0.00	0.00	0.01	0.99	0.99	0.08	0.34	0.99	0.03	1.00	0.99
beta-Lactam resistance	0.04	0.03	0.03	0.05	0.05	0.03	0.00	0.00	0.99	0.99	0.00	0.00	0.99	0.00	1.00	0.99
Betalain biosynthesis	0.00	0.00	0.00	0.00	0.00	0.00	1.00	0.94	0.99	0.99	0.97	1.00	0.99	1.00	1.00	0.99
Butirosin and neomycin biosynthesis	0.07	0.08	0.08	0.07	0.07	0.08	0.18	0.06	0.99	0.99	0.48	0.46	0.99	0.44	1.00	0.99
Caffeine metabolism	0.00	0.00	0.00	0.00	0.00	0.00	1.00	0.82	0.99	0.99	0.84	1.00	0.99	1.00	1.00	0.99
Flavone and flavonol biosynthesis	0.00	0.00	0.00	0.00	0.00	0.00	0.92	0.73	0.99	0.99	0.94	0.98	0.99	1.00	1.00	0.99
Flavonoid biosynthesis	0.00	0.00	0.00	0.00	0.00	0.01	0.92	0.84	0.99	0.99	0.14	0.24	0.99	0.98	1.00	0.99
Indole alkaloid biosynthesis	0.00	0.00	0.00	0.00	0.00	0.00	1.00	0.94	0.99	0.99	0.97	1.00	0.99	1.00	1.00	0.99
Isoquinoline alkaloid biosynthesis	0.05	0.06	0.05	0.06	0.05	0.06	0.78	0.53	0.99	0.99	0.83	0.98	0.99	1.00	1.00	0.99
Penicillin and cephalosporin biosynthesis	0.03	0.03	0.03	0.04	0.04	0.03	0.09	0.59	0.99	0.99	0.03	0.05	0.99	0.00	1.00	0.99
Stilbenoid, diarylheptanoid and gingerol biosynthesis	0.00	0.00	0.00	0.00	0.00	0.00	0.06	0.03	0.99	0.99	0.65	0.67	0.99	0.68	1.00	0.99

Photosynthesis - antenna proteins	0.00	0.00	0.00	0.00	0.00	0.00	1.00	0.98	0.99	0.99	0.98	1.00	0.99	1.00	1.00	0.99
Glycosphingolipid biosynthesis - lacto and neolacto series	0.00	0.00	0.00	0.00	0.00	0.00	0.15	0.10	0.99	0.99	0.81	0.86	0.99	0.98	1.00	0.99
N-Glycan biosynthesis	0.02	0.03	0.03	0.02	0.03	0.03	0.09	0.07	0.99	0.99	0.30	0.98	0.99	0.98	1.00	0.99
Various types of N-glycan biosynthesis	0.00	0.00	0.00	0.00	0.00	0.00	0.92	0.15	0.99	0.99	0.01	0.19	0.99	1.00	1.00	0.99
alpha-Linolenic acid metabolism	0.00	0.00	0.00	0.00	0.00	0.00	0.23	0.88	0.99	0.99	0.98	0.59	0.99	0.63	1.00	0.99
Arachidonic acid metabolism	0.01	0.03	0.02	0.01	0.01	0.02	0.00	0.01	0.99	0.99	0.00	0.05	0.99	0.01	1.00	0.99
Ether lipid metabolism	0.00	0.00	0.00	0.00	0.00	0.00	0.00	0.00	0.99	0.99	0.03	0.88	0.99	0.61	1.00	0.99
Fatty acid elongation in mitochondria	0.00	0.00	0.00	0.00	0.00	0.00	1.00	0.93	0.99	0.99	0.97	1.00	0.99	1.00	1.00	0.99
Primary bile acid biosynthesis	0.04	0.03	0.03	0.04	0.04	0.03	0.01	0.01	0.99	0.99	0.00	0.02	0.99	0.02	1.00	0.99
Secondary bile acid biosynthesis	0.04	0.03	0.03	0.04	0.04	0.03	0.01	0.01	0.99	0.99	0.00	0.02	0.99	0.02	1.00	0.99
Steroid biosynthesis	0.00	0.00	0.00	0.00	0.00	0.00	1.00	0.40	0.99	0.99	0.76	1.00	0.99	1.00	1.00	0.99
Steroid hormone biosynthesis	0.02	0.02	0.02	0.02	0.02	0.02	0.95	1.00	0.99	0.99	0.81	0.86	0.99	0.68	1.00	0.99
Synthesis and degradation of ketone bodies	0.02	0.02	0.02	0.02	0.02	0.02	0.88	0.98	0.99	0.99	0.16	0.14	0.99	0.61	1.00	0.99
Lipoic acid metabolism	0.03	0.05	0.05	0.03	0.04	0.05	0.08	0.10	0.99	0.99	0.13	0.55	0.99	0.61	1.00	0.99
Retinol metabolism	0.02	0.02	0.02	0.02	0.02	0.02	0.37	0.94	0.99	0.99	0.98	0.86	0.99	0.98	1.00	0.99
D-Arginine and D-ornithine metabolism	0.00	0.00	0.00	0.00	0.00	0.00	0.21	0.22	0.99	0.99	0.03	0.01	0.99	0.02	1.00	0.99
Phosphonate and phosphinate metabolism	0.06	0.06	0.06	0.06	0.06	0.06	0.41	0.97	0.99	0.99	0.98	0.86	0.99	0.22	1.00	0.99
Biosynthesis of siderophore group nonribosomal peptides	0.01	0.02	0.01	0.01	0.01	0.02	0.33	0.59	0.99	0.99	0.84	0.71	0.99	0.98	1.00	0.99
Biosynthesis of vancomycin group antibiotics	0.07	0.07	0.07	0.07	0.07	0.07	1.00	0.81	0.99	0.99	0.83	0.24	0.99	0.14	1.00	0.99
Carotenoid biosynthesis	0.00	0.00	0.00	0.00	0.00	0.00	0.59	1.00	0.99	0.99	0.81	0.98	0.99	1.00	1.00	0.99
Geraniol degradation	0.03	0.04	0.04	0.03	0.03	0.05	0.10	0.16	0.99	0.99	0.23	0.17	0.99	0.20	1.00	0.99
Limonene and pinene degradation	0.07	0.08	0.09	0.08	0.07	0.09	0.16	0.04	0.99	0.99	0.16	0.01	0.99	0.03	1.00	0.99
Zeatin biosynthesis	0.06	0.06	0.06	0.06	0.06	0.06	0.15	0.81	0.99	0.99	0.84	0.87	0.99	0.22	1.00	0.99
1,1,1-Trichloro-2,2-bis(4-chlorophenyl)ethane (DDT) degradation	0.00	0.00	0.00	0.00	0.00	0.00	0.19	0.00	0.99	0.99	0.01	0.27	0.99	0.76	1.00	0.99
Atrazine degradation	0.02	0.01	0.01	0.02	0.03	0.01	0.18	0.27	0.99	0.99	0.76	0.14	0.99	0.03	1.00	0.99
Caprolactam degradation	0.01	0.01	0.01	0.01	0.01	0.01	1.00	0.88	0.99	0.99	0.21	0.03	0.99	0.26	1.00	0.99
Chlorocyclohexane and chlorobenzene degradation	0.01	0.01	0.00	0.01	0.01	0.01	0.00	0.00	0.99	0.99	0.01	0.01	0.99	0.00	1.00	0.99
Dioxin degradation	0.06	0.05	0.05	0.06	0.06	0.05	0.02	0.06	0.99	0.99	0.19	0.19	0.99	0.19	1.00	0.99
Drug metabolism - cytochrome P450	0.02	0.02	0.02	0.02	0.02	0.02	1.00	0.98	0.99	0.99	0.98	0.84	0.99	0.86	1.00	0.99
Ethylbenzene degradation	0.03	0.05	0.05	0.04	0.03	0.05	0.01	0.01	0.99	0.99	0.03	0.03	0.99	0.05	1.00	0.99
Fluorobenzoate degradation	0.00	0.00	0.00	0.00	0.00	0.00	0.88	0.80	0.99	0.99	0.98	0.59	0.99	0.86	1.00	0.99
Metabolism of xenobiotics by cytochrome P450	0.02	0.02	0.02	0.02	0.02	0.02	1.00	0.98	0.99	0.99	0.98	0.85	0.99	0.85	1.00	0.99
Styrene degradation	0.02	0.01	0.01	0.02	0.02	0.02	0.00	0.03	0.99	0.99	0.95	0.25	0.99	0.00	1.00	0.99
Xylene degradation	0.06	0.05	0.05	0.06	0.05	0.05	0.03	0.15	0.99	0.99	0.48	0.34	0.99	0.20	1.00	0.99
Cardiac muscle contraction	0.00	0.00	0.00	0.00	0.00	0.00	0.23	0.22	0.99	0.99	0.58	0.34	0.99	0.15	1.00	0.99
Bile secretion	0.00	0.00	0.00	0.00	0.00	0.00	0.63	0.02	0.99	0.99	0.00	0.22	0.99	1.00	1.00	0.99

Carbohydrate digestion and absorption	0.01	0.02	0.01	0.01	0.01	0.01	0.03	0.22	0.99	0.99	0.04	0.59	0.99	0.10	1.00	0.99
Mineral absorption	0.00	0.01	0.01	0.00	0.00	0.01	0.01	0.01	0.99	0.99	0.01	0.02	0.99	0.01	1.00	0.99
Protein digestion and absorption	0.02	0.04	0.03	0.03	0.03	0.03	0.08	0.41	0.99	0.99	0.61	0.78	0.99	0.51	1.00	0.99
Progesterone-mediated oocyte maturation	0.04	0.05	0.05	0.04	0.04	0.05	0.08	0.01	0.99	0.99	0.05	0.59	0.99	0.40	1.00	0.99
Renin-angiotensin system	0.00	0.00	0.00	0.00	0.00	0.00	1.00	0.45	0.99	0.99	0.75	1.00	0.99	1.00	1.00	0.99
Circadian rhythm - plant	0.00	0.00	0.00	0.00	0.00	0.00	1.00	0.56	0.99	0.99	0.98	1.00	0.99	1.00	1.00	0.99
Aldosterone-regulated sodium reabsorption	0.00	0.00	0.00	0.00	0.00	0.00	1.00	0.98	0.99	0.99	0.98	1.00	0.99	1.00	1.00	0.99
Proximal tubule bicarbonate reclamation	0.01	0.02	0.03	0.02	0.02	0.02	0.01	0.00	0.99	0.99	0.08	0.35	0.99	0.40	1.00	0.99
Antigen processing and presentation	0.04	0.05	0.05	0.04	0.04	0.05	0.08	0.01	0.99	0.99	0.05	0.59	0.99	0.40	1.00	0.99
NOD-like receptor signaling pathway	0.04	0.05	0.05	0.04	0.05	0.05	0.09	0.04	0.99	0.99	0.09	0.86	0.99	0.69	1.00	0.99
RIG-I-like receptor signaling pathway	0.00	0.00	0.00	0.00	0.00	0.00	0.00	0.00	0.99	0.99	0.00	0.14	0.99	0.98	1.00	0.99
Electron transfer carriers	0.01	0.01	0.01	0.01	0.01	0.01	0.82	0.73	0.99	0.99	0.75	0.04	0.99	0.05	1.00	0.99
Germination	0.04	0.04	0.05	0.04	0.05	0.04	1.00	0.50	0.99	0.99	0.97	0.84	0.99	0.87	1.00	0.99
Transcription related proteins	0.01	0.00	0.00	0.00	0.00	0.00	0.00	0.06	0.99	0.99	0.81	0.70	0.99	0.15	1.00	0.99
Biosynthesis and biodegradation of secondary metabolites	0.06	0.05	0.05	0.07	0.06	0.05	0.01	0.01	0.99	0.99	0.00	0.01	0.99	0.00	1.00	0.99
Glycan biosynthesis and metabolism	0.02	0.04	0.04	0.02	0.02	0.04	0.00	0.00	0.99	0.99	0.03	0.05	0.99	0.03	1.00	0.99
Nucleotide metabolism	0.03	0.04	0.04	0.03	0.02	0.03	0.70	0.23	0.99	0.99	0.58	0.02	0.99	0.00	1.00	0.99
